# Catchet-MS identifies IKZF1-targeting thalidomide analogues as novel HIV-1 latency reversal agents

**DOI:** 10.1093/nar/gkac407

**Published:** 2022-05-30

**Authors:** Enrico Ne, Raquel Crespo, Ray Izquierdo-Lara, Shringar Rao, Selin Koçer, Alicja Górska, Thomas van Staveren, Tsung Wai Kan, David van de Vijver, Dick Dekkers, Casper Rokx, Panagiotis Moulos, Pantelis Hatzis, Robert-Jan Palstra, Jeroen Demmers, Tokameh Mahmoudi

**Affiliations:** Department of Biochemistry, Erasmus University Medical Center, Ee622 PO Box 2040, 3000CA Rotterdam, The Netherlands; Department of Biochemistry, Erasmus University Medical Center, Ee622 PO Box 2040, 3000CA Rotterdam, The Netherlands; Department of Biochemistry, Erasmus University Medical Center, Ee622 PO Box 2040, 3000CA Rotterdam, The Netherlands; Department of Biochemistry, Erasmus University Medical Center, Ee622 PO Box 2040, 3000CA Rotterdam, The Netherlands; Department of Biochemistry, Erasmus University Medical Center, Ee622 PO Box 2040, 3000CA Rotterdam, The Netherlands; Department of Biochemistry, Erasmus University Medical Center, Ee622 PO Box 2040, 3000CA Rotterdam, The Netherlands; Department of Biochemistry, Erasmus University Medical Center, Ee622 PO Box 2040, 3000CA Rotterdam, The Netherlands; Department of Biochemistry, Erasmus University Medical Center, Ee622 PO Box 2040, 3000CA Rotterdam, The Netherlands; Department of Pathology, Erasmus University Medical Center, The Netherlands; Department of Urology, Erasmus University Medical Center, The Netherlands; Department of Viroscience, Erasmus University Medical Center, The Netherlands; Proteomics Center, Erasmus University Medical Center, Ee679a PO Box 2040, 3000CA Rotterdam, The Netherlands; Department of Internal Medicine, Section of Infectious Diseases, Erasmus University Medical Center, Rg-530, PO Box 2040, 3000CA Rotterdam, The Netherlands; Institute for Fundamental Biomedical Research, Biomedical Sciences Research Center “Alexander Fleming”, 16672, Vari, Greece; Institute for Fundamental Biomedical Research, Biomedical Sciences Research Center “Alexander Fleming”, 16672, Vari, Greece; Department of Biochemistry, Erasmus University Medical Center, Ee622 PO Box 2040, 3000CA Rotterdam, The Netherlands; Department of Pathology, Erasmus University Medical Center, The Netherlands; Department of Urology, Erasmus University Medical Center, The Netherlands; Proteomics Center, Erasmus University Medical Center, Ee679a PO Box 2040, 3000CA Rotterdam, The Netherlands; Department of Biochemistry, Erasmus University Medical Center, Ee622 PO Box 2040, 3000CA Rotterdam, The Netherlands; Department of Pathology, Erasmus University Medical Center, The Netherlands; Department of Urology, Erasmus University Medical Center, The Netherlands

## Abstract

A major pharmacological strategy toward HIV cure aims to reverse latency in infected cells as a first step leading to their elimination. While the unbiased identification of molecular targets physically associated with the latent HIV-1 provirus would be highly valuable to unravel the molecular determinants of HIV-1 transcriptional repression and latency reversal, due to technical limitations, this has been challenging. Here we use a dCas9 targeted chromatin and histone enrichment strategy coupled to mass spectrometry (Catchet-MS) to probe the differential protein composition of the latent and activated HIV-1 5′LTR. Catchet-MS identified known and novel latent 5′LTR-associated host factors. Among these, IKZF1 is a novel HIV-1 transcriptional repressor, required for Polycomb Repressive Complex 2 recruitment to the LTR. We find the clinically advanced thalidomide analogue iberdomide, and the FDA approved analogues lenalidomide and pomalidomide, to be novel LRAs. We demonstrate that, by targeting IKZF1 for degradation, these compounds reverse HIV-1 latency in CD4+ T-cells isolated from virally suppressed people living with HIV-1 and that they are able to synergize with other known LRAs.

## INTRODUCTION

Combination antiretroviral therapy (cART) effectively blocks HIV replication and has significantly reduced AIDS-associated mortality. However, cART is not curative, has side effects, and, due to high costs, its global roll-out remains an ongoing challenge (UNAIDS fact sheet 2019). HIV persists because, subsequent to stable integration into the CD4+ T cell host genome, the provirus can remain in a nonproductive latent state, defined by the absence of HIV-1 gene expression. Because of this reservoir of latently HIV-1 infected cells, interruption of cART leads to rapid rebound of unrestricted viral replication, necessitating life-long treatment (1).

Strategies for HIV cure aim to eliminate, inactivate, or reduce the pool of latently infected cells such that the patient's immune system can control viral replication upon cessation of cART. As quiescent memory CD4+ T cells, which constitute the main cellular reservoir of latent HIV infected cells, have a long half-life (1), pharmacological approaches aim to speed up the decay rate of this infected reservoir. One such strategy is to induce viral expression in latently infected cells using latency reversal agents (LRAs) to increase viral protein expression, rendering the infected cells recognizable to the immune system or susceptible to viral cytopathic effects for elimination (2).

At the molecular level, the expression of the HIV-1 genome is determined by the activity of the HIV-1 promoter, which is controlled by the 5′LTR chromatin landscape (3), the engagement of sequence-specific host transcription factors (TFs) and associated cofactors (4,5), and the recruitment of RNA polymerase II (Pol II) and its efficient transcriptional elongation (6,7). Co-transcriptionally recruited host factors subsequently mediate post-transcriptional processing of the nascent HIV-1 viral RNA template for its efficient splicing, export, and translation (8–11). Host factors mediating these regulatory steps are thus the main targets of ongoing pharmacological strategies to reverse latency (12).

Several studies have shown that pharmacological reactivation of HIV-1 transcription is possible *in vivo* (13–16). However, due to the complex and heterogeneous nature of latency, the currently available LRAs are incapable of reactivating a significant portion of cells carrying a latent provirus (17) and have limited capacity to induce viral protein expression (18,19); thus they have failed to significantly impact the reservoir in patients (13–16,20). Additionally, LRAs targeting host molecular complexes with widespread regulatory functions are prone to non-specific pleiotropic effects. Besides, as viral clearance is a necessary step towards HIV-1 elimination (20,21), maintaining an intact cytotoxic compartment is of utmost importance in HIV-1 cure strategies. While being tolerable *in vivo*, LRAs can cause significant cytotoxicity to CD4+ and CD8+ T cells, induce global immune activation and may lead to impairment of T cell functionality (22). Therefore, there is an unmet need for novel LRAs that are able to significantly activate HIV-1 transcription *in vivo* while maintaining an intact immune compartment.

Identification of the repertoire of regulatory factors and cofactors involved in silencing the latent HIV 5′LTR is critical for a more complete understanding of the molecular mechanisms mediating latency and for the development of more specific and effective combinations of LRAs to deplete the HIV reservoir. However, due to technical limitations, unbiased proteomic analysis of host proteins associated with the latent or active HIV-1 promoter, in infected cells, has not been conducted.

Locus-specific strategies to identify *in vivo* chromatin-bound protein complexes at a single genomic location, such as the HIV-1 promoter, are largely based on a reverse chromatin immunoprecipitation (reverse-ChIP) protocol which relies on the targeting of an affinity bait to the chromatin region of interest, followed by purification of the bait and proteomic analysis of the proteins that co-purify with it (23,24). With the introduction of CRISPR-based nuclease-deficient Cas9 (dCas9) fusion constructs as a bait, reverse-ChIP strategies have become less labor-intensive and more versatile, without the need to introduce a potentially disruptive artificial consensus sequence required for targeting of the bait to the region of interest (25,26).

However, such techniques still represent an enormous challenge in protein biochemistry and proteomics; in each cell, the number of protein molecules bound at a specific regulatory region is exceedingly lower than that bound to the rest of the genome (27,28). Consequently these approaches require high amounts of input material, starting from at least a billion cells which despite several rounds of purification lead to a high signal to noise ratio and a low relative abundance of the single locus of interest with its bound protein (27–29). An additional caveat in locus-specific proteomics strategies is that a large fraction of the exogenously expressed bait is present diffusely in the cells and not exclusively bound to the locus of interest and therefore contributes to the nonspecific background signal. This contribution of non-localized bait molecules represents an enormous source of background signal that negatively affects sensitivity, and necessitates subtraction, through use of a robust set of negative controls from the final dataset and implementation of multiple replicates (25,27).

Here, we take an alternative two-tiered approach to reduce the background associated with non-target localized bait molecules causing the high signal to noise ratio of reverse-ChIP approaches. First, prior to semi-quantitative Mass Spectrometry identification, we added a histone purification step to our CRISPR-dCas9 based reverse-ChIP approach, to biochemically enrich for locus bound bait molecules and remove the unwanted background associated with non-target localized cytoplasmic and nucleoplasmic bait molecules; a purification pipeline we refer to as ‘dCas9 targeted chromatin and histone enrichment for mass spectrometry’ (Catchet-MS). Second, instead of using a non-targeted control, which has been reported to predominantly localize to the nucleolus (30,31), to eliminate potential background bound to the latent HIV-1 LTR, we also performed Catchet-MS in a re-activated HIV-1 infected isogenic cell line to identify the proteins bound to the same but re-activated locus; in our analysis we exclusively focused on proteins that were differentially bound to the latent versus the transcriptionally active HIV 5′LTR, a strategy which crucially allowed for stringent removal of potential nonspecific background without the need of additional negative controls.

Using Catchet-MS as a discovery tool we identify novel and previously reported proteins preferentially associated with the latent or active HIV-1 promoter that represent potential key regulators of HIV-1 gene expression. Among these regulators, we demonstrate that the transcription factor IKZF1 is a novel transcriptional repressor of the HIV-1 promoter, required for the sequential recruitment of the polycomb repressive complex 2 (PRC2) and critical for maintenance of a repressive LTR chromatin environment characterized by the presence of the H3K27me3 chromatin mark. Crucially, we demonstrate that the therapeutically advanced drug iberdomide (CC-220), and the FDA approved thalidomide analogues lenalidomide and pomalidomide, by targeting IKZF1 protein for CRL4^CRBN^ dependent degradation, lead to HIV-1 latency reversal in both *ex vivo* infected primary CD4 + T cells and cells isolated from aviremic cART-suppressed People Living With HIV (PLWHIV) and have a synergistic effect in latency reversal when combined with other FDA-approved LRAs. Our data demonstrate that modulation of IKZF1 protein expression with thalidomide derived drugs such as the FDA-approved lenalidomide and pomalidomide, represents a viable target for therapeutic intervention studies aiming to reverse latency towards HIV-1 cure.

## MATERIALS AND METHODS

### Cell lines

Latent HIV-1 infected Jurkat cells (clone J-Lat 11.1 and clone J-Lat A2) were cultured in RPMI-1640 media supplemented with 7% FBS and 100 μg/ml penicillin-streptomycin at 37°C in a humidified 95% air, 5% CO_2_ incubator. Primary CD4+ T cells were cultured in RPMI-1640 media supplemented with 7% FBS and 100 μg/ml penicillin-streptomycin at 37°C in a humidified 95% air–5% CO_2_ incubator. HEK 293T cells were cultured in DMEM supplemented with 10% FBS and 100 μg/ml penicillin-streptomycin at 37 ºC in a humidified 95% air 5% CO_2_ incubator and used exclusively for the production of lentiviral shRNA vectors.

### Establishment of J-lat 11.1 cells expressing the HA-V5-FLAG-dCas9 bait

**Figure 1.**
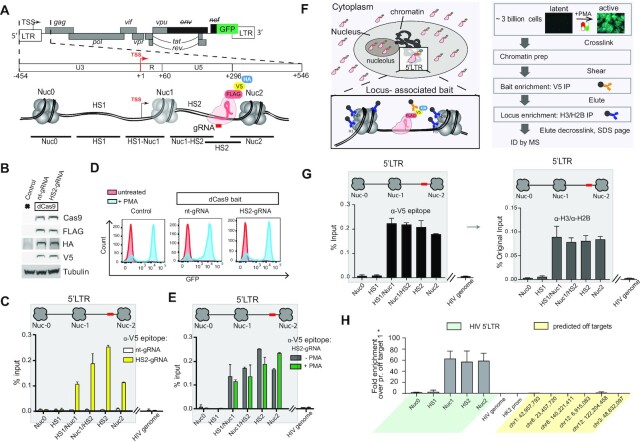
dCas9 targeted chromatin and histone enrichment for mass spectrometry (Catchet-MS), a method to isolate and identify locus-bound protein complexes *in vivo*. (**A**) Schematic representation of the genomic organization of the integrated HIV-1 provirus in J-Lat 11.1 cells, encoding GFP and containing a frameshift mutation in *env* and a partial deletion of *nef*. The 5′ LTR region is further segmented into the U3, R, and U5 regions. Three strictly positioned nucleosomes, Nuc-0, Nuc-1 and Nuc-2, delimit the nucleosome-free regions HS1 and HS2, hypersensitive to nuclease digestion, as indicated. The HS2 region, to which the multiple epitope-tagged HA-V5-FLAG-dCas9 bait is guided, is indicated. The amplicons used to scan the chromatin region in ChIP-qPCR are shown. (**B**) Western blot analysis indicates expression of multiple epitope-tagged HA-V5-FLAG-dCas9 bait in modified J-Lat 11.1 cells using antibodies specific for Cas9, V5, FLAG and HA as indicated. Parental J-Lat 11.1 cell lysate is used as a negative control and α-Tubulin is used as a loading control. (**C**) ChIP-qPCR analysis with anti V5 epitope affinity beads indicate specific enrichment of the HA-V5-FLAG-dCas9 bait over the guided HIV-1 LTR region. White bars represent data generated in cells expressing the dCas9 bait together with a non-targeting gRNA (nt-gRNA), yellow bars represent data generated in cells expressing the bait and the single guide RNA targeting the HS2 region of the HIV-1 5′LTR (HS2-gRNA). Data show a representative experiment, error bars represent the standard deviation (±SD) of two separate real-time PCR measurements. HIV-1 5′LTR sequences recovery is calculated as a percentage of the input. (**D**) Flow cytometry histograms show the distribution of GFP positive cells in unstimulated and PMA stimulated control J-Lat 11.1 cells, cells expressing the dCas9 bait and a non-targeting gRNA (nt-gRNA) and cells expressing the bait and the HS2 targeting gRNA (HS2-gRNA). (**E**) ChIP-qPCR analysis with anti V5 epitope affinity beads in latent (-PMA; grey bars) and PMA treated cells (+PMA; green bars) expressing the dCas9 bait and the HS2-gRNA. Data are the mean of 2 independent experiments (±SD). HIV-1 5′LTR sequences recovery is calculated as a percentage of the input. (**F**) Schematic representation of the dCas9 bait cellular localization (left panel) and the Catchet-MS workflow (right panel). Approximately 3 billion cells per condition are cross-linked with formaldehyde to stabilize the protein-protein and protein DNA interaction. Following a stringent chromatin enrichment protocol, the cross-linked chromatin is isolated and fragmented by ultrasound sonication. The dCas9 containing complexes are immunoprecipitated using anti V5 antibody conjugated affinity beads, eluted from the beads, and used as input material for a second round of purification with anti-histones (H3, H2B) antibody-conjugated beads, in order to remove the non-chromatin bound fraction of the HA-V5-FLAG-dCas9 bait complexes and to enrich for the locus associated bait complexes. Immunoprecipitated material is finally decrosslinked, resolved on an SDS-page and prepared for mass spectrometry analysis. (**G**) (Left panel) ChIP-qPCR analysis with anti V5 epitope affinity beads in chromatin fraction prepared from unstimulated cells indicates specific enrichment of HA-V5-FLAG-dCas9 over the HIV-1 5′LTR. Data show a representative experiment, error bars represent the standard deviation (SD) of two separate real-time PCR measurements, HIV-1 5′LTR levels are calculated as percentages of the input. The V5 affinity-purified chromatin (represented in top panel) was eluted and used as input for a sequential immunoprecipitation with a mix of histone H2B and H3 conjugated affinity beads and the isolated material was analyzed by qPCR (Right panel). ChIP-qPCR with anti H3/H2B conjugated affinity beads indicates 5′LTR enrichment of HA-V5-FLAG-dCas9 bait after sequential V5/histone affinity purification.Data show a representative experiment, error bars represent the standard deviation (SD) of two separate real-time PCR measurements, HIV-1 5′LTR levels in both the V5 and histone affinity purification are calculated as percentages of the same original input. (**H**) Average coverage profiles using ChIP sequencing reads mapped 500 bp upstream and downstream of the peak center at the 5′LTR region of the HIV genome (‘Targets’) to the respective coverage around the predicted off targets (‘OffTargets’). ‘Start’ denotes the starting base pair of the aforementioned 1 kb region around the peak centers and ‘End’ the ending base pair respectively.

In order to generate J-Lat 11.1 cells expressing the triple tagged (HA, V5, 3xFLAG) dCas9 construct, cells were then nucleofected with a modified version of the PX462 plasmid (AddGene) and selected for resistance to puromycin. pSpCas9n(BB)-2A-Puro (PX462) V2.0 was a gift from Feng Zhang (Addgene plasmid # 62987; http://n2t.net/addgene:62987; RRID:Addgene_62987). The original plasmid encodes for a Cas9 D10A nickase mutant, we modified the original sequence by introducing an H841A mutation that renders the Cas9 fully catalytically inactive (dCas9). Additionally, we introduced a triple FLAG epitope sequence (3xFLAG), a V5 epitope sequence and an HA epitope sequence at the N-terminal region of the dCas9. The plasmid contains a chicken β-actin promoter that drives the expression of the dCas9 bait and a U6 promoter that drives the expression of the gRNA scaffold. The sequence of the gRNA is 5′-GAAGCGCGCACGGCAAG-3′ and is located between nucleotides 708 and 724 on the HIV-1 5′ LTR, downstream the transcriptional start site (TSS) and in-between Nuc-1 and Nuc-2 (Figure 1A). Within the plasmid, the dCas9 open reading frame (ORF) sequence additionally contains an IRES sequence that is followed by a puromycin resistance cassette, allowing for puromycin selection of dCas9 expressing cells. The plasmid sequence information is available upon request. Forty-eight hours after nucleofection the cells were selected for a week with 1μg/ml puromycin and expanded in a polyclonal population. Unstimulated J-Lat 11.1 cells were used to model the latent, repressed, promoter while cells stimulated with 10 μm of phorbol 12 myristate 13-acetate (PMA) (Sigma), a potent PKC agonist, were used to model the transcriptionally active promoter.

### Plasmids nucleofection

The expression plasmids for the HA-V5-FLAG-dCas9 bait, the dCas9-VPR and shRNA vectors (scramble shRNA and shIKZF1) were delivered to J-Lat 11.1 cells by nucleofection using Amaxa Nucleofector (Lonza) as previously described (3) and the Cell Line Nucleofector Kit R (Lonza). Briefly, cells were split to 4 × 10^5^ cells/ml one day before nucleofection, 8 million cells were centrifuged at 600rcf for 5 min at room temperature, resuspended in 100 μl of solution R, and nucleofected with a total of 2 μg of plasmid, using program O28 and the Cell line nucleofector Kit R. Nucleofected cells were then resuspended in 500 μl of pre-warmed, serum-free antibiotic-free RPMI at 37 °C for 15 min and then plated in 5 ml of pre-warmed complete media. Seventy-two hours post-nucleofection cells nucleofected with the dCas9 VPR plasmid were analyzed with flow cytometry while cells nucleofected with the dCas9 construct for purification (HA, V5, FLAG) and shRNA constructswere selected for 10 days in puromycin 0.5 μg/ml and expanded as a polyclonal population.

### Bacterial strains

DH5α and Stable3 bacterial cells were used to propagate the plasmids used in the study.

### Western blotting

10 × 10^6^ cells were lysed with 100 μl cold lysis buffer containing 150 mM NaCl, 30 mM Tris (pH 7.5), 1 mM EDTA, 1% Triton X-100, 10% Glycerol, 0.5 mM DTT, protease inhibitor cocktail tablets (EDTA-free) (Roche) at 4°C for 30 min. Cell lysates were clarified by centrifugation (14 000 rpm for 30 min at 4°C), mixed with 4× sodium dodecyl sulfate (SDS)-loading buffer with 0.1 M DTT and boiled at 95°C for 5 min. Samples were run in a 10% SDS-polyacrylamide gel at 100 V. The proteins were then transferred to polyvinylidene difluoride (PVDF) membranes (Immobilion) and the membranes probed with primary antibodies (Supplementary Table S5) overnight at 4°C. Secondary antibodies were used accordingly to the species reactivity and incubated for 1 h at room temperature. Proteins were detected by chemical luminescence using the SuperSignal West Pico or SuperSignal West Pico Femto Chemiluminescent Substrate (Thermo Scientific) or by infrared imaging using IRDye^®^ secondary antibodies accordingly to the species reactivity and detected using LI-Cor Odyssey CLx.

For visualization of dCas9 protein enrichment during the ChIP-MS protocol, the protein complexes bound to the beads were decrosslinked,eluted and boiled at 95°C for 30 min in 4× sodium dodecyl sulfate (SDS)-loading buffer containing 0.1 M DTT. The proteins were then resolved in a 10% polyacrylamide SDS-PAGE gel and western blotting was performed using a mouse anti-FLAG antibody, a secondary anti-mouse HRP antibody, and imaged as above.

### Cytoplasmic and nuclear fractionation

Approximately 2 × 10^7^ J-lat 11.1 cells expressing the HA-V5-FLAG-dCas9 bait were subjected to cellular fractionation to separate the cytoplasmic and nuclear fraction. The cells were collected by centrifugation at 800rcf for 5 min at 4C and washed twice with PBS. The cell pellet was then transferred and resuspended into a pre-chilled 7 ml dounce homogenizer, where the cell membrane was broken using 10 strokes of a tight pestle. The dounced cells were then centrifuged for 5 min at 4C, 800rcf and the supernatant was retained as cytoplasmic fraction.

The nuclear pellets were then resuspended in 3 ml of a solution (S1) containing 0.25 M Sucrose and 10 mM MgCl_2_ and layered over a 3 ml cushion of a 0.35 M sucrose and 0.5 mM MgCl_2_ solution (S2) by slowly pipetting S1 on top S2. The cell pellets were centrifuged again for 10 min at 4C at 2500rcf and the remaining pellets retained as the nuclear fraction.

### Chromatin immunoprecipitation and HIV-1 5′LTR purification using Catchet-MS

In order to more specifically enrich for chromatin and reduce background from cellular contaminants and from the non-chromatin associated bait, we improved the stringency of our in-house ChIP protocol (3) by incorporating a few steps from the Chromatin enrichment for proteomics (CheP) protocol (32) during the preparation of our input material. subsequently we followed a traditional ChIP protocol in which we have enriched for the dCas9 bait and its associated complexes using an Anti-V5 Agarose Affinity Gel (Sigma). To further eliminate background originating from the purification of non-chromatin associated bait complexes we additionally added a histone enrichment step, downstream of the bait enrichment step, using histone H2B (Abcam) and H3 antibodies (Abcam) conjugated to Protein G Sepharose™ 4 Fast flow (GE Healthcare) and Protein A Sepharose™ 4 Fast flow (GE Healthcare) beads by dimethyl pimelimidate cross-linking as described in (33).

### Chromatin input preparation using CheIP

Approximately 3 billion cells per condition were collected, cross-linked and processed in batches of 400 million cells. After collection, by centrifugation at 800rcf for 5 min, each batch was washed two times in 40 ml PBS supplemented with 1 mM MgCl_2_ and 1 mM CaCl_2_, and cross-linked in 40 ml PBS with 1% formaldehyde (Formaldehyde methanol free 16%, Polysciences Inc.) at room temperature for 30 min with vertical rotation at 15 rpm. The reaction was quenched with the addition of 1 M glycine to a final concentration of 125 mM. Cross-linked cell pellets were then washed in 20 ml cold buffer B (0.25% Triton X-100, 1 mM EDTA, 0.5 mM EGTA, 20 mM HEPES pH 7.6) and 20 ml cold buffer C (0.15 M NaCl, 1 mM EDTA, 0.5 mM EGTA, 20 mM HEPES pH 7.6), resuspended in 2 ml ChIP incubation buffer (1% Triton X-100, 150 mM NaCl, 1 mM EDTA, pH 8.0, 0.5 mM EGTA, 20 mM HEPES pH 7.6, protease inhibitor cocktail tablets (EDTA-free, Roche) and then transferred to 15 ml polystyrene Falcon tubes compatible with sonication. In order to isolate and expose the cross-linked nuclei to the subsequent denaturation steps, the samples were sonicated with 15 ml probes at medium intensity for 10 min (30′ ON/30′ OFF intervals; Diagenode Bioruptor) and the cell nuclei were spun at 800rcf for 10 min. The pellets were then re-suspended in 2 ml ChIP incubation buffer containing 0.15% SDS and sonicated again for 10 minutes at medium intensity (30′ ON/30′ OFF intervals; Diagenode Bioruptor). The sonicated nuclei in suspension were aliquoted (500μls per tube) in 2 ml Eppendorf tubes and denatured for 5 min at room temperature with 350 μl 10% SDS and 1.2 ml 8 M urea buffer (10 mM Tris pH 7.4, 1mM EDTA and 8 M urea). The insoluble fraction of the nuclei, enriched for chromatin, was precipitated by centrifugation at 16200rcf at room temperature for 20 min. The precipitated pellets were resuspended and combined (4 into 1) in a single 2ml Falcon tube and denatured again for 5 min at room temperature with 500 μl of 4% SDS buffer (50 mM Tris pH 7.4, 10 mM EDTA, 4% SDS) and 8 M urea buffer and precipitated again by centrifugation at 16 200rcf at room temperature for 20 min.

The pellets were resuspended and washed by centrifugation at 16 200rcf at room temperature for 20 min in 2 ml 4% SDS buffer and resuspended in 2.5 ml ChIP incubation buffer containing 1% SDS. The content of each tube was transferred in a 15 ml polystyrene tube and sonicated (three tubes at the time) at a high intensity (30′ ON/30′ OFF intervals; Diagenode Bioruptor), using metal probes, to obtain chromatin fragments in a 200–500 bp size range. The different chromatin batches were then pulled together concentrated through 100kDa Centricon filters, to buffer exchange with 0.1% SDS ChIP incubation buffer and remove smaller proteins and fragments. The sonicated chromatin (a total of 50 ml per condition) was spun at max speed (20817 rcf) at 4°C for 30 min to remove cellular debris and diluted to 50 ml per condition. The chromatin was precleared overnight at 4°C with vertical rotation at 15 rpm (a total of 5 reactions per condition in 15 ml tubes) with 500 μl of a mix of Protein G and A Sepharose™ 4 Fast flow (GE Healthcare) beads and subsequently used for immunoprecipitation.

### HA-V5-FLAG-dCas9 bait enrichment

Per each condition, dCas9 containing complexes were isolated from the sonicated chromatin using 500 μl (a total of five reactions where 100 μl of affinity beads were used to purify 10ml of chromatin) of Anti-V5 Agarose Affinity Gel (Sigma) and then washed two times per each buffer (5minutes, 15rpm vertical rotation washes) with buffer 1 (0.1% SDS, 0.1% DOC, 1% Triton X-100, 150 mM NaCl, 1 mM EDTA pH 8.0, 0.5 mM EGTA, 20 mM HEPES pH 8.0), buffer 2 (500 mM NaCl: 0.1% SDS, 0.1% DOC, 1% Triton X-100, 500 mM NaCl, 1 mM EDTA pH 8.0, 0.5 mM EGTA, 20 mM HEPES pH 8.0), buffer 3 (0.25M LiCl, 0.5% DOC, 0.5% NP-40, 1 mM EDTA pH 8.0, 0.5 mM EGTA pH 8.0, 20 mM HEPES pH 8.0), buffer 4 (1 mM EDTA pH 8.0, 0.5 mM EGTA pH 8.0, 20 mM HEPES pH 8.0) to remove unspecific binding and eluted in a total volume of 1 ml elution buffer 1% SDS, 0.1 M NaHCO_3_ per each condition. At this stage, a 50 μl aliquot of the eluate was taken to be analyzed by qPCR as quality control for dCas9 enrichment over the chromatin input at the desired chromatin locus.

### Locus enrichment

The eluate was diluted 10 times in ChIP incubation buffer without SDS (1% Triton X-100, 150 mM NaCl, 1 mM EDTA, pH 8.0, 0.5 mM EGTA, 20 mM HEPES pH 7.6), protease inhibitor cocktail tablets (EDTA-free) (Roche) and immunoprecipitated again in a single reaction with 100 μl of a mix of Protein G and A SepharoseTM 4 Fast flow (GE Healthcare) beads pre-conjugated with histone H3 and histone H2B antibodies in order to specifically enrich for chromatin-associated dCas9 complexes and eliminate unspecific non-chromatin associated dCas9 complexes from the purification. The immunoprecipitated material, bound to the beads, was washed again 2 times per each buffer with buffer 1, buffer 2, buffer 3 and buffer 4 to remove unspecific binding and the protein complexes bound to the beads were finally eluted and decrosslinked by boiling the sample for 30 min in 100 μl of 4× sodium dodecyl sulfate (SDS)-loading buffer, containing 0.1M DTT. Beads were re-eluted and processed for mass spectrometry analysis as described above.

### Mass spectrometry

Proteins were resolved in a 15% polyacrylamide SDS-PAGE gel, visualized by Colloidal Blue Staining Kit (ThermoFisher) and prepared for nanoflow LC MS/MS analysis. SDS-PAGE gel lanes were cut into 1-mm slices and proteins were subjected to in-gel reduction with dithiothreitol, alkylation with iodoacetamide and digested with trypsin (TPCK Trypsin, ThermoScientific, Rockford, IL, USA), as described previously (34). Peptides were extracted from the gel blocks in 30% acetonitrile (Biosolve) and 0.5% formic acid (Biosolve) and dried in a SpeedVac Vacuum Concentrator (ThermoFisher). Samples were analyzed by LC MS/MS on a 20 cm × 75 μm C18 column (BEH C18, 130 Å, 3.5 μm, Waters) after trapping on a nanoAcquity UPLC Symmetry C18 trapping column (Waters, 100 Å, 5 μm, 180 μm × 20 mm) on an EASY-nLC 1000 coupled to a Fusion Tribrid Orbitrap mass spectrometer (ThermoFisher Scientific), essentially as described in (35). Data analysis was performed with the Mascot software suite (Daemon version 2.3.2 and Distiller version 2.3.02, Matrix Science) within the Proteome Discoverer (version 2.2, ThermoFisher Scientific) framework. Spectra were searched against a Uniprot database (version April 2017, taxonomy *Homo sapiens*) concatenated with a fasta database containing amino acid sequences of the triple tagged dCas9 (HA, V5, 3xFLAG) construct. Protein Mascot scores and peptide numbers were taken directly from the Mascot output and reported. The same procedure was used to analyze input samples for the protocol characterization (Supplementary Figure S2). Heat maps were generated using MORPHEUS (http://software.broadintistute.org/morpheus/index.html). Samples F29 and F30, corresponding to re-eluted histone pulldown samples, were excluded from further analysis (Supplementary Table S4).

### Chromatin quality control

100μl aliquots of chromatin were taken per each batch of the purification protocol, resuspended in 500 μl of ChIP elution buffer and, after addition of 24 μl 5 M NaCl (200 mM final concentration), decrosslinked overnight at 65°C on a heat block. DNA isolation was then performed using phenol–chloroform isoamyl isolation method and ethanol precipitation in the presence of glycogen as a carrier. The DNA pellet was resuspended in 100 μl of nuclease-free water. 5 μl of DNA was mixed with sample 4× sample buffer and run on a 1% agarose DNA electrophoreses gel to check for fragmentation between 200 and 500 bp.

### ChIP-qPCR

Real-time qPCR was used to detect the specific enrichment of the 5′-LTR locus at a DNA level after the ChIP procedure. Reactions of 10 μl were performed with the GoTaq qPCR Master Mix kit (Promega) in a CFX Connect Real-Time PCR thermocycler (Biorad). Relative quantitation was calculated with the 2-ΔCt method where relative enrichment over the adjusted DNA input was used for data representation.

To evaluate IKZF1, CBX8 and SUZ12 binding to the HIV-1 promoter and histone mark profiles a smaller scale ChIP experiment (50 million cells) with identical preparation of the chromatin input as for the mass spectrometry experiment was performed. For the immunoprecipitation, protein G-beads or A-beads were used in combination with the anti-IKZF1 antibody (Cell Signal Technology), anti-SUZ12 (Abcam), anti CBX8 (homemade) and histone antibodies: Histone H3 (Abcam), histone H3K27me3 (Millipore), H3K4me3 (Millipore), H3K9me3 (Abcam). DNA was PCI extracted and used to evaluate the enrichment at the HIV-1 5′LTR by the same primer sets summarized in the key resources table. Antibodies used are summarized in the key resource table.

### ChIP-sequencing data analysis

In order to align the short reads produced by the ChIP-Seq procedure and also capture reads mapped to the HIV genome, we constructed a custom version of the human genome (UCSC version hg38) where we attached the HIV genome as an additional chromosome. We used the HIV strain K03455.1 (https://www.ncbi.nlm.nih.gov/nuccore/K03455). Subsequently, the resulting genome was indexed and reads were aligned with bwa 0.7.17 (1) and the bwa mem algorithm. In order to compensate for differences in library sizes between ChIP and Input DNA sequencing samples, the total number of reads of each sample was equalized by uniformly downsampling reads relatively to the sample with the lower number of reads. ChIP peaks were called with MACS2 (2) using default parameters. The peaks returned by MACS2 were further filtered by (i) imposing an additional MACS FDR threshold of 0.1%, (ii) excluding peaks demonstrating a fold enrichment <3 in log_2_ scale (where fold enrichment is the ratio of normalized reads under a peak area in the ChIP sample to the respective number of normalized reads in the Input DNA sample). Finally, in order to visualize the ChIP-Seq signals, we constructed a custom UCSC track hub based on the combined hg38-HIV genomes using instructions and tools provided by UCSC (36,37).

### Production of shRNA lentiviral vectors

Lentiviral constructs containing the desired shRNA sequence were amplified from bacterial glycerol stocks obtained in house from the Erasmus Center for Biomics and part of the MISSION^®^ shRNA library (Sigma). Briefly, 5.0 × 10^6^ HEK293T cells were plated in a 10 cm dish and transfected with 12.5 μg of plasmids mix. 4.5 μg of pCMVΔR8.9 (envelope), 2 μg of pCMV-VSV-G (packaging) and 6 ug of shRNA vector were mixed in 500 μl serum-free DMEM and combined with 500 μl DMEM containing 125 μl of 10 mM polyethyleneimine (PEI, Sigma). The resulting 1 mL mixture was applied to HEK293T cells after 15 min incubation at room temperature. The transfection mix was removed after 12 h and replaced with fresh RPMI medium. Virus-containing medium was harvested twice and replaced with fresh medium at 36, 48 , 60 and 72 h post-transfection. After each harvest, the collected medium was filtered through a cellulose acetate membrane (0.45 μm pore) and used directly for shRNA infections or stored at −80°C for subsequent use. Lentivaral vectors used in the study are summarized in Supplementary Table S5.

### Flow cytometry of J-lat 11.1

GFP expression in the J-Lat cell lines was analyzed by Flow Cytometry. The live-cell population was defined by using the forward scatter area versus side scatter area profiles (FSC-A, SSC-A). Single cells were then gated by using forward scatter height (FSC-H) versus forward scatter width (FSC-W) and side scatter height (SSC-H) versus side scatter width (SSC-W). GFP intensity to differentiate between GFP-positive and negative cells. Between 2–4 × 10^5^ events were collected per sample on an LSR Fortessa (BD Biosciences) and analyzed using FlowJo software (version 9.7.4, Tree Star).

### Fluorescence microscopy

Fluorescence microscopy and bright field pictures of J-Lat 11.1 cells in the absence and presence of PMA were acquired using an Olympus IX70 Fluorescence Phase Contrast inverted Microscope.

### Total RNA isolation and quantitative real-time PCR (RT-qPCR)

Total RNA was isolated from the cells using Trizol (Thermo Fisher) and the Total RNA Zol out kit (A&A Biotechnology) and residual genomic DNA digested with DNAseI (Life technologies). cDNA synthesis reactions were performed with Superscript II Reverse Transcriptase (Life Technologies) kit following with random priming and manufacturer's instructions. RT-qPCR reactions were conducted CFX Connect Real-Time PCR Detection System thermocycler (BioRad) using GoTaq qPCR Master Mix (Promega) following manufacturer protocol. Amplification was performed on the using following thermal program starting with 3 min at 95°C, followed by 40 cycles of 95°C for 10 s and 60°C for 30 s. The specificity of the RT-qPCR products was assessed by melting curve analysis. Primers used for real-time PCR are listed in Supplementary Table S5. Expression data was calculated using 2-ΔΔCt method (38). Cyclophilin A (CyPA), GAPDH and β2 microglobulin (B2M) were used as housekeeping genes for the analysis.

### Isolation and *ex vivo* infection of primary CD4+ T cells

Peripheral blood mononuclear cells (PBMCs) were isolated by Ficoll density gradient sedimentation of buffy coats from healthy donors (GE Healthcare). Total CD4+ T cells were separated by negative selection using EasySep Human CD4+ T Cell Enrichment Cocktail (StemCell Technologies). Primary CD4+ T cells were plated at a concentration of 1.5 × 10^6^ cells/ml left overnight for recovery. HIV-1 latency *ex vivo* model was generated by spinoculation according to Lassen and Greene method as described elsewhere (39). Pseudotyped viral particles were obtained from co-transfecting HEK 293T cells with HXB2 Env and pNL4.3.Luc.R-E-plasmids using PEI. Supernatants were collected at 48 and 72 h post-transfection, filtered with a 0.45 μM filter and stored at −80°C. Expression vectors HXB2 Env and pNL4.3.Luc.R-E- were donated by Dr Nathaniel Landau and Drs Kathleen Page and Dan Littman, respectively. Antiretroviral drugs Saquinavir Mesylate and Raltegravir were kindly provided by the Centre for AIDS reagents, NIBSC.

### Flow cytometry for T cells activation and toxicity assay

Primary CD4+ T cells isolated from buffy coats of healthy volunteers were treated with 10 μM of iberdomide, 500 nM of JQ1, or both compounds, with PMA/ionomycin used as a positive control. The cells were examined by flow-cytometry at 24 and 48 h, live cells were gated using forward scatter area versus side scatter area profiles (FSC-A, SSC-A). Cells were then stained for expression of Annexin V to examine the percentage of cells undergoing apoptosis and with the surface receptor CD69 to measure T cell activation. For Annexin V and CD69 staining, 10^6^ cells were washed with PBS supplemented with 3% FBS and 2.5 mM CaCl_2_ followed by staining with Annexin V-PE (Becton and Dickinson), CD69-FITC (eBIOSCIENCE) for 20 min at 4C in the presence of 2.5 mM CaCl_2_. Cells were then washed with PBS/FBS/CaCl_2_ and analyzed by flow cytometry. Between 2–4 × 10^5^ events were collected per sample within 3 h after staining on an LSRFortessa (BD Biosciences, 4 lasers, 18 parameters) and analyzed using FlowJo software (version 9.7.4, Tree Star).

### T cell proliferation and functionality assay

To analyze the effect of the LRA on CD8+ and CD4+ T cells, proliferation and cytokine expression were analyzed by flow cytometry. Primary CD8+ and CD4+ T cells were isolated from buffy coats from healthy donors by Ficoll gradient (GE Healthcare) followed by negative selection with RosetteSep Human CD8+ T Cell Enrichment Cocktail or RosetteSep Human CD4+ T Cell Enrichment Cocktail (StemCell Technologies) respectively. Isolated cells were left overnight for recovery. To analyze T cell proliferation capacity, 1 million CD8+ or CD4+ T cells were stained with 0.07 μM CellTrace Far Red Cell Proliferation dye (ThermoFisher Scientific) following manufacturer's instructions. Cells were then cultivated for 72 h with either unstimulated or stimulated conditions in the presence of the LRA, and analyzed by flow cytometry. Stimulation of T cells was performed using Anti-CD3/CD28 coated Dynabeads (ThermoFisher Scientific) following manufacturer's protocol. Proliferation capacity was determined by a decrease in proliferation dye intensity in daughter cells upon cell division. To analyze T cell functionality by means of cytokine expression 1 million CD8+ or CD4+ T cells were left untreated or were treated with the LRA for 18 h. Cells were then left unstimulated or stimulated with 50 ng/ml PMA and 1μM Ionomycin for 7 h in the presence of a protein transport inhibitor (BD GolgiPlug™, BD Biosciences). To stain for intracellular cytokines cells were washed with PBS supplemented with 3% FBS followed by a fixation and permeabilization step with the FIX & PERM Kit (Invitrogen) following manufacturer's protocol and incubated with 1:25 BV510 anti-IL2 (563265, BD Biosciences) and PE-Cy7 anti-IFNg (27-7319-41, eBioscience) in permeabilization buffer for 45 min at 4°C. Stained cells were washed with PBS supplemented with 3% FBS and analyzed by flow cytometry.

### HIV-1 latency reversal in primary CD4+ T cells isolated from aviremic patients

Primary CD4+ T cells from five aviremic patients (maintained viremia below 50 copies/ml for at least 2 years) were isolated as described previously (40). Two million CD4+ T cells were plated at the cell density of 10^6^/ml and treated as indicated. After 16 h, cells were lysed in TRI reagent and RNA was isolated with Total RNA Zol-out kit (A&A Biotechnology), cDNA synthesis was performed as described above. Absolute quantification of cell-associated HIV-1 gag RNA was performed in a nested PCR protocol modified from Pasternak *et al.* (41). Briefly, first round of amplification was performed in a final volume of 25 μl using 4 μl of cDNA, 2.5 μl of 10× PCR buffer (Life Technologies), 1 μl of 50 mM MgCl_2_ (Life Technologies), 1 μl of 10 mM dNTPs (Life Technologies), 0.075 μl of 100 μM Gag Forward primer, 0.075 μl of 100 μM SK437 Reverse primer, and 0.2 μl Platinum Taq polymerase (Life Technologies). The second round of amplification was performed in a final volume of 25 μl using 2 μl of pre-amplified cDNA, 2.5 μl of 10× PCR buffer (Life Technologies), 1 μl of 50 mM MgCl_2_ (Life Technologies), 1 μl of 10 mM dNTPs (Life Technologies), 0.05 μl of 100 μM Gag Forward primer, 0.05 μl of 100 μM Gag Reverse primer, 0.075 μl of 50 μM Gag Probe and 0.2 μl Platinum Taq polymerase. The absolute number of gag copies in the PCR was calculated using a standard curve ranging from 8 to 512 copies of a plasmid containing the full-length HIV-1 genome (pNL4.3.Luc.R-E-). The amount of HIV-1 cellular associated RNA was expressed as number of copies/μg of input RNA in reverse transcription.

The study was conducted according to the ethical principles of the Declaration of Helsinki. All patients involved in the study gave their written informed consent. The study protocol and any amendment were approved by The Netherlands Medical Ethics Committee (MEC-2012–583).

### FISH-Flow analysis

Cells were collected, fixed, permeabilised and subjected to the PrimeFlow RNA assay (Thermo Fisher Scientific) following the manufacturer's instructions and as described in (21). Primary CD4+ T cells were first stained in Fixable Viability dye 780 (Thermo Fisher Scientific) for 20 min at room temperature (1:1000 in dPBS). HIV unspliced mRNA was then labelled with a set of 40 probe pairs against the GagPol region of the vRNA (catalogue number GagPol HIV-1 VF10-10884, Thermo Fisher Scientific) diluted 1:5 in diluent provided in the kit and hybridized to the target mRNA for 2 h at 40°C. Samples were washed to remove excess probes and stored overnight in the presence of RNAsin. Signal amplification was performed by sequential 1.5 h, 40°C incubations with the pre-amplification and amplification mix. Amplified mRNA was labelled with fluorescently-tagged probes for 1 h at 40°C. Samples were acquired on a BD LSR Fortessa Analyzer and gates were set using the uninfected CD4+ T cells. The analysis was performed using the FlowJo V10 software (Treestar).

### Statistical analysis

Statistical analysis was conducted as indicated in the figure legends using Prism version 8.3.0 (GraphPad software). Continuous data from two groups were compared using a *t*-test. An unpaired *t*-test was chosen when the data were independent. Conversely, a paired *t*-test was used when the data were dependent (i.e. different conditions were compared from data that were obtained from the same donor at the same time point). A one-way ANOVA was performed when comparing the effect of at least three different groups. Tukey's post hoc test for multiple comparisons was then used to determine which groups were different.

## RESULTS

### Catchet-MS, a target discovery strategy to identify protein complexes differentially bound at the latent and activated HIV-1 LTR *in vivo*

We established a CRISPR/dCas9-based reverse-ChIP proteomics strategy that incorporates a sequential purification step targeting histones which we refer to as ‘dCas9 targeted chromatin and histone enrichment for mass spectrometry’’ (Catchet-MS) to identify a comprehensive repertoire of protein complexes differentially associated *in vivo* with the latent and active HIV promoter (Figure 1). As a cellular model system for latency we used J-Lat 11.1, a Jurkat derived T cell line, which harbors a single latent copy of the integrated full-length HIV-1 proviral genome, defective in *env*, and in which GFP replaces the *nef* coding sequence as a reporter for viral activation (42) (Figure 1A). J-Lat 11.1 cells were first modified to stably express a multiple epitope (V5, HA, FLAG)-tagged dCas9 bait, and an 18 nucleotide single guide-RNA, targeting the hypersensitive site 2 (HS2) region of the HIV-1 promoter, downstream of the Transcription Start Site (TSS) (Figure 1A). We chose to target our dCas9 bait to the HS2 region downstream of Nuc-1, as recent evidence pointed to transcription elongation and RNA processing as critical steps involved in HIV-1 latency *in vivo* (43). As a negative control, we generated cells expressing HA-V5-FLAG-dCas9 and a non-targeting control gRNA (nt-gRNA). Expression of the tagged dCas9 bait construct was confirmed by Western blotting, using antibodies specifically recognizing dCas9 and the construct tags HA, V5, and FLAG (Figure 1B). We validated gRNA (HS2-gRNA) dependent HIV-1 promoter targeting in a functional assay by transfecting J-Lat 11.1 cells with a dCas9-VP64-p65-Rta (dCas9-VPR) fusion construct (Supplementary Figure S1A), and by examining the specific binding of the HA-V5-FLAG-dCas9 bait at the HS2 region of the HIV-1 promoter in latent J-Lat 11.1 cells by V5 epitope ChIP-qPCR (Figure 1C).

Binding of the HA-V5-FLAG-dCas9 bait did not interfere functionally with viral reactivation as demonstrated by Flow cytometry and fluorescence microscopy of GFP expression in the HA-V5-FLAG-dCas9 expressing J-Lat 11.1 cells both before and after PMA stimulation (Figure 1D and supplementary Figure S1B). In addition, HA-V5-FLAG-dCas9 binding was not affected by the transcriptional state of the 5′LTR as demonstrated by ChIP-qPCR in latent and PMA stimulated cells (Figure 1E). Therefore we concluded that the dCas9 bait is properly targeted to the 5′HIV LTR and does not interfere with the maintenance of latency or capacity for reactivation. From this, we inferred that binding of 5′LTR associated transcription regulatory factors bound in the repressed or activated states is unaltered by dCas9 targeting.

We determined the optimal HA-V5-FLAG-dCas9 bait IP enrichment strategy by examining specific enrichment of the locus using antibodies directed against the HA, V5 and FLAG epitopes in ChIP-qPCR experiments (Supplementary Figure S1C, D). While antibodies directed against each tag resulted in efficient and specific enrichment of the targeted HIV-1 DNA region, we chose to use anti V5 affinity beads targeting the V5 epitope in the large-scale locus enrichment strategy as it provided both good purification yield as well as the highest signal to noise ratio (Supplementary Figure S1D).

The large number of non-localized HA-V5-FLAG-dCas9 bait molecules presents a huge source of background in our single locus chromatin proteomics reverse-ChIP approach as they are also captured in the purification pipeline, thus interfering with reliable identification of the locus associated bait interactome. It has been demonstrated that Cas9 molecules, when non-efficiently guided are diffusely present in the nucleus and tend to accumulate in the RNA-rich nucleolar compartment (30,31). In our experimental system, cell fractionation followed by western blotting revealed that a large fraction of the HA-V5-FLAG-dCas9 bait molecules is present in the cytoplasmic fraction, despite the presence of a nuclear localization sequence (NLS) within the bait construct (Figure 1F, left panel, Supplementary Figure S2A). To obtain a purer chromatin fraction, we included 10 cycles of low-efficiency sonication in the presence of strong denaturing buffers after nuclear isolation into our in-house ChIP protocol similar to what was described in the CheP protocol (32). Mass spectrometry analysis of individual steps of our purification pipeline demonstrated progressive removal of membrane, cytoplasmic and organelle associated proteins as well as, in part, non-chromatin bound nuclear proteins by CheP, resulting in the enrichment of chromatin associated proteins within the insoluble nuclear material (Supplementary Figure S2B, C).

To improve the specificity of the purification, we introduced a histone enrichment step into our purification pipeline after the V5 epitope-based purification of the HA-V5-FLAG-dCas9 bait-containing cross-linked complexes (Figure 1F), thus eliminating the need for large amounts of histone antibodies. As shown in Figure 1G (left panel), ChIP, using anti V5 affinity beads efficiently enriched for the HIV-1 promoter region targeted by the HA-V5-FLAG-dCas9. Western blotting of the decrosslinked ChIP eluate confirmed efficient bait recovery by immunoprecipitation (Supplementary Figure S2D). The subsequential immunoprecipitation, using anti H3- anti H2B antibodies conjugated beads, resulted in a loss of (non-chromatin bound) HA-V5-FLAG-dCas9 bait (Supplementary Figure S2D), while retaining the HIV-1 target locus of interest (Figure 1G) (right panel).

The specificity of the guided dCas9 is a critical point for locus purification. We, therefore, performed ChIP-sequencing experiments to comprehensively investigate the locus specificity of our bait, which is guided and predicted to specifically bind to the integrated HIV-1 5′LTR. ChIP-sequencing was performed using the sequentially purified chromatin, first immunoprecipitated using V5 affinity beads, eluted and re-immunoprecipitated using Histone H3–H2B affinity purification. Both in ChIP-seq (Supplementary Figure S2E, F) and by ChIP qPCR (Figure 1H) we observe a strong enrichment on the HIV-1 5′ LTR while enrichment at human chromosomes, aside from centromeric artifacts common to ChIP-seq and a few minor off-target peaks (Supplementary Table S1), is low. Next, we specifically monitored the recovery of the top potential dCas9 off-target binding sites as predicted for the HS2 gRNA sequence (5′-GAAGCGCGCACGGCAAG-3′) by the online tool http://www.rgenome.net/cas-offinder/. Zooming in on those predicted off-target regions in the ChIP-seq data (Supplementary Figure S2E, F) and by ChIP qPCR (Figure 1H) demonstrate the absence of any dCas9 binding to these chromosomal loci. In summary our results demonstrate that Catchet-MS enables highly specific enrichment of the targeted HIV-1 5′LTR bound proteome while cellular proteins are depleted, indicating its suitability as a discovery tool to identify HIV-1 5′LTR associated host factors.

### Cachet-MS identifies known and novel host factors associated with the latent and active HIV-1 promoter

**Figure 2.**
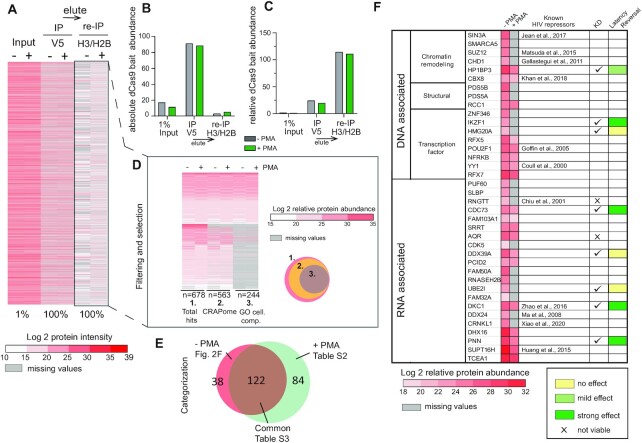
Cachet-MS identifies known and novel host factors associated with the latent and active HIV-1 promoter. (**A**) Heatmap displays the protein content at each step of the Catchet-MS purification pipeline. The colors represents the Log2 transformation of the protein intensities scores. Values corresponding to the V5 based immunopurification are adjusted to 100% as only a fraction (1:40) of the material that went into the second, histone based (H2B/H3) immunopurification, was analyzed by mass spectrometry. For the V5/histone experiment, 100% of the material was subjected to mass spectrometry. Missing values are represented by grey lines. The heatmap represents data from one Catchet-MS experiment, input material for the experiment corresponds to chromatin generated from a starting material of 3 billion cells per condition. (**B**) Bar plots showing the absolute HA-V5-FLAG-dCas9 bait abundance at each step of the purification pipeline. The absolute abundance was calculated based on protein/peptide spectral intensity values and adjusted to the fraction of material analyzed by mass spectrometry. The values are displayed in arbitrary units. (**C**) Bar plots showing the relative HA-V5-FLAG-dCas9 bait abundance at each step of the purification pipeline. The relative abundance was calculated based on protein/peptide spectral intensity values and normalized to the total protein content. The values are displayed in arbitrary units. (**D**) Filtering criteria applied to Catchet-MS data. Hits are filtered by comparison with the Crapome repository for contaminants of affinity purification experiments. as well as through GO categorization to select hits present in the cell nucleus and classified to be RNA bound, DNA bound or alternatively bound to histones, transcription factors or associated to chromatin. Colors represent the Log2 transformation of the protein relative abundance. The protein relative abundance was calculated based on protein/peptide spectral intensity values and normalized to the total protein content. Missing values are represented by grey lines. (**E**) Venn diagram graphically summarizing the overlap between the hits identified to be exclusively, or more abundantly, associated with the unstimulated state (−PMA) and the hits associated with the activated state (+PMA). (**F**) Selection and functional classification of hits associated with the unstimulated state and considered for a potential role in the maintenance of HIV-1 latency. The first columns of the table summarizes in a heatmap the log_2_ trasfomation of the protein relative abundance values in the unstimulated (–PMA) sample and the activated (+PMA) sample. Missing values are represented by grey lines. Candidates previously reported to restrict HIV-1 expression are referenced in the third column. Checkmarks indicate the hits functionally validated by shRNA mediated depletion experiments (Supplementary Figure S3). Colored boxes (yellow to dark green) summarize the effect of the target depletion on HIV- 1 expression and refers to the effects summarized in Supplementary Figure S3.

We scaled up the Catchet-MS workflow (Figure 1F), to ∼3 billion cells as starting material in the unstimulated and PMA stimulated conditions to model J-Lat 11.1 HIV-1 latency and activation. Figure 2A presents a heatmap of the mass spectrometry analysis performed at different steps of the Catchet-MS purification pipeline. The heatmap of 1% of the input chromatin material, prepared using the optimized CheP protocol in both latent and activated states shows high sample complexity, characterized by a large number of high protein intensity values (Figure 2A). The input samples are enriched for nuclear and chromatin-associated proteins but also contain numerous cellular contaminants, resulting from carryover precipitation of insoluble, non-chromatin associated complexes, while as expected, the abundance of the HA-V5-FLAG-dCas9 bait is low (Figure 2B and C). The anti-V5 affinity purification step results in a marked reduction in sample complexity (Figure 2A) concomitant with high enrichment of the HA-V5-FLAG-dCas9 bait (Figure 2B and C). Finally, re-immunoprecipitation using anti-Histone H2B and H3 conjugated affinity beads results in a clear difference in protein abundances between the unstimulated and PMA stimulated samples (Figure 2A). As expected, given the known accumulation of non-locus bound Cas9 in the RNA-rich nucleolar compartment (30,31), V5 affinity purification resulted in an increase in the number of nucleolar proteins detected, followed by their subsequent loss following the succeeding histone affinity purification step (Supplementary Figure S2G). Relative abundance of histone proteins is reduced during the V5 enrichment step while, as expected, they are retained after the histone H3/H2B enrichment step (Supplementary Figure S2H).

Analysis of the MS data from the final locus enrichment step resulted in a list of 669 proteins, identified with high confidence, Mascot score >100, bound to either the latent or active HIV-1 5′LTR. We then applied stringent and unbiased selection criteria, Illustrated in Figure 2D, to remove potential contaminants. First, we filtered out frequent contaminants using the Crapome database (https://www.crapome.org/), second we applied a GO cellular compartment and function analysis (GO-terms nucleus, chromatin-associated, histones bound, bound to transcription factors, DNA associated, or RNA associated) to include only nuclear proteins in our further analysis. Finally and crucially, to further eliminate non-specific contaminants, we determined the differential proteome of latent versus activated HIV-1 LTR bound factors and excluded proteins common to both conditions, which include potential non-specific background proteins from further analysis. While this approach may also result in elimination of potentially functionally relevant factors bound to both transcriptional states of the promoter, it has the advantage that, in contrast to a non-targeted control which only captures general nucleoplasmic contaminants, it allows for a stringent removal of a-specific background proteins preferentially sticking to HIV-1 5′LTR chromatin. Applying these criteria, we compiled a list (n = 38) of proteins predominantly associated with the HIV-1 promoter in the latent state (Figure 2E and F), and a list (n = 84) of factors predominantly associated with the activated HIV-1 promoter following treatment with PMA (Figure 2E and Supplementary Table S2), while (n = 122) common proteins, including the dCas9 bait and potential contaminants, were found associated with the HIV-1 5′LTR under both conditions (Figure 2E and Supplementary Table S3)

### Functional validation of proteins identified to associate with the latent promoter

In search of putative molecular targets for therapeutic inhibition and HIV-1 latency reversal, we exclusively selected the protein hits that were distinctly associated with the latent HIV-1 LTR, *in vivo*, comprising the 38 factors presented in the table in Figure 2F. Of these latent HIV-1 LTR-associated candidates, a significant number (*n* = 11) were previously reported to restrict viral expression (Figure 2F referenced in the table) and thus serve as positive controls that validate the quality of the experimental approach, providing confidence towards the veracity of the candidate list. We evaluated the effect of shRNA-mediated knockdown of a selection of the novel factors we identified, on the HIV-1 5′LTR-driven GFP reporter by flow cytometry and on the expression of HIV-1 genes Gag, pol, and Tat by RT-PCR in J-Lat 11.1 cells (Figure 2F; Supplementary Figure S3). Depletion of a number of the selected candidates (*n* = 5/10, namely HP1BP3, IKZF1, CDC73, DKC1, and PNN), resulted in significant (*P* < 0.05) upregulation of HIV-1 genes and an increase in the percentage of GFP positive cells (green boxes, Figure 2F and Supplementary Figure S3), while for a small number of factors tested (*N* = 3/10), we did not observe a significant change in HIV-1 expression (yellow boxes, Figure 2F) and the remaining factors were found to be essential for cell viability (x mark, Figure 2F). Our data indicate that the selected *n* = 5 candidates are required for repression of HIV-1 expression in J-Lat 11.1 cells.

### IKZF1 binds to the latent HIV-1 promoter and is repressive to HIV-1 transcription

**Figure 3.**
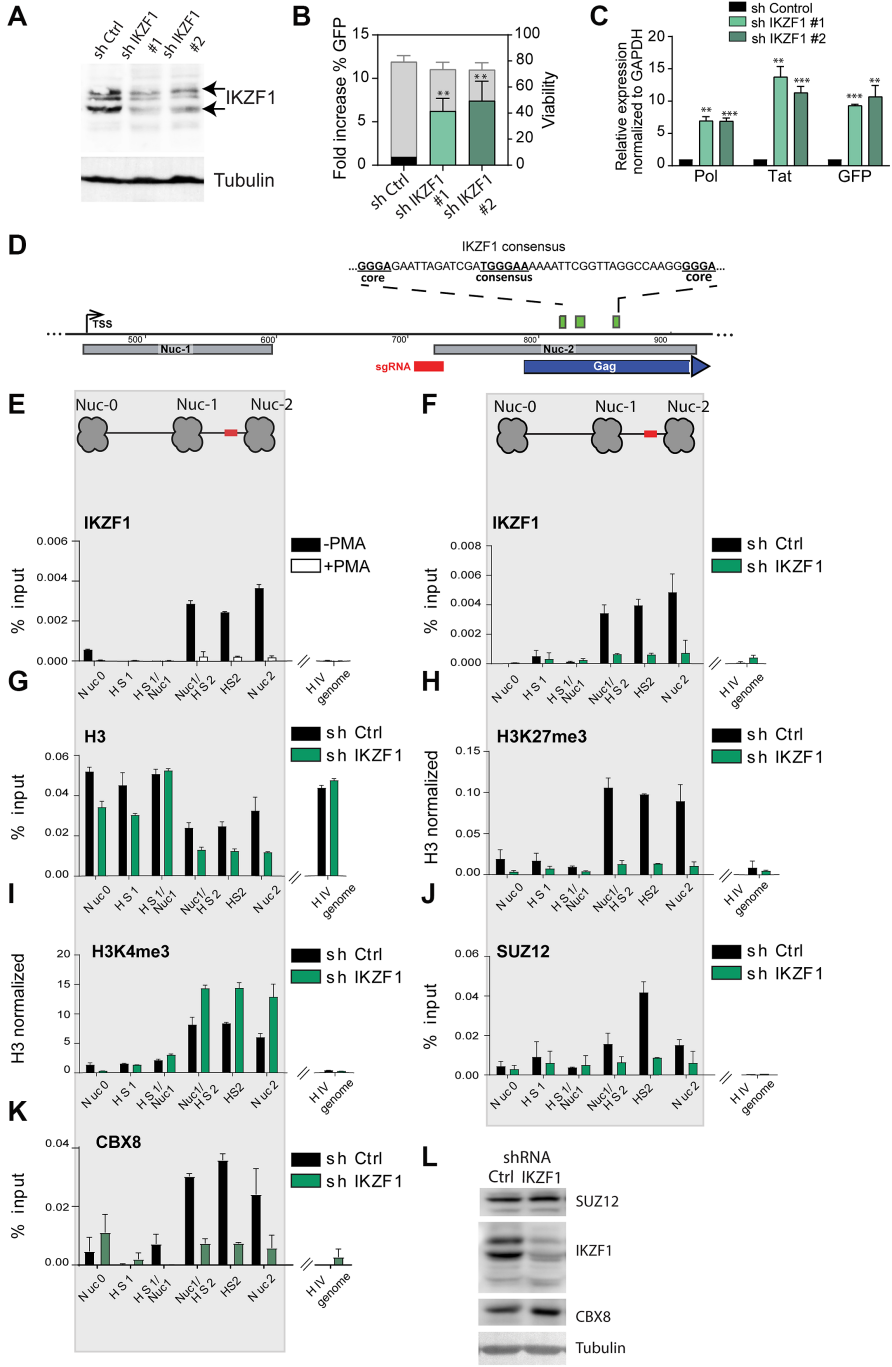
IKZF1, required for maintenance of HIV-1 latency, binds downstream of the latent HIV-1 5′LTR to promote PRC2/PRC1 recruitment and establish a repressive chromatin environment. (**A**) Western blotting for IKZF1 in J-lat 11 transduced with scramble shRNA (sh Control) and shIKZF1 #1 and #2 iondicates depletion of IKZF1. α-Tubulin is used as a loading control. (**B**) Bar plot showing the fold increase in % GFP positive cells (left y axes) measured by flow cytometry analysis, following IKZF1 depletion by lentiviral transduction in J-Lat 11.1 with two different shRNA constructs (#1 and #2). Data are the mean of three independent experiments (±SD). The right y axis represents the percentage of live cells. (**C**) qRT-PCR analysis measuring expression of HIV-1 genes (pol, GFP, tat) in J-lat 11 transduced with scramble shRNA (sh Control) and sh IKZF1 #1 and #2. Data, normalized to GAPDH are represented as fold enrichment over sh Control and are the mean of three independent experiments (±SEM). Statistical significance was calculated using a unpaired *t* test, *P < 0.05; **P < 0.01; ***P < 0.001. (**D**) Putative IKZF1 binding site (835–840) downstream of the HIV-1 5′-LTR in the proximity of the sequence targeted by the gRNA. The IKZF1 binding site is composed of a consensus sequence (TGGGAA/T) and at least one more extra core site (GGGA) in a 40 bp range (Li *et al.*, 2011). (**E**) ChIP-qPCR analysis with IKZF1 antibody in latent cells and PMA stimulated J-Lat 11.1 cells as indicated. (**F**) ChIP qPCR analysis with IKZF1 antibody in J-Lat 11. 1 cells transduced with scramble shRNA (shControl) and shIKZF1. Data in (E) and (F) are presented as % input, error bars represent the standard deviation (SD) of two separate real-time PCR measurements. (G–I) ChIP-qPCR using antibodies specific for distinct histone marks in J-Lat 11. 1 cells transduced with scramble shRNA (shControl) and shIKZF1. Total histone H3 (**G**), H3K27me3 (**H**), H3K4me3 (**I**). Total histone H3 data (G) are represented as % input mean (±SD), histone marks data (H, I) are expressed as fold change over H3 signal (±SD). (**J**) ChIP-qPCR analysis with SUZ12 in J-Lat 11. 1 cells transduced with scramble shRNA (shControl) and shIKZF1. (**K**) ChIP-qPCR analysis with CBX8 in J-Lat 11. 1 cells transduced with scramble shRNA (shControl) and shIKZF1. Data in (J) and (K) are presented as % input, error bars represent the standard deviation (SD) of two separate real-time PCR measurements. The ChIP analysis presented (E–K) are a representative experiment, biological replicate experiments are shown in Supplementary Figure S8. (**L**) Western blotting for SUZ12, IKZF1, CBX in J-lat 11 transduced with scramble shRNA (sh Control) and shIKZF1. α-Tubulin is used as a loading control.

Catchet-MS identified the IKAROS Family Zinc Finger 1 protein (IKZF1) as one of the factors to be exclusively associated with the repressed HIV-1 promoter (Figure 2F). IKZF1, a critical factor required for normal T cell development that can act both as a transcriptional repressor and activator (44–46), has been reported to regulate gene expression through its association with the nucleosome remodeling and deacetylase complex (NuRD) and the positive transcription elongation factor (P-TEFb) (46–50). Additionally, IKZF1-mediated gene silencing in T cells has been shown to be facilitated by its interaction with the Polycomb repressive complex 2 (PRC2), which promotes histone H3 lysine 27 trimethylation, a mark of transcriptionally silent chromatin (51). Consistent with its role as a repressive factor in T cells, depletion of IKZF1 in our functional testing screen using independent shRNA clones (Figure 3A, B, Supplementary Figure S4A) led to the strongest reactivation of HIV-1 expression (5–10-fold), as measured by the increase of GFP-positive cells in flow cytometry and qRT-PCR for HIV-1 genes (Figure 3B, C, Supplementary Figure S4B). To ensure that the observed latency reversal is not subject to clonal effects in J-Lat 11.1 cells, we depleted IKZF1 in J-Lat A2 cells, harboring an integrated HIV 5′ LTR-driven GFP reporter and observed similar reversal of HIV-1 latency (Supplementary Figure S4C, D).

The IKZF family consists of several family members (49,52), although IKZF1 is the only family member detected in our mass spectrometry data. We assessed the expression of the other IKZF family members in Jurkat cells (RNA sequencing (53)) and proceeded with shRNA mediated depletion of IKZF2 and IKZF5, which are expressed in Jurkat cells (Supplementary Figure S5A). Depletion of IKZF2 and IKZF5 also resulted in HIV-1 latency reversal, although less pronounced compared to IKZF1 (Supplementary Figure S5B). These results are consistent with the lower levels of expression of IKZF2 and IKZF5 in T cells, compared to IKZF1, and with the well-established heterodimeric interactions among IKZF family members (54–56). We next sought to confirm and examine the association of IKZF1 with the latent HIV-1 LTR. Downstream of the 5′LTR, at position +818/+864 we identified a putative IKZF binding site, which is composed of a consensus TGGGAA/T sequence and two extra GGGA core sites (57), (Figure 3D). To assess IKZF1 binding at the predicted sites, we performed ChIP-qPCR experiments in latent and PMA treated J-Lat 11.1 cells using antibodies specific for IKZF1 (Figure 3E). In agreement with the Catchet-MS data, ChIP experiments in latent J-Lat 11.1 cells demonstrated IKZF1 enrichment between HIV-1 HS2 and Nuc2, (Figure 3E). Importantly, activation of HIV-1 transcription by PMA treatment abrogated IKZF1 binding (Figure 3E and Supplementary Figure S6A), consistent with the notion that IKZF1 may be important for maintenance of HIV-1 transcriptional repression. To confirm the specificity of IKZF1 binding, we also performed ChIP in control J-Lat 11.1 cells, infected with a scramble shRNA vector (Sh Control), and in cells infected with an shRNA vector targeting the IKZF1 mRNA (sh IKZF1). Our data show enrichment of IKZF1 binding over its predicted binding region in the sh Control cell line together with a dramatic loss of binding upon shRNA mediated depletion in the IKZF1 depleted cells (Figure 3F and Supplementary Figure S6B), thus confirming the specificity of the antibody. To further control for the specificity of the IKZF1 signal we also monitored enrichment of IKZF1 at endogenous IKZF1 targets previously reported to be enriched for IKZF1 binding in B cells (58) (Supplementary Figure S6C).

We then examined the effect of IKZF1 depletion on the chromatin state of the HIV 5′LTR region by histone ChIP-qPCR experiments using antibodies against the active chromatin modification H3 lysine 4 tri-methylation (H3K4me3), the repressive chromatin modifications H3 lysine 27 tri-methylation (H3K27me3) and H3 lysine 9 tri-methylation (H3K9me3), using the total histone H3 signal for normalization (Figure 3G–I; Supplementary Figure S7A–D).

Upon IKZF1 depletion the chromatin state is characterized by a drastic loss of enrichment for the H3K27me3 mark (Figure 3H; Supplementary Figure S7B), a moderate increase of the H3K4me3 mark (Figure 3I; Supplementary Figure S7C) and a concomitant decrease in the repressive chromatin mark H3K9me3 (Supplementary Figure 7SD). Similar data were obtained upon IKZF1 depletion in J-Lat A2 cells (Supplementary Figure S7E–I), thus confirming that the changes in chromatin states are not subject to clonal effects.

We also assessed the histone modification profile in latent and PMA stimulated J-lat 11.1 cells (Supplementary Figure S8A–D). Mimicking what was observed upon IKZF1 depletion and consistent with literature (3,59–61), treatment with PMA led to an increase in H3K4 tri-methylation (Supplementary Figure S8B) and a concomitant decrease in the repressive chromatin marks H3K27me3 (Supplementary Figure S8C) and H3K9me3 (Supplementary Figure S8D). All in all, we observed a general trend that indicates that the HIV-1 promoter is remodeled to a more active state upon IKZF-1 depletion.

In T cells, IKZF1 is necessary for mediating gene silencing through the recruitment of the Polycomb repressive complex 2 (PRC2) and the deposition of the H3K27me3 mark (51). Interestingly, our Catchet-MS experiment identified SUZ12, a core subunit of the PRC2 complex, and CBX8, a subunit of the Polycomb repressive complex 1 (PRC1) that acts as a reader of the H3K27me3 modification (62–64) to be also associated with the HIV-1 5′LTR in its latent state. We therefore conducted ChIP experiments to probe for the IKZF-1 mediated binding of SUZ12 to the region to verify the hypothesis that IKZF1 may be necessary for recruitment of PRC2 complex to the HIV-1 5′LTR. We additionally examined the recruitment of CBX8 to check whether the reduction in H3K27me3 mark deposition observed in absence of IKZF1 affects CBX8 binding. Consistent with this hypothesis, our data shows that depletion of IKZF1 leads to reduced enrichment of SUZ12 (Figure 3J and Supplementary Figure S8E) and CBX8 (Figure 3K) over the HS2 5′LTR region. Importantly, as shown by western blotting, depletion of IKZF1 does not lead to decreased levels of expression of SUZ12 and CBX8, suggesting that the effects observed are exclusively consequence of the absence of IKZF1 recruitment to the region (Figure 3L). When we treated IKZF1 depleted cells with EZH2 inhibitor EPZ6438 we observed reduced efficacy in reactivating HIV-1 latency as compared to cells containing control shRNA (Supplementary Figure S8F), which supports the notion that IKZF1 represses the HIV-1 LTR in a PRC2 dependent manner.

Lastly, we aimed to assess the levels of IKZF1 expression in the target memory CD4+ T cells that harbor the HIV-1 provirus and constitute the main HIV-1 cellular reservoir. Using an array gene expression dataset (65) corresponding to flow cytometry sorted Naïve, Central/transitional memory and Effector memory cells from two healthy donors, we observed similar levels of IKZF-1 expression in memory and naïve CD4+ T cells for both donors as assessed by two different probes (Supplementary Figure S8G).

### Targeting IKZF1 by iberdomide treatment reverses HIV-1 latency in *ex vivo* infected primary CD4+ T cells

Thalidomide-derived immunomodulatory drugs (IMIDs), have been shown to cause selective ubiquitination and degradation of IKZF1 and its related family member IKZF3 (66,67). The mechanism of action for IMID activity lies in their affinity for cereblon (CRBN), which is part of the cullin-ring finger ligase-4 cereblon (CRL4^CRBN^) E3 ubiquitin ligase complex. iberdomide is a novel compound with higher affinity for CRBN and the lowest IC50 of all thalidomide analogues, and is currently under clinical development for the treatment of systemic lupus erythematosus (SLE) and relapsed/refractory multiple myeloma (RRMM).

Binding of iberdomide to CRBN modulates the E3 ubiquitin ligase activity of the complex, increasing its affinity for IKZF1 and IKZF3, leading to their ubiquitination and proteasome-dependent degradation. Therefore, we set out to establish if iberdomide treatment in latent HIV-1 infected cells would induce degradation of IKZF1 and lead to HIV-1 latency reversal.

**Figure 4.**
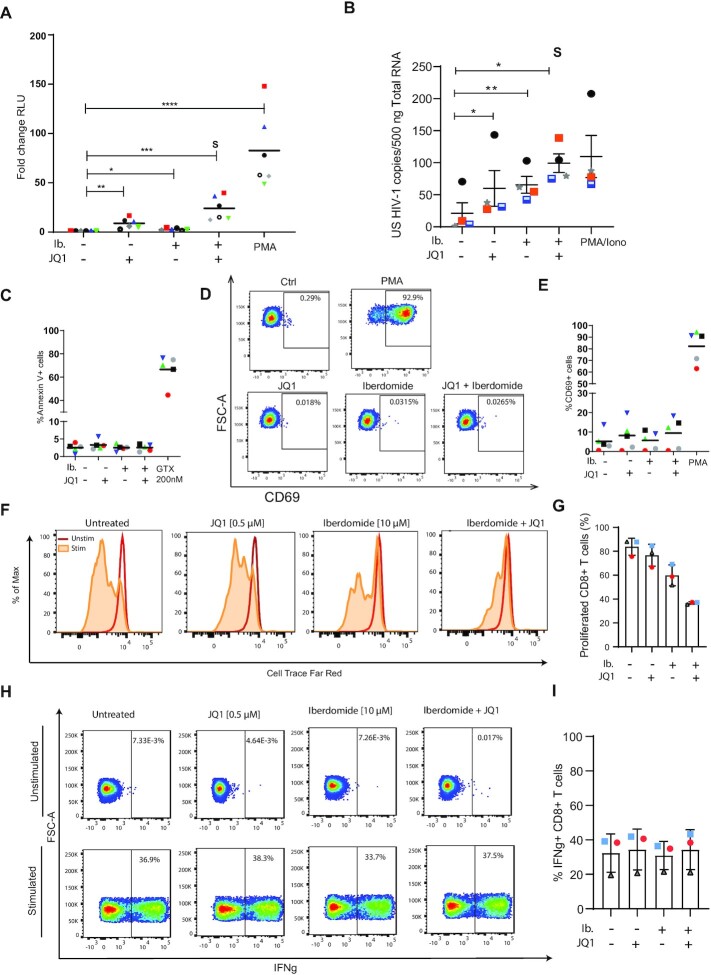
Targeting Targeting IKZF1 by iberdomide treatment reverses latency in primary CD4+ T cells obtained from cART suppressed HIV-1 infected patients with minimal effect on toxicity and effector function. (**A**) The latency reversal activity of iberdomide alone (10μM) and in combination with JQ1 (500nM) was tested, in primary human CD4+ T cells *ex vivo* infected to harbour latent HIV-1. The dot plot in panel A shows the fold increase in luciferase activity after treatment as indicated. Each dot represents a single measurement while the black horizontal lines represent the average fold increase for each treatment in the pool of donors. Experiments were performed in duplicate using cells obtained from 6 healthy donors. Statistical significance was determined by repeated measures one-way ANOVA on the log-transformed fold changes followed by Tukey's multiple comparison test: *P < 0.05, ** *P* < 0.01, *** *P* < 0.001, ****P < 0.0001. (**B**) Graph panel showing the average levels of unspliced cell-associated HIV-1 RNA in CD4+ T cells isolated from four HIV-1 infected, aviremic participants upon treatment with iberdomide (10 μM), JQ-1 (500 nM) or combination as measured by nested qPCR. PMA/Ionomycin was used as a positive control. CD4+ T cells were isolated from PBMCs from HIV-1 infected donors and treated as indicated for 24 h. S represents synergism, calculated by using the coefficient of drug interaction (CDI) method. Statistical significance was calculated using paired two-tailed *t*-test: *P < 0.05, ** *P* < 0.01. (**C**) Percentage of cells expressing the Annexin V marker of apoptosis in primary CD4+ T cells treated with iberdomide (10 μM), JQ-1 (500 nM) and the combination of both compounds for 24 h. Treatment with a toxic concentration of Gliotoxin (GTX) 200 nM was used as a positive control. Experiments were performed in uninfected cells obtained from five healthy donors, represented by the dots. (**D**) Representative flow cytometry plot of extracellular CD69 marker staining analysis in primary CD4+ T cells, upon treatment with iberdomide (10 μM), JQ-1 (500 nM) or a combination of both compounds for 24 h. CD69 expression was assessed by extracellular staining and analyzed by flow cytometry. (**E**) Percentage of cells expressing the CD69 marker of cell activation in primary CD4+ T cells from five healthy donors as described in (E). Treatment with PMA was used as a positive control. (**F**) Representative histogram of proliferative capacity of unstimulated or aCD3/CD28 stimulated CD8+ T cells in the presence or absence of LRAs. Cells were stained with a proliferation dye and analyzed 72 h later by flow cytometry. Dividing cells show decreased intensity of proliferation dye as it becomes diluted upon cell division. (**G**) Percentage of proliferated CD8+ T cells from three healthy donors as described in (G). (**H**) Representative flow cytometry plots of IFNg production analysis in unstimulated and stimulated primary CD8+ T cells after treatment with LRAs. Cells were treated as indicated for 18 h followed by PMA/Ionomycin (50 ng/1 μM) stimulation for 7 h in the presence of a protein transport inhibitor or remained unstimulated. IFN-g production was assessed by intracellular staining and analyzed by flow cytometry. Numbers in the plot show percentage of IL-2 producing cells. (**I**) Percentage of INFg producing CD8+ T cells from three healthy donors as described in (H).

As the main reservoir of latent HIV-1 in infected individuals comprises resting CD4+ T cells, we *ex vivo* infected, without prior activation, CD4+ T cells obtained from healthy donors with a defective full-length HIV-1 virus harboring a luciferase reporter to establish latent infections (Supplementary Figure S9A) (39). Treatment of latent HIV-1-infected primary CD4 + T cells with iberdomide for 48 h resulted in moderate but significant (*P* < 0.05) reversal of latency, as observed by an increase in the mean luciferase activity compared to the untreated control (Figure 4A) in all six donor derived primary CD4+ T cells examined. Bromodomain end extra-terminal domain (BET) inhibitors have been reported to act synergistically with IMIDs in treatment of refractory forms of multiple myeloma (68,69). Therefore, we examined whether these compounds also synergize in the context of HIV-1 latency reversal. Indeed, co-treatment with iberdomide and JQ1 resulted in a robust and synergistic HIV-1 latency reversal (Figure 4A). To confirm that treatment with iberdomide results in degradation of IKZF1 at the protein level, we performed western blot analysis of primary CD4+ T cells treated for 48 h with iberdomide. As expected, treatment with iberdomide, alone or in combination with JQ1, resulted in degradation of IKZF1, as shown in Supplementary Figure S9B. Importantly, treatment with iberdomide did not affect IKZF1 expression at the level of transcription (Supplementary Figure S9C), consistent with the known post-transcriptional mechanism by which IMIDs target IKZF1 for CRBN mediated degradation. Conversely, mRNA levels of the endogenous IKZF1 targets p21 and cMyc (70,71) were significantly affected by the treatment (Supplementary Figure S9C).

To validate our findings in a more relevant setting, we tested the efficacy of iberdomide in CD4+ T cells obtained from virologically suppressed HIV-1 patients. Treatment with iberdomide alone resulted in a significant increase in cell-associated unspliced HIV-1 RNA in all four donors compared to control, and was comparable to that observed after treatment of cells with the BET inhibitor JQ1 (Figure 4B). Remarkably, in cells derived from HIV-1 infected, aviremic participants we also observe a robust, synergistic increase in cell-associated HIV-1 RNA upon co-treatment with iberdomide and JQ-1 (Figure 4B). As observed in primary CD4 + T cells, treatment of iberdomide alone or in combination with JQ-1 in cells derived from aviremic HIV infected participants led to changes in gene expression of the IKZF1 targets p21 and c-Myc (Supplementary Figure S9D).

Confounding factors for potential clinical use of candidate LRAs is toxicity and the possibility of inducing unwanted global immune activation, thus leading to serious adverse effects. Potential toxicity and immune activation caused by iberdomide treatment in primary CD4+ T cells was determined by Annexin V and CD69 staining, followed by flow-cytometry. CD4+ T cell viability, measured by Annexin V staining, following iberdomide treatment alone or in combination with JQ-1 was not significantly affected (Figure 4C; Supplementary Figure S10A, B); neither did the treatment cause global T cell activation, as measured by extracellular CD69 staining (Figure 4D, E; Supplementary Figure S10B, C).

HIV-1 reactivation strategies that aim for a cure will require modulation or boosting of the immune compartment to promote viral clearance. Given the known immunomodulatory role of iberdomide and Thalidomide-derived drugs, we investigated the effects of iberdomide treatment alone or in combination with JQ-1 on T cell proliferation and functionality. As previously reported in the literature, and consistent with their anti-lymphoproliferative capacity (72), we observed decreased proliferation upon treatment of CD4 + and CD8 + primary T cells (Figure 4F and G; Supplementary Figure S10D and E). Finally, we assessed the effect of iberdomide on T cell functionality, by measuring cytokines IFNγ and IL-2 in CD4 + and CD8 + primary T cells. Iberdomide treatment alone or in combination with JQ-1 does not hamper the production of cytokines IFNγ (Figure 4H and I; Supplementary Figure S11A and B) and IL-2 (Supplementary Figure S11C-F) upon stimulation of CD4+ or CD8+ T cells and, therefore does not diminish T cell functionality. On the contrary, and consistent with the literature (73), we observe an increase in the production of IL-2 upon treatment with the LRAs (Supplementary Figure S11C–F).

### Targeting IKZF1 by FDA-approved thalidomide analogues reverses latency in cells obtained from cART suppressed HIV-1 infected patients and synergizes with other LRAs

Although clinically advanced, iberdomide is yet to be approved by drug administrations and, therefore, cannot be presently applied in the clinic. Other thalidomide analogues, namely lenalidomide and pomalidomide, are FDA-approved drugs employed in the treatment of relapsed and refractory multiple myeloma (MM), leading to improved patient survival rates. In addition, pomalidomide is also approved for the treatment of AIDS-related Kaposi sarcoma and has proven to be well tolerated in people living with HIV, without causing drug-drug interactions with ART (74). These drugs have thus high potential for translational studies and direct use in the clinic, and are worth investigating in the context of HIV-1 latency reversal and cure studies.

**Figure 5.**
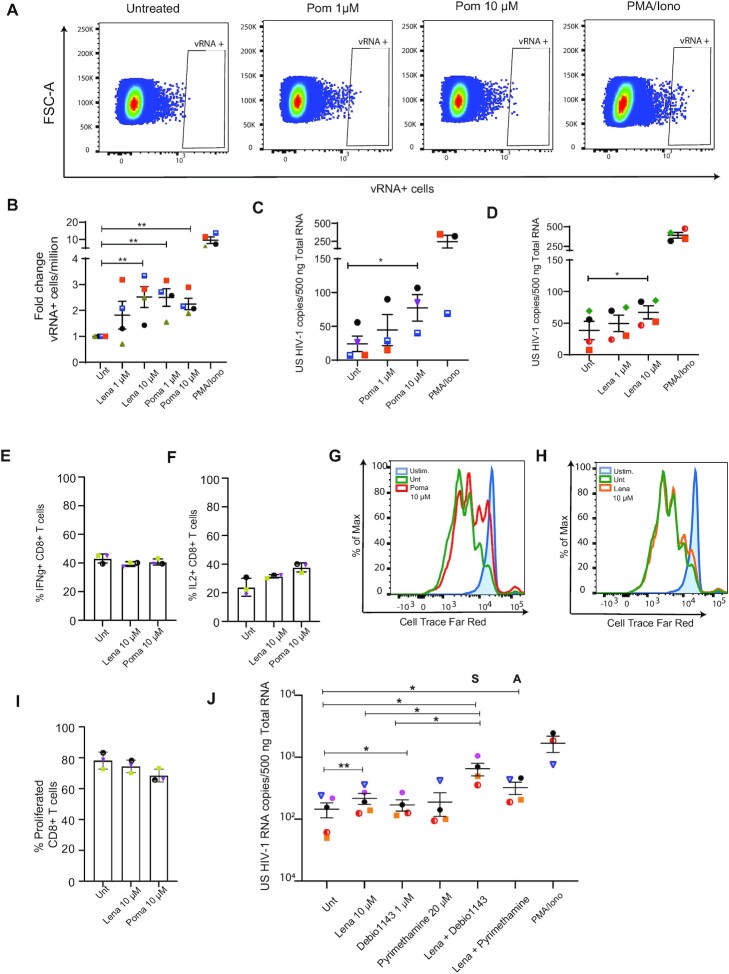
FDA-approved thalidomide analogues pomalidomide and lenalidomide reactivate HIV-1 latency and synergizes with other LRAs in cells obtained from HIV-1 infected donors. (**A**) Representative FISH-Flow dot plots of CD4+ T cells from HIV-1 infected donors *ex vivo* treated with pomalidomide at 1 and 10 μM concentration for 24 h. (**B**) Graph panel showing the fold increase in vRNA+ cells/million in *ex vivo* treated CD4+ T cells from HIV-1 infected donors relative to Untreated control as measured by FISH-Flow (A). Cells were treated with pomalidomide or lenalidomide at the indicated concentrations for 24 hours and vRNA + production was analyzed by FISH-Flow. Error bars represent the mean and standard deviation of independent experiments. Statistical significance was calculated using paired Wilcoxon test: * *P* < 0.05, ** *P* < 0.01. (**C, D**) Changes in cell-associated unspliced HIV-1 RNA in CD4+ T cells isolated from HIV-1 infected donors after treatment with pomalidomide (C) and lenalidomide (D). CD4+ T cells were isolated from PBMCs from HIV-1 infected donors and treated as indicated for 24 h. Error bars represent the mean and the standard deviation. Statistical significance was calculated using paired two-tailed *t* test: * *P* < 0,05. (E, F) Percentage of INFg (**E**) and IL2 (**F**) producing CD8+ T cells from three healthy donors upon treatment with lenalidomide and pomalidomide as indicated. Cells were treated as indicated for 18 h followed by PMA/Ionomycin (50 ng/1 μM) stimulation for 7 h in the presence of a protein transport inhibitor or remained unstimulated. IFN-g and IL2 production was assessed by intracellular staining and analyzed by flow cytometry. Numbers in the plot show percentage of IFNg and IL2 producing cells. (G, H) Representative histogram of proliferative capacity of unstimulated or aCD3/CD28 stimulated CD8+ T cells in the presence or absence of pomalidomide (**G**) or lenalidomide (**H**). Cells were stained with a proliferation dye and analyzed 72 h later by flow cytometry. Dividing cells show decreased intensity of proliferation dye as it becomes diluted upon cell division. (**I**) Percentage of proliferated CD4+ T cells from three healthy donors in the presence of pomalidomide and lenalidomide as described in (G, H). (**J**) Synergistic reactivation of HIV-1 latency by lenalidomide in combination with Debio 1143 and Pyrimethamine in CD4+ T cells obtained from HIV-1 infected donors as measured by cell associated unspliced HIV-1 RNA. CD4+ T cells were isolated from PBMCs from HIV-1 infected donors and treated as indicated for 18–24 h. Error bars represent the mean and the standard deviation. Statistical significance was calculated using paired two-tailed *t* test: * *P* < 0,05, ** *P* < 0,01. S represents synergism, A represents additive effects calculated by using the coefficient of drug interaction (CDI) method.

In order to characterize the role of FDA-approved pomalidomide and lenalidomide in HIV-1 latency reversal we assessed their efficacy in *ex vivo* treated primary CD4 + T cells obtained from aviremic HIV1-infected donors (Table 1). Cells were treated for 24 h with different concentrations of pomalidomide and lenalidomide and latency reversal was analyzed both by FISH-Flow using probes against the viral RNA (vRNA) that target the *gag/pol* region and CA HIV-1 RNA targeting the US HIV-1 RNA. Our results show that both pomalidomide and lenalidomide are able to significantly reverse HIV-1 latency in *ex vivo* treated CD4+ T cells from people living with HIV (PLWH) as observed by an increase in vRNA+ cells upon treatment (Figure 5A and B; Supplementary Figure S12A). Moreover, we show that pomalidomide (Figure 5C; Supplementary Figure S12B) and lenalidomide (Figure 5D; Supplementary Figure S12C) increase the levels of cell associated unspliced (CA US) HIV-1 RNA in CD4+ T cells from PLWH. In addition, we confirmed by western blot that treatment with pomalidomide and lenalidomide leads to degradation of IKZF1 protein in CD4+ T cells after 24 h of treatment (Supplementary Figure S12D, E).

**Table 1.** Clinical Information table corresponding to the HIV-1 infected study participants

	Donor 1	Donor 2	Donor 3	Donor 4	Donor 5	Donor 6	Donor 7	Donor 8	Donor 9	Donor 10
**Symbol**	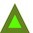	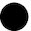	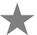	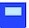	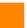	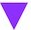	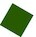	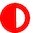	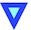	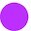
**Sex**	F	M	M	M	M	M	M	M	M	M
**Time from HIV-1 diagnosis to leuk. (months)**	55	85	52	86	108	19	384	264	77	250
**ART initiated during acute HIV-1**	No	No	Yes (Fiebig 6)	Yes (Fiebig 6)	No	Yes (Fiebig 4)	No	No	No	No
**Time on ART (months)**	55	72	52	86	81	19	288	204	77	243
**Time with continues HIV-1 RNA < 50 copies/ml (months)**	>12	>12	>12	>12	>12	>12	>12	>12	>12	>12
**CD4+ at leukapheresis (mm^3^)**	620	490	1540	990	970	870	570	750	950	470

To assess toxicity induced by treatment with pomalidomide and lenalidomide we treated primary CD4+ T cells obtained from healthy donors with different concentrations of these drugs for 24 h and stained for Annexin V as a marker of apoptosis. In addition, we also stained for the extracellular CD69 marker in order to assess global T cell activation caused by thalidomide analogues. Similar to our results with iberdomide, we show that pomalidomide and lenalidomide do not cause loss of viability or apoptosis and do not induce global T cell activation (Supplementary Figure S13A and B). Because of their immunomodulatory role described in literature we also aimed to assess the effects of pomalidomide and lenalidomide treatment in CD4+ and CD8+ T cell proliferation and functionality. As shown with iberdomide, both pomalidomide and lenalidomide treatment did not hamper T cell functionality as measured by intracellular production of IFNg and IL-2 upon PMA/Ionomycin stimulation (Figure 5E, F; Supplementary Figure S13C–E), and only pomalidomide caused a slight but non-significant decrease in proliferative capacity of primary CD4+ and CD8+ T cells (Figure 5G–I and Supplementary Figure S13F–H).

Finally, we aimed to assess whether the Thalidomide analogue lenalidomide, widely used for the treatment of immunopathies, is able to synergize with other known FDA-approved LRAs to achieve higher induction of HIV-1 transcription. We reasoned that the highest synergy would be achieved by combinations of compounds that target distinct mechanisms regulating HIV-1 latency. We chose to explore combinations of the de-repressors lenalidomide and pyrimethamine (BAF inhibitor) (40) and non-canonical NFKb activator Debio1134 (75) .We tested these compounds alone or in combination with lenalidomide in CD4+ T cells obtained from aviremic HIV-1 infected individuals. Our results demonstrate that lenalidomide is able to synergize with non-canonical NF-κB activator Debio 1134, and has additive effects when combined with the BAF inhibitor Pyrimethamine as assessed by Coefficient of Drug Interaction synergy score (Figure 5J).

In conclusion, we demonstrate that FDA-approved Thalidomide analogues are able to reverse latency in *ex vivo* treated CD4+ T cells obtained from PLWH, synergize with other known FDA-approved LRAs and are therefore promising candidates for potential direct use in the clinic. The immunomodulatory effects of these two drugs together with the fact that they are orally available and have proven to be safe in PLWH, prompts for further studies in the context of HIV-1 latency reversal and cure strategies.

## DISCUSSION

Locus-specific proteomics is a developing field that still represents tremendous biochemical challenges. The high signal to noise ratio together with the low abundance of single-loci, represent a significant hurdle to overcome in locus-specific strategies to identify the complete repertoire of factors bound to a single locus *in vivo* (27). Here, in search of novel determinants of HIV-1 latency, we designed a targeted discovery strategy based on proteomics, which has allowed us to describe the proteins associated with the HIV-1 5′LTR in its latent and active states. Catchet-MS is based upon a reverse-ChIP strategy and couples CRISPR/dCas9 targeting and purification of a genomic locus to an additional biochemical histone affinity step, to enrich for chromatin-associated dCas9 locus-bound complexes. The advantage of the Catchet-MS approach is its use of dCas9 as a bait which renders the system suitable for the study of single genomic loci in live cells, without the need to modify the site of interest. Additionally, introduction of the histone-based affinity purification step ensures that Catchet-MS effectively removes unwanted background originating from cellular contaminants and non-localized bait molecules from the mass spectrometry analysis. Crucially, we performed Catchet-MS both in a latent and in a re-activated HIV-1 infected isogenic cell line and focused our analysis exclusively on proteins that were differentially bound to the latent versus the transcriptionally active HIV 5′LTR, a strategy which allows for stringent removal of potential nonspecific background without the need of additional negative controls. Limitations of Catchet-MS, common to other locus specific enrichment strategies are its large scale, limiting the technique to cell lines and inability to provide a comprehensive and specific set of locus bound proteins. Catchet-MS should thus be considered a discovery tool to identify proteins distinctively associated with a genomic region of interest in two functionally disparate states, including transcriptional on-off state, as with the latent and active HIV-5′LTR here, states of differentiation, or distinct genomic SNPs.

Aware of these limitations, we verified the locus specificity of our dCas9 bait targeting by ChIP sequencing, made use of a large input (∼3 billion cells) and introduced a two-step (V5/histones) affinity purification, increasing sensitivity and specificity over a single-step affinity purification, and used stringent filtering and exclusive selection of MS hits detected with high confidence. Importantly, we describe the distinct proteome of latent versus activated HIV-1 5′LTR bound factors and not that of the total HIV 5′LTR bound proteome. In our analysis, the chromatin obtained from cells containing an activated HIV-1 5′LTR serves as a control for the chromatin obtained from cells harboring latent HIV-1 5′LTR, and vice versa. Using this approach, the majority of the proteins common to both conditions, including the dCas9 bait, potential non-specific contaminants, but also previously reported functionally relevant factors such as EED (76,77), MTA2 (78) and UCHL5 (79), are excluded from further analysis, thus confirming the stringency of the approach.

Among the list of interactors bound to the HIV-1 5′LTR locus, in addition to the well-established candidates, we find a number of novel interactors of the HIV-1 promoter (5′LTR) in its latent and active states as shown in Figure 3F and Supplementary Table S2. Within the list of latent 5′LTR associated factors, we find proteins belonging to chromatin remodeling complexes with well-established roles in HIV-1 latency including the Polycomb repressive complex1 (PRC1), Polycomb repressive complex 2 (PRC2), the SWItch/Sucrose Non-Fermentable complex (SWI/SNF) and the Nucleosome Remodeling Deacetylase (NuRD) complexes (4,80). We also find sequence-specific transcription factors previously reported to repress HIV-1 transcription (YY1 (81) and POU2F1 (82)), proteins structurally bound to chromatin (TOP2A, PDS5A, PDS5B), and proteins described to promote viral latency (SUPT16H, DKC1) (83,84).

Our approach specifically identified factors differentially bound to the latent and activated LTR and therefore potentially regulate the HIV-1 transcriptional and co-transcriptional status through a direct interaction with the HIV-1 promoter. In a different methodological approach, by using a prey-bait yeast system Pedro et al also identified novel HIV-1 LTR interactors. In other studies, such as CRISPR KO based screens (85,86), gene-trap mutagenesis (87) or (single cell)RNA-seq analysis on sorted latent vs active infected cells (88,89) the screen results identified putative targets that regulate HIV-1 latency and latency reactivation not only via direct binding to the LTR but also via putative indirect molecular mechanisms.

Beyond the differences in methodological approach and latency models, we found common hits identified by Catchet-MS and those in previously published studies. These include the helicase DDX5 (89), members of the TREX1 (90) and TREX2 complex (87), transcriptional regulators such as GON4L (86) and CBX3 (88), members of the proteasome complex (86), SP-related factors (91) and interferon related factors such as IRF2BP2 (87,91) and ISG15 (88). The identification of previously identified and characterized interactors serve as positive controls and justify the validity of our approach. As expected, upon HIV-1 transcriptional activation, a much larger number of ribonucleoproteins, splicing factors as well as proteins involved in mRNA processing and nuclear-cytoplasmic transport are identified by Catchet-MS, most likely recruited to the locus following transcriptional activation (Supplementary Table S3).

In search of putative novel molecular targets for HIV-1 latency reversal, we focused on factors identified to be significantly enriched or uniquely bound to the latent but not the activated HIV-1 LTR. Using shRNA-mediated depletion, we functionally validated a number of novel putative candidates in J-Lat 11.1 cells. Of 10 putative candidates tested, shRNA depletion of 5 factors (HP1BP3, IKZF1, CDC73, DKC1, PNN) resulted in HIV-1 latency reversal. Of these, the sequence-specific transcription factor IKZF1 stood out as an attractive candidate for which drug targeting is also available (66,67). IKZF1, a critical factor for lymphoid lineage specification in hematopoietic stem cells, suppresses the stem cell and myeloid programs and primes the expression of lymphoid specific genes (45). IKZF1 is also an important regulator of T cell function (52,92). Mechanistically, IKZF1 has a dual role in transcription, acting, in a context-dependent manner, both as a repressor and an activator (44–46). In an activating capacity, IKZF1 can restrict the activity of the NuRD complex, promoting chromatin accessibility (46,48–50). IKZF1 has also been proposed to act as an adaptor protein for the local recruitment of p-TEFb and the protein phosphatase 1α at IKZF1 target genes, facilitating transcription elongation (47). However, in T cells, IKZF1 has been shown to associate with PRC2 and is required for repression of Notch target genes and the hematopoietic stem cell program (51). IKZF1-mediated gene silencing may also depend on its interaction with the co-repressors CtBP, CtIP SWI/SNF-related complexes and HDAC-containing Sin3 complexes (49).

**Figure 6.**
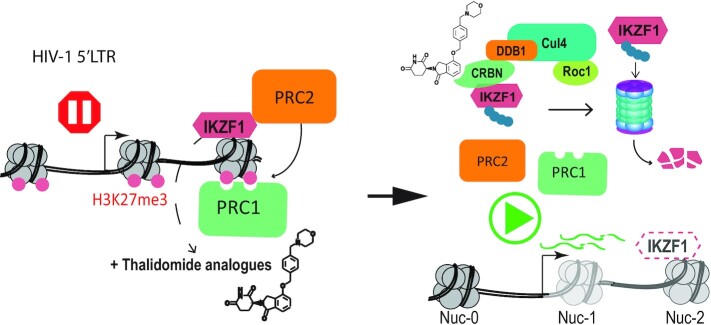
Proposed model for Thalidomide analogues mediated HIV-1 latency reversal. Thalidomide analogues binds to CRBN, a subunit of the CRL^CRBN^ E3 ubiquitin ligase complex that acts as a substrate adaptor. Thalidomide biding induces recruitment of IKZF1 and its ubiquitination by the ligase. In latently infected cells, ubiquitination of IKZF1 and its degradation by the proteasome results in a decrease in IKZF1 levels, impaired LTR recruitment of PRC2 and PRC1, leading to chromatin de-repression and latency reversal.

Previous evidence points to a model in which, in T-cells, IKZF1 regulates the epigenetic silencing of IKZF1 target genes via recruitment of the PRC2 complex (51). The regulatory role of PRC2 at the latent HIV-1 promoter on chromatin compaction, recruitment of PRC1, and repression of HIV-1 transcription is well established (49,62,80). Consistent with these reports, Catchet-MS identified SUZ12, a core subunit of the PRC2 complex, and CBX8, a subunit of the PRC1 complex, to be associated with the HIV-1 5′LTR in its latent state (Figure 3F). Depletion of IKZF1 led to reduced SUZ12 and CBX8 enrichment over the HS2 region. Our results are consistent with a model in which IKZF1 directly recruits PRC2 to the HIV-1 5′LTR, as previously described at endogenous target genes in T cells (51). The PRC2-modified, H3K27 trimethylated region subsequently serves as a docking site for and results in recruitment of PRC1 (Figure 6).

IMIDs, such as the thalidomide analogues lenalidomide, pomalidomide and iberdomide promote ubiquitin-dependent proteosomal degradation of IKZF1 and IKZF3 by redirecting the substrate specificity of the CRL4^CRBN^ ubiquitin ligase complex (67,93). Among these drugs, iberdomide (CC-220) is a novel compound with the highest reported specificity for CRBN and the lowest IC50, currently in a phase 2 clinical trial for systemic lupus erythematosus (SLE) and multiple myeloma (MM). Due to its high potency we selected iberdomide for treatment of CD4 + T cells obtained from cART suppressed aviremic participants. Iberdomide mediated depletion of IKZF1 was accompanied by pronounced reversal of HIV-1 latency, with no significant associated toxicity. BET inhibitors, such as JQ1, represent a class of compounds previously reported to synergize with IMIDs in inhibiting the growth of refractory forms of MM (68,69). As BET inhibitors are also a well-established class of HIV-1 LRAs, we examined the latency reversal effects of combination treatment with iberdomide and JQ1. Remarkably, co-treatment of latently infected primary CD4+ T cells results in strong and synergistic induction of HIV-1 transcription in cells obtained from HIV infected cART suppressed aviremic patients. Thus, similar to what was observed in the treatment of resistant MM, combination of these two classes of compounds also leads to synergism in the context of HIV-1 latency reversal.

Although clinically advanced, iberdomide is yet to be approved by international drug approval bodies. On the other hand, pomalidomide and lenalidomide are FDA-approved compounds currently in use for the treatment of immune disorders such as MM or other lymphopathies (72); therefore, we aimed to investigate their role in HIV-1 latency reversal and potential effects on the immune system. We show that both pomalidomide and lenalidomide treatment of CD4+ T cells obtained from HIV-1 infected, aviremic donors leads to reactivation of HIV-1 latency without causing added toxicity or global immune activation in primary CD4 + T cells. Importantly, we show that lenalidomide is able to synergize with other FDA-approved LRAs such as non canonical NFKb activator Debio 1134 (75), and has additive effects when combined with BAF inhibitor Pyrimethamine (40).

The role of Thalidomide and its analogues in modulating the immune system has been very well studied and described in the past, in the context of lympho and myeloproliferative immune pathologies (72). Consistent with what is reported in the literature (72), in this study we show that iberdomide and the FDA-approved thalidomide analogue pomalidomide modulate the proliferation capacity of primary T cells. In addition, we demonstrate that the use of iberdomide does not affect T cell functionality, as measured by cytokine expression in CD4+ and CD8+ T cells and does not cause apoptosis or global immune activation. More importantly, high concentrations of pomalidomide and lenalidomide do not cause cytotoxicity or impair T cell functional response in primary CD4+ T cells obtained from healthy donors. Crucial to the potential applicability of LRA treatments alone or in combination in future clinical studies is the absence of detrimental effects on the overall T cell biology and the preservation of a functional cytotoxic compartment. Currently available LRAs, though not appearing to cause serious adverse effects in HIV-1 infected individuals, lead to significant cytotoxicity in CD4+ and CD8+ T cells and thus major impairment of cytotoxic immune responses (22). As viral clearance after latency reactivation depends mostly on cytotoxic CD8+ T cells, maintaining or boosting functional immune responses in HIV-1 cure strategies is of utmost importance for achieving long-term reduction in reservoir size. Thus, the role of Thalidomide analogues in modulating the immune system together with their LRA activity makes them of great interest for HIV-1 cure studies. In fact, previous clinical studies have shown that the use of pomalidomide leads to an increase in the total numbers of CD4+ and CD8+ T cells, decrease in senescence and a shift towards central memory T cells in HIV-1 infected individuals. Furthermore, IMIDs such as pomalidomide have been shown to enhance co-stimulation of virus specific CD8+ T cells (94) and, more importantly, treatment of pomalidomide and lenalidomide significantly expands and enhances the effector function of HIV-1 specific CD8+ T cells *in vitro* (95) and boosts immune responses in HIV-1 infected individuals by inducing chemotaxis-mediated recruitment of effector cells (96). Notably, several clinical trials have shown that pomalidomide and lenalidomide at high doses are safe and tolerable in HIV-1 infected patients in the context of AIDS-related morbidities, such as Kaposi sarcoma, or in HIV+ individuals suffering from lymphoma (74,97–99).

To conclude, FDA/EMA-approved thalidomide analogue compounds, such as pomalidomide and lenalidomide, which target IKZF1 for degradation and lead to HIV-1 latency reactivation while boosting HIV-1 specific CTL responses, represent attractive candidates for inclusion in proof of concept clinical studies aiming to reduce the latent HIV-1 reservoir. The specificity of the IMID/IKZF1-targeting pharmacological strategy for HIV-1 latency reversal may also be enhanced by combination treatment with other LRA class compounds, which we demonstrate to lead to synergistic latency reversal.

## DATA AVAILABILITY

Data corresponding to the CHIP-Seq experiment is now deposited in the GEO repository under GEO accession number GSE196059. The mass spectrometry proteomics data is deposited in PRIDE repository under accession number PXD032975. Lastly, the flow cytometry data corresponding to primary cell experiments is deposited in FlowRepository under accession number FR-FCM-Z56J.

## Supplementary Material

gkac407_Supplemental_Files

## References

[1] Siliciano J.M. , Siliciano R.F. The remarkable stability of the latent reservoir for HIV-1 in resting memory CD4+ T cells. J. Infect. Dis. 2015; 212:1345–1347. 25877551 10.1093/infdis/jiv219

[2] Deeks S.G. HIV: shock and kill. Nature. 2012; 487:439–440. 22836995 10.1038/487439a

[3] Rafati H. , Parra M., Hakre S., Moshkin Y., Verdin E., Mahmoudi T. Repressive LTR nucleosome positioning by the BAF complex is required for HIV latency. PLoS Biol. 2011; 9:e1001206. 22140357 10.1371/journal.pbio.1001206 PMC3226458

[4] Ne E. , Palstra R.J., Mahmoudi T. Transcription: insights from the HIV-1 promoter. Int. Rev. Cell Mol. Biol. 2018; 335:191–243. 29305013 10.1016/bs.ircmb.2017.07.011

[5] Pereira L.A. , Bentley K., Peeters A., Churchill M.J., Deacon N.J. A compilation of cellular transcription factor interactions with the HIV-1 LTR promoter. Nucleic Acids Res. 2000; 28:663–668. 10637316 10.1093/nar/28.3.663 PMC102541

[6] Mousseau G. , Valente S.T. Role of host factors on the regulation of tat-mediated HIV-1 transcription. Curr. Pharm. Des. 2017; 23:4079–4090. 28641539 10.2174/1381612823666170622104355 PMC5731639

[7] Ott M. , Geyer M., Zhou Q. The control of HIV transcription: keeping RNA polymerase II on track. Cell Host Microbe. 2011; 10:426–435. 22100159 10.1016/j.chom.2011.11.002 PMC3478145

[8] Chun T.W. , Justement J.S., Lempicki R.A., Yang J., Dennis G., Hallahan C.W., Sanford C., Pandya P., Liu S., McLaughlin M et al. Gene expression and viral prodution in latently infected, resting CD4+ T cells in viremic versus aviremic HIV-infected individuals. Proc. Natl. Acad. Sci. U.S.A. 2003; 100:1908–1913. 12552096 10.1073/pnas.0437640100 PMC149932

[9] Karn J. , Stoltzfus C.M. Transcriptional and posttranscriptional regulation of HIV-1 gene expression. Cold Spring Harb. Perspect. Med. 2012; 2:a006916. 22355797 10.1101/cshperspect.a006916 PMC3281586

[10] Lassen K.G. , Ramyar K.X., Bailey J.R., Zhou Y., Siliciano R.F. Nuclear retention of multiply spliced HIV-1 RNA in resting CD4+ T cells. PLoS Pathog. 2006; 2:e68. 16839202 10.1371/journal.ppat.0020068 PMC1487174

[11] Rojas-Araya B. , Ohlmann T., Soto-Rifo R. Translational control of the HIV unspliced genomic RNA. Viruses. 2015; 7:4326–4351. 26247956 10.3390/v7082822 PMC4576183

[12] Stoszko M. , Ne E., Abner E., Mahmoudi T. A broad drug arsenal to attack a strenuous latent HIV reservoir. Curr. Opin. Virol. 2019; 38:37–53. 31323521 10.1016/j.coviro.2019.06.001

[13] Elliott J.H. , Wightman F., Solomon A., Ghneim K., Ahlers J., Cameron M.J., Smith M.Z., Spelman T., McMahon J., Velayudham P. et al. Activation of HIV transcription with short-course vorinostat in HIV-infected patients on suppressive antiretroviral therapy. PLoS Pathog. 2014; 10:e1004473. 25393648 10.1371/journal.ppat.1004473 PMC4231123

[14] Archin N.M. , Eron J.J., Palmer S., Hartmann-Duff A., Martinson J.A., Wiegand A., Bandarenko N., Schmitz J.L., Bosch R.J., Landay A.L. et al. Valproic acid without intensified antiviral therapy has limited impact on persistent HIV infection of resting CD4+ T cells. AIDS. 2008; 22:1131–1135. 18525258 10.1097/QAD.0b013e3282fd6df4 PMC3863687

[15] Archin N.M. , Cheema M., Parker D., Wiegand A., Bosch R.J., Coffin J.M., Eron J., Cohen M., Margolis D.M. Antiretroviral intensification and valproic acid lack sustained effect on residual HIV-1 viremia or resting CD4+ cell infection. PLoS One. 2010; 5:e9390. 20186346 10.1371/journal.pone.0009390 PMC2826423

[16] Sogaard O.S. , Graversen M.E., Leth S., Olesen R., Brinkmann C.R., Nissen S.K., Kjaer A.S., Schleimann M.H., Denton P.W., Hey-Cunningham W.J. et al. The depsipeptide romidepsin reverses HIV-1 latency in vivo. PLoS Pathog. 2015; 11:e1005142. 26379282 10.1371/journal.ppat.1005142 PMC4575032

[17] Battivelli E. , Dahabieh M.S., Abdel-Mohsen M., Svensson J.P., TojalDa Silva I., Cohn L.B., Gramatica A., Deeks S., Greene W.C., Pillai S.K. et al. Distinct chromatin functional states correlate with HIV latency reactivation in infected primary CD4(+) T cells. Elife. 2018; 7:e34655. 29714165 10.7554/eLife.34655 PMC5973828

[18] Grau-Exposito J. , Luque-Ballesteros L., Navarro J., Curran A., Burgos J., Ribera E., Torrella A., Planas B., Badia R., Martin-Castillo M. et al. Latency reversal agents affect differently the latent reservoir present in distinct CD4+ T subpopulations. PLoS Pathog. 2019; 15:e1007991. 31425551 10.1371/journal.ppat.1007991 PMC6715238

[19] Grau-Exposito J. , Serra-Peinado C., Miguel L., Navarro J., Curran A., Burgos J., Ocana I., Ribera E., Torrella A., Planas B. et al. A novel single-cell FISH-Flow assay identifies effector memory CD4(+) T cells as a major niche for HIV-1 transcription in HIV-Infected patients. Mbio. 2017; 8:e00876-17. 28698276 10.1128/mBio.00876-17 PMC5513707

[20] Kim Y. , Anderson J.L., Lewin S.R. Getting the “Kill” into “Shock and kill”: strategies to eliminate latent HIV. Cell Host Microbe. 2018; 23:14–26. 29324227 10.1016/j.chom.2017.12.004 PMC5990418

[21] Rao S. , Lungu C., Crespo R., Steijaert T.H., Gorska A., Palstra R.J., Prins H.A.B., van Ijcken W., Mueller Y.M., van Kampen J.J.A. et al. Selective cell death in HIV-1-infected cells by DDX3 inhibitors leads to depletion of the inducible reservoir. Nat. Commun. 2021; 12:2475. 33931637 10.1038/s41467-021-22608-z PMC8087668

[22] Zhao M. , De Crignis E., Rokx C., Verbon A., van Gelder T., Mahmoudi T., Katsikis P.D., Mueller Y.M. T cell toxicity of HIV latency reversing agents. Pharmacol. Res. 2019; 139:524–534. 30366100 10.1016/j.phrs.2018.10.023

[23] Byrum S.D. , Raman A., Taverna S.D., Tackett A.J. ChAP-MS: a method for identification of proteins and histone posttranslational modifications at a single genomic locus. Cell Rep. 2012; 2:198–205. 22840409 10.1016/j.celrep.2012.06.019 PMC3408609

[24] Pourfarzad F. , Aghajanirefah A., de Boer E., Ten Have S., Bryn van Dijk T., Kheradmandkia S., Stadhouders R., Thongjuea S., Soler E., Gillemans N. et al. Locus-specific proteomics by TChP: targeted chromatin purification. Cell Rep. 2013; 4:589–600. 23911284 10.1016/j.celrep.2013.07.004

[25] Liu X. , Zhang Y., Chen Y., Li M., Zhou F., Li K., Cao H., Ni M., Liu Y., Gu Z. et al. In situ capture of chromatin interactions by biotinylated dCas9. Cell. 2017; 170:1028–1043. 28841410 10.1016/j.cell.2017.08.003 PMC6857456

[26] Waldrip Z.J. , Byrum S.D., Storey A.J., Gao J., Byrd A.K., Mackintosh S.G., Wahls W.P., Taverna S.D., Raney K.D., Tackett A.J. A CRISPR-based approach for proteomic analysis of a single genomic locus. Epigenetics. 2014; 9:1207–1211. 25147920 10.4161/epi.29919 PMC4169012

[27] Gauchier M. , van Mierlo G., Vermeulen M., Dejardin J. Purification and enrichment of specific chromatin loci. Nat. Methods. 2020; 17:380–389. 32152500 10.1038/s41592-020-0765-4

[28] Vermeulen M. , Dejardin J. Locus-specific chromatin isolation. Nat. Rev. Mol. Cell Biol. 2020; 21:249–250. 31996790 10.1038/s41580-020-0217-0

[29] Dejardin J. , Kingston R.E. Purification of proteins associated with specific genomic Loci. Cell. 2009; 136:175–186. 19135898 10.1016/j.cell.2008.11.045 PMC3395431

[30] Chen B. , Gilbert L.A., Cimini B.A., Schnitzbauer J., Zhang W., Li G.W., Park J., Blackburn E.H., Weissman J.S., Qi L.S. et al. Dynamic imaging of genomic loci in living human cells by an optimized CRISPR/Cas system. Cell. 2013; 155:1479–1491. 24360272 10.1016/j.cell.2013.12.001 PMC3918502

[31] Ma H. , Tu L.C., Naseri A., Huisman M., Zhang S., Grunwald D., Pederson T. CRISPR-Cas9 nuclear dynamics and target recognition in living cells. J. Cell Biol. 2016; 214:529–537. 27551060 10.1083/jcb.201604115 PMC5004447

[32] Kustatscher G. , Wills K.L., Furlan C., Rappsilber J. Chromatin enrichment for proteomics. Nat. Protoc. 2014; 9:2090–2099. 25101823 10.1038/nprot.2014.142 PMC4300392

[33] Chalkley G.E. , Verrijzer C.P. Immuno-depletion and purification strategies to study chromatin-remodeling factors in vitro. Methods Enzymol. 2004; 377:421–442. 14979043 10.1016/S0076-6879(03)77028-1

[34] Schwertman P. , Lagarou A., Dekkers D.H., Raams A., van der Hoek A.C., Laffeber C., Hoeijmakers J.H., Demmers J.A., Fousteri M., Vermeulen W. et al. UV-sensitive syndrome protein UVSSA recruits USP7 to regulate transcription-coupled repair. Nat. Genet. 2012; 44:598–602. 22466611 10.1038/ng.2230

[35] Sap K.A. , Bezstarosti K., Dekkers D.H.W., Voets O., Demmers J.A.A. Quantitative proteomics reveals extensive changes in the ubiquitinome after perturbation of the proteasome by targeted dsRNA-Mediated subunit knockdown in drosophila. J. Proteome Res. 2017; 16:2848–2862. 28665616 10.1021/acs.jproteome.7b00156

[36] Li H. , Durbin R. Fast and accurate long-read alignment with burrows-wheeler transform. Bioinformatics. 2010; 26:589–595. 20080505 10.1093/bioinformatics/btp698 PMC2828108

[37] Zhang Y. , Liu T., Meyer C.A., Eeckhoute J., Johnson D.S., Bernstein B.E., Nusbaum C., Myers R.M., Brown M., Li W. et al. Model-based analysis of ChIP-Seq (MACS). Genome Biol. 2008; 9:R137. 18798982 10.1186/gb-2008-9-9-r137 PMC2592715

[38] Schmittgen T.D. , Livak K.J. Analyzing real-time PCR data by the comparative C(T) method. Nat. Protoc. 2008; 3:1101–1108. 18546601 10.1038/nprot.2008.73

[39] Lassen K.G. , Hebbeler A.M., Bhattacharyya D., Lobritz M.A., Greene W.C. A flexible model of HIV-1 latency permitting evaluation of many primary CD4 T-cell reservoirs. PLoS One. 2012; 7:e30176. 22291913 10.1371/journal.pone.0030176 PMC3265466

[40] Stoszko M. , De Crignis E., Rokx C., Khalid M.M., Lungu C., Palstra R.J., Kan T.W., Boucher C., Verbon A., Dykhuizen E.C. et al. Small molecule inhibitors of BAF; a promising family of compounds in HIV-1 latency reversal. EBioMedicine. 2016; 3:108–121. 26870822 10.1016/j.ebiom.2015.11.047 PMC4739437

[41] Pasternak A.O. , Adema K.W., Bakker M., Jurriaans S., Berkhout B., Cornelissen M., Lukashov V.V. Highly sensitive methods based on seminested real-time reverse transcription-PCR for quantitation of human immunodeficiency virus type 1 unspliced and multiply spliced RNA and proviral DNA. J. Clin. Microbiol. 2008; 46:2206–2211. 18463204 10.1128/JCM.00055-08 PMC2446885

[42] Jordan A. , Bisgrove D., Verdin E. HIV reproducibly establishes a latent infection after acute infection of T cells in vitro. EMBO J. 2003; 22:1868–1877. 12682019 10.1093/emboj/cdg188 PMC154479

[43] Yukl S.A. , Kaiser P., Kim P., Telwatte S., Joshi S.K., Vu M., Lampiris H., Wong J.K. HIV latency in isolated patient CD4(+) T cells may be due to blocks in HIV transcriptional elongation, completion, and splicing. Sci. Transl. Med. 2018; 10:eaap9927. 29491188 10.1126/scitranslmed.aap9927 PMC5959841

[44] Geimer Le Lay A.S. , Oravecz A., Mastio J., Jung C., Marchal P., Ebel C., Dembele D., Jost B., Le Gras S., Thibault C. et al. The tumor suppressor Ikaros shapes the repertoire of notch target genes in T cells. Sci. Signal. 2014; 7:ra28. 24643801 10.1126/scisignal.2004545

[45] Ng S.Y. , Yoshida T., Zhang J., Georgopoulos K. Genome-wide lineage-specific transcriptional networks underscore Ikaros-dependent lymphoid priming in hematopoietic stem cells. Immunity. 2009; 30:493–507. 19345118 10.1016/j.immuni.2009.01.014 PMC3012962

[46] Zhang J. , Jackson A.F., Naito T., Dose M., Seavitt J., Liu F., Heller E.J., Kashiwagi M., Yoshida T., Gounari F. et al. Harnessing of the nucleosome-remodeling-deacetylase complex controls lymphocyte development and prevents leukemogenesis. Nat. Immunol. 2011; 13:86–94. 22080921 10.1038/ni.2150 PMC3868219

[47] Bottardi S. , Mavoungou L., Milot E. IKAROS: a multifunctional regulator of the polymerase II transcription cycle. Trends Genet. 2015; 31:500–508. 26049627 10.1016/j.tig.2015.05.003

[48] Davis K.L. Ikaros: master of hematopoiesis, agent of leukemia. Ther. Adv. Hematol. 2011; 2:359–368. 23556102 10.1177/2040620711412419 PMC3573420

[49] Marke R. , van Leeuwen F.N., Scheijen B. The many faces of IKZF1 in B-cell precursor acute lymphoblastic leukemia. Haematologica. 2018; 103:565–574. 29519871 10.3324/haematol.2017.185603 PMC5865415

[50] Schwickert T.A. , Tagoh H., Gultekin S., Dakic A., Axelsson E., Minnich M., Ebert A., Werner B., Roth M., Cimmino L. et al. Stage-specific control of early B cell development by the transcription factor Ikaros. Nat. Immunol. 2014; 15:283–293. 24509509 10.1038/ni.2828 PMC5790181

[51] Oravecz A. , Apostolov A., Polak K., Jost B., Le Gras S., Chan S., Kastner P. Ikaros mediates gene silencing in T cells through Polycomb repressive complex 2. Nat. Commun. 2015; 6:8823. 26549758 10.1038/ncomms9823 PMC4667618

[52] Powell M.D. , Read K.A., Sreekumar B.K., Oestreich K.J. Ikaros zinc finger transcription factors: regulators of cytokine signaling pathways and CD4(+) T helper cell differentiation. Front. Immunol. 2019; 10:1299. 31244845 10.3389/fimmu.2019.01299 PMC6563078

[53] Palstra R.J. , de Crignis E., Roling M.D., van Staveren T., Kan T.W., van Ijcken W., Mueller Y.M., Katsikis P.D., Mahmoudi T. Allele-specific long-distance regulation dictates IL-32 isoform switching and mediates susceptibility to HIV-1. Sci. Adv. 2018; 4:e1701729. 29507875 10.1126/sciadv.1701729 PMC5833994

[54] Boutboul D. , Kuehn H.S., Van de Wyngaert Z., Niemela J.E., Callebaut I., Stoddard J., Lenoir C., Barlogis V., Farnarier C., Vely F. et al. Dominant-negative IKZF1 mutations cause a T, B, and myeloid cell combined immunodeficiency. J. Clin. Invest. 2018; 128:3071–3087. 29889099 10.1172/JCI98164 PMC6026000

[55] Mullighan C.G. , Su X., Zhang J., Radtke I., Phillips L.A., Miller C.B., Ma J., Liu W., Cheng C., Schulman B.A. et al. Deletion of IKZF1 and prognosis in acute lymphoblastic leukemia. N. Engl. J. Med. 2009; 360:470–480. 19129520 10.1056/NEJMoa0808253 PMC2674612

[56] Sun L. , Liu A., Georgopoulos K. Zinc finger-mediated protein interactions modulate Ikaros activity, a molecular control of lymphocyte development. EMBO J. 1996; 15:5358–5369. 8895580 PMC452279

[57] Li Z. , Perez-Casellas L.A., Savic A., Song C., Dovat S. Ikaros isoforms: the saga continues. World J. Biol. Chem. 2011; 2:140–145. 21765980 10.4331/wjbc.v2.i6.140 PMC3135861

[58] Song C. , Pan X., Ge Z., Gowda C., Ding Y., Li H., Li Z., Yochum G., Muschen M., Li Q. et al. Epigenetic regulation of gene expression by Ikaros, HDAC1 and casein kinase II in leukemia. Leukemia. 2016; 30:1436–1440. 26639180 10.1038/leu.2015.331 PMC4889471

[59] Boehm D. , Jeng M., Camus G., Gramatica A., Schwarzer R., Johnson J.R., Hull P.A., Montano M., Sakane N., Pagans S. et al. SMYD2-Mediated histone methylation contributes to HIV-1 latency. Cell Host Microbe. 2017; 21:569–579. 28494238 10.1016/j.chom.2017.04.011 PMC5490666

[60] Mbonye U. , Karn J. The molecular basis for human immunodeficiency virus latency. Annu. Rev. Virol. 2017; 4:261–285. 28715973 10.1146/annurev-virology-101416-041646

[61] Tyagi M. , Pearson R.J., Karn J. Establishment of HIV latency in primary CD4+ cells is due to epigenetic transcriptional silencing and P-TEFb restriction. J. Virol. 2010; 84:6425–6437. 20410271 10.1128/JVI.01519-09 PMC2903277

[62] Bracken A.P. , Dietrich N., Pasini D., Hansen K.H., Helin K. Genome-wide mapping of Polycomb target genes unravels their roles in cell fate transitions. Genes Dev. 2006; 20:1123–1136. 16618801 10.1101/gad.381706 PMC1472472

[63] Malik B. , Hemenway C.S. CBX8, a component of the Polycomb PRC1 complex, modulates DOT1L-mediated gene expression through AF9/MLLT3. FEBS Lett. 2013; 587:3038–3044. 23891621 10.1016/j.febslet.2013.07.034 PMC3800029

[64] Vidal M. , Starowicz K. Polycomb complexes PRC1 and their function in hematopoiesis. Exp. Hematol. 2017; 48:12–31. 28087428 10.1016/j.exphem.2016.12.006

[65] Chevalier N. , Jarrossay D., Ho E., Avery D.T., Ma C.S., Yu D., Sallusto F., Tangye S.G., Mackay C.R. CXCR5 expressing human central memory CD4 T cells and their relevance for humoral immune responses. J. Immunol. 2011; 186:5556–5568. 21471443 10.4049/jimmunol.1002828

[66] Schafer P.H. , Ye Y., Wu L., Kosek J., Ringheim G., Yang Z., Liu L., Thomas M., Palmisano M., Chopra R. Cereblon modulator iberdomide induces degradation of the transcription factors Ikaros and Aiolos: immunomodulation in healthy volunteers and relevance to systemic lupus erythematosus. Ann. Rheum. Dis. 2018; 77:1516–1523. 29945920 10.1136/annrheumdis-2017-212916 PMC6161670

[67] Kronke J. , Udeshi N.D., Narla A., Grauman P., Hurst S.N., McConkey M., Svinkina T., Heckl D., Comer E., Li X. et al. Lenalidomide causes selective degradation of IKZF1 and IKZF3 in multiple myeloma cells. Science. 2014; 343:301–305. 24292625 10.1126/science.1244851 PMC4077049

[68] Diaz T. , Rodriguez V., Lozano E., Mena M.P., Calderon M., Rosinol L., Martinez A., Tovar N., Perez-Galan P., Blade J. et al. The BET bromodomain inhibitor CPI203 improves lenalidomide and dexamethasone activity in in vitro and in vivo models of multiple myeloma by blockade of Ikaros and MYC signaling. Haematologica. 2017; 102:1776–1784. 28751557 10.3324/haematol.2017.164632 PMC5622862

[69] Moros A. , Rodriguez V., Saborit-Villarroya I., Montraveta A., Balsas P., Sandy P., Martinez A., Wiestner A., Normant E., Campo E. et al. Synergistic antitumor activity of lenalidomide with the BET bromodomain inhibitor CPI203 in bortezomib-resistant mantle cell lymphoma. Leukemia. 2014; 28:2049–2059. 24721791 10.1038/leu.2014.106

[70] Bjorklund C.C. , Kang J., Amatangelo M., Polonskaia A., Katz M., Chiu H., Couto S., Wang M., Ren Y., Ortiz M. et al. Iberdomide (CC-220) is a potent cereblon E3 ligase modulator with antitumor and immunostimulatory activities in lenalidomide- and pomalidomide-resistant multiple myeloma cells with dysregulated CRBN. Leukemia. 2020; 34:1197–1201. 31719682 10.1038/s41375-019-0620-8 PMC7214241

[71] Fecteau J.F. , Corral L.G., Ghia E.M., Gaidarova S., Futalan D., Bharati I.S., Cathers B., Schwaederle M., Cui B., Lopez-Girona A. et al. Lenalidomide inhibits the proliferation of CLL cells via a cereblon/p21(WAF1/Cip1)-dependent mechanism independent of functional p53. Blood. 2014; 124:1637–1644. 24990888 10.1182/blood-2014-03-559591 PMC4155272

[72] Fuchs O. Treatment of lymphoid and myeloid malignancies by immunomodulatory drugs. Cardiovasc. Hematol. Disord. Drug Targets. 2019; 19:51–78. 29788898 10.2174/1871529X18666180522073855

[73] Gandhi A.K. , Kang J., Havens C.G., Conklin T., Ning Y., Wu L., Ito T., Ando H., Waldman M.F., Thakurta A. et al. Immunomodulatory agents lenalidomide and pomalidomide co-stimulate T cells by inducing degradation of T cell repressors Ikaros and Aiolos via modulation of the E3 ubiquitin ligase complex CRL4(CRBN.). Br. J. Haematol. 2014; 164:811–821. 24328678 10.1111/bjh.12708 PMC4232904

[74] Polizzotto M.N. , Uldrick T.S., Wyvill K.M., Aleman K., Peer C.J., Bevans M., Sereti I., Maldarelli F., Whitby D., Marshall V. et al. Pomalidomide for symptomatic kaposi's sarcoma in people with and without HIV infection: a phase I/II study. J. Clin. Oncol. 2016; 34:4125–4131. 27863194 10.1200/JCO.2016.69.3812 PMC5477825

[75] Bobardt M. , Kuo J., Chatterji U., Chanda S., Little S.J., Wiedemann N., Vuagniaux G., Gallay P.A. The inhibitor apoptosis protein antagonist Debio 1143 is an attractive HIV-1 latency reversal candidate. PLoS One. 2019; 14:e0211746. 30716099 10.1371/journal.pone.0211746 PMC6361451

[76] Nguyen K. , Das B., Dobrowolski C., Karn J. Multiple histone lysine methyltransferases are required for the establishment and maintenance of HIV-1 latency. Mbio. 2017; 8:e00133-17. 28246360 10.1128/mBio.00133-17 PMC5347344

[77] Turner A.W. , Dronamraju R., Potjewyd F., James K.S., Winecoff D.K., Kirchherr J.L., Archin N.M., Browne E.P., Strahl B.D., Margolis D.M. et al. Evaluation of EED inhibitors as a class of PRC2-Targeted small molecules for HIV latency reversal. ACS Infect. Dis. 2020; 6:1719–1733. 32347704 10.1021/acsinfecdis.9b00514 PMC7359025

[78] Cismasiu V.B. , Paskaleva E., Suman Daya S., Canki M., Duus K., Avram D. BCL11B is a general transcriptional repressor of the HIV-1 long terminal repeat in T lymphocytes through recruitment of the NuRD complex. Virology. 2008; 380:173–181. 18768194 10.1016/j.virol.2008.07.035 PMC2637995

[79] Rathore A. , Iketani S., Wang P., Jia M., Sahi V., Ho D.D. CRISPR-based gene knockout screens reveal deubiquitinases involved in HIV-1 latency in two Jurkat cell models. Sci. Rep. 2020; 10:5350. 32210344 10.1038/s41598-020-62375-3 PMC7093534

[80] Khan S. , Iqbal M., Tariq M., Baig S.M., Abbas W. Epigenetic regulation of HIV-1 latency: focus on polycomb group (PcG) proteins. Clin. Epigenet. 2018; 10:14. 10.1186/s13148-018-0441-z PMC5800276 29441145

[81] Coull J.J. , Romerio F., Sun J.M., Volker J.L., Galvin K.M., Davie J.R., Shi Y., Hansen U., Margolis D.M. The human factors YY1 and LSF repress the human immunodeficiency virus type 1 long terminal repeat via recruitment of histone deacetylase 1. J. Virol. 2000; 74:6790–6799. 10888618 10.1128/jvi.74.15.6790-6799.2000 PMC112196

[82] Goffin V. , Demonte D., Vanhulle C., de Walque S., de Launoit Y., Burny A., Collette Y., Van Lint C. Transcription factor binding sites in the pol gene intragenic regulatory region of HIV-1 are important for virus infectivity. Nucleic Acids Res. 2005; 33:4285–4310. 16061936 10.1093/nar/gki720 PMC1182164

[83] Huang H. , Santoso N., Power D., Simpson S., Dieringer M., Miao H., Gurova K., Giam C.Z., Elledge S.J., Zhu J. FACT proteins, SUPT16H and SSRP1, are transcriptional suppressors of HIV-1 and HTLV-1 that facilitate viral latency. J. Biol. Chem. 2015; 290:27297–27310. 26378236 10.1074/jbc.M115.652339 PMC4646377

[84] Zhao Y. , Karijolich J., Glaunsinger B., Zhou Q. Pseudouridylation of 7SK snRNA promotes 7SK snRNP formation to suppress HIV-1 transcription and escape from latency. EMBO Rep. 2016; 17:1441–1451. 27558685 10.15252/embr.201642682 PMC5048380

[85] Krasnopolsky S. , Kuzmina A., Taube R. Genome-wide CRISPR knockout screen identifies ZNF304 as a silencer of HIV transcription that promotes viral latency. PLoS Pathog. 2020; 16:e1008834. 32956422 10.1371/journal.ppat.1008834 PMC7529202

[86] Li Z. , Wu J., Chavez L., Hoh R., Deeks S.G., Pillai S.K., Zhou Q. Reiterative enrichment and authentication of CRISPRi targets (REACT) identifies the proteasome as a key contributor to HIV-1 latency. PLoS Pathog. 2019; 15:e1007498. 30645648 10.1371/journal.ppat.1007498 PMC6333332

[87] Roling M. , Mollapour Sisakht M., Ne E., Moulos P., Crespo R., Stoszko M., De Crignis E., Bodmer H., Kan T.W., Akbarzadeh M. et al. A two-color haploid genetic screen identifies novel host factors involved in HIV-1 latency. Mbio. 2021; 12:e0298021. 34872356 10.1128/mBio.02980-21 PMC8649776

[88] Bradley T. , Ferrari G., Haynes B.F., Margolis D.M., Browne E.P. Single-Cell analysis of quiescent HIV infection reveals host transcriptional profiles that regulate proviral latency. Cell Rep. 2018; 25:107–117. 30282021 10.1016/j.celrep.2018.09.020 PMC6258175

[89] Golumbeanu M. , Cristinelli S., Rato S., Munoz M., Cavassini M., Beerenwinkel N., Ciuffi A. Single-Cell RNA-Seq reveals transcriptional heterogeneity in latent and reactivated HIV-Infected cells. Cell Rep. 2018; 23:942–950. 29694901 10.1016/j.celrep.2018.03.102

[90] Jager S. , Cimermancic P., Gulbahce N., Johnson J.R., McGovern K.E., Clarke S.C., Shales M., Mercenne G., Pache L., Li K. et al. Global landscape of HIV-human protein complexes. Nature. 2011; 481:365–370. 22190034 10.1038/nature10719 PMC3310911

[91] Pedro K.D. , Agosto L.M., Sewell J.A., Eberenz K.A., He X., Fuxman Bass J.I., Henderson A.J A functional screen identifies transcriptional networks that regulate HIV-1 and HIV-2. Proc. Natl. Acad. Sci. U.S.A. 2021; 118:e2012835118. 33836568 10.1073/pnas.2012835118 PMC7980386

[92] Georgopoulos K. The making of a lymphocyte: the choice among disparate cell fates and the IKAROS enigma. Genes Dev. 2017; 31:439–450. 28385788 10.1101/gad.297002.117 PMC5393059

[93] Lu G. , Middleton R.E., Sun H., Naniong M., Ott C.J., Mitsiades C.S., Wong K.K., Bradner J.E., Kaelin W.G. Jr The myeloma drug lenalidomide promotes the cereblon-dependent destruction of Ikaros proteins. Science. 2014; 343:305–309. 24292623 10.1126/science.1244917 PMC4070318

[94] Haslett P.A. , Hanekom W.A., Muller G., Kaplan G. Thalidomide and a thalidomide analogue drug costimulate virus-specific CD8+ T cells in vitro. J. Infect. Dis. 2003; 187:946–955. 12660941 10.1086/368126

[95] De Keersmaecker B. , Allard S.D., Lacor P., Schots R., Thielemans K., Aerts J.L. Expansion of polyfunctional HIV-specific t cells upon stimulation with mRNA electroporated dendritic cells in the presence of immunomodulatory drugs. J. Virol. 2012; 86:9351–9360. 22718823 10.1128/JVI.00472-12 PMC3416114

[96] Lim H. , Kane L., Schwartz J.B., Hesdorffer C.S., Deeks S.G., Greig N., Ferrucci L., Goetzl E.J. Lenalidomide enhancement of human T cell functions in human immunodeficiency virus (HIV)-infected and HIV-negative CD4 T lymphocytopenic patients. Clin. Exp. Immunol. 2012; 169:182–189. 22774993 10.1111/j.1365-2249.2012.04603.x PMC3406378

[97] Lurain K. , Ramaswami R., Mangusan R., Widell A., Ekwede I., George J., Ambinder R., Cheever M., Gulley J.L., Goncalves P.H. et al. Use of pembrolizumab with or without pomalidomide in HIV-associated non-Hodgkin's lymphoma. J. Immunother. Cancer. 2021; 9:e002097. 33608378 10.1136/jitc-2020-002097 PMC7898875

[98] Martinez V. , Tateo M., Castilla M.A., Melica G., Kirstetter M., Boue F. Lenalidomide in treating AIDS-related Kaposi's sarcoma. AIDS. 2011; 25:878–880. 21412067 10.1097/QAD.0b013e328344c145

[99] Pourcher V. , Desnoyer A., Assoumou L., Lebbe C., Curjol A., Marcelin A.G., Cardon F., Gibowski S., Salmon D., Chennebault J.M. et al. Phase II trial of lenalidomide in HIV-Infected patients with previously treated kaposi's sarcoma: results of the ANRS 154 lenakap trial. AIDS Res. Hum. Retroviruses. 2017; 33:1–10. 27405442 10.1089/AID.2016.0069

