# Supplementary material for: Catchet-MS identifies IKZF1-targeting thalidomide analogues as novel HIV-1 latency reversal agents
Source: Nucleic Acids Res. 2022 May 30;50(10):5577–98. doi: 10.1093/nar/gkac407 (PMC9177988; doi:10.1093/nar/gkac407)
Supplement: gkac407_Supplemental_Files [file gkac407_supplemental_files.zip › Supplementary Material.pdf]

Supplementary Material

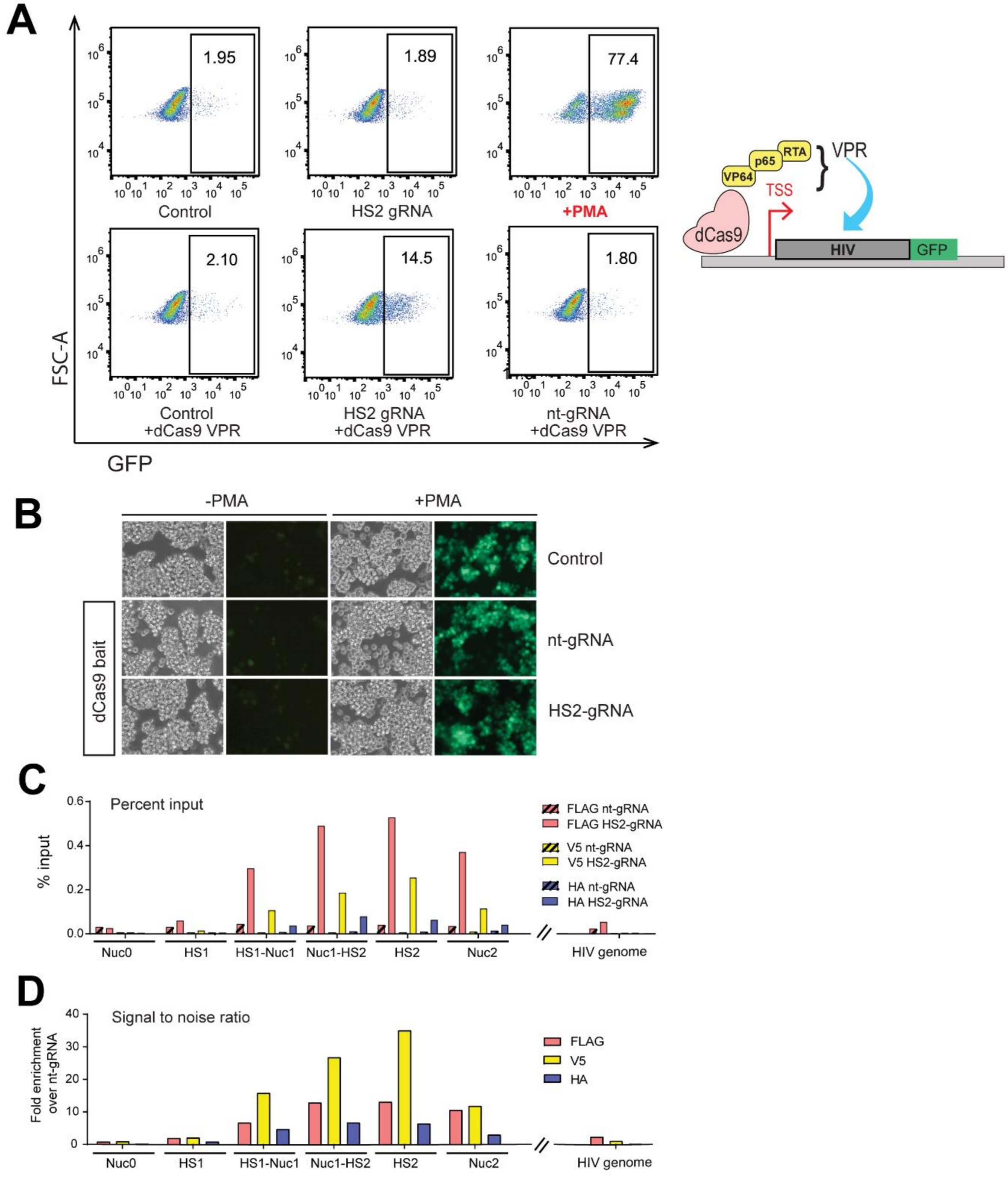

Supplementary Figure S1. Characterization of the experimental system.

(A) Functional validation of the gRNA designed against the HIV-1 5'LTR HS2 region (HS2-gRNA). The FACSs plots show J-Lat 11.1 cells examined by flow-cytometry at 72 hours after nucleofection with a dCas9 VPR construct to check for HIV-1 LTR dependent GFP expression, measured as % GFP positive cells. **(B)** Microscopy pictures (bright field and fluorescence) of control J-lat 11.1 cells, cells expressing dCas9 bait and a non-targeting gRNA, cells expressing the bait and a gRNA targeting the HS2 region. Cells have been examined in unstimulated (-PMA) and in the presence of 20nM PMA (+PMA) to assess reactivation capacity. **(C)** ChIP-qPCR experiments performed using different antibodies, conjugated to affinity beads, against the different synthetic tags (FLAG, V5, HA) of the dCas9 construct. Cells expressing the HS2 gRNA and control cells expressing a non-targeting gRNA (nt-gRNA) are compared. HIV-1 5'LTR sequences recovery is calculated as a percentage of the input. **(D)** From the experiment shown in **(C)**, the signal to noise ratio of the experiment is calculated by dividing the ChIP-qPCR signal obtained in the HS2 gRNA expressing pool with the signal obtained in the nt-gRNA expressing pool. Data are represented as fold enrichment over the non-targeting gRNA signal.

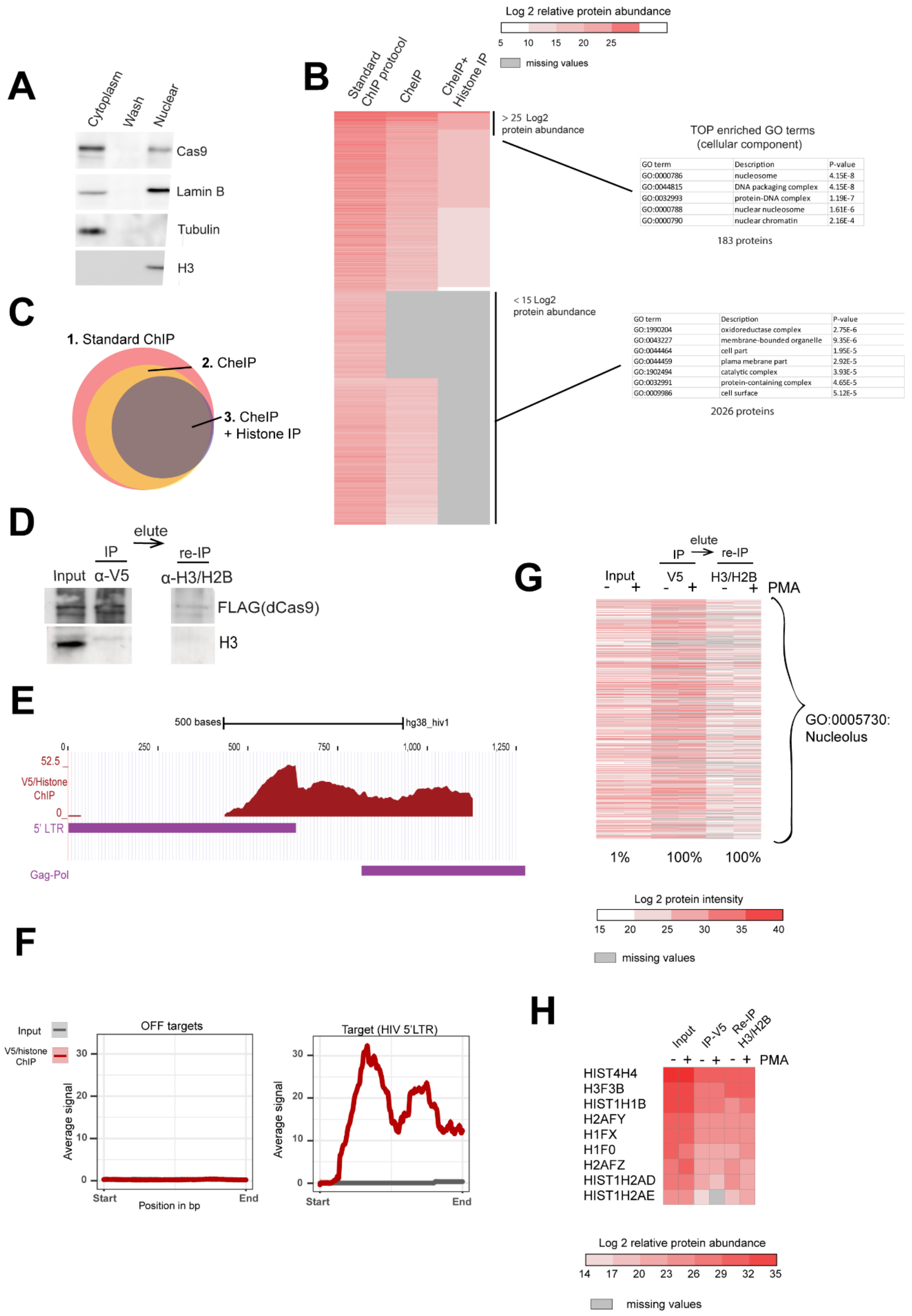

A
Cytoplasm
Wash
Nuclear
Cas9
Lamin B
Tubulin
H3
B
Log 2 relative protein abundance
5 10 15 20 25
missing values
Standard ChIP protocol
CheIP
CheIP+ Histone IP
> 25 Log2 protein abundance
TOP enriched GO terms (cellular component)
GO term | Description | P-value
GO:0000786 | nucleosome | 4.15E-8
GO:0044815 | DNA packaging complex | 4.15E-8
GO:0032993 | protein-DNA complex | 1.19E-7
GO:0000788 | nuclear nucleosome | 1.61E-6
GO:0000790 | nuclear chromatin | 2.16E-4
183 proteins
< 15 Log2 protein abundance
GO term | Description | P-value
GO:1990204 | oxidoreductase complex | 2.75E-6
GO:0043227 | membrane-bounded organelle | 9.35E-6
GO:0044464 | cell part | 1.95E-5
GO:0044459 | plama mebrane part | 2.92E-5
GO:1902494 | catalytic complex | 3.93E-5
GO:0032991 | protein-containing complex | 4.65E-5
GO:0009986 | cell surface | 5.12E-5
2026 proteins
C
1. Standard ChIP
2. CheIP
3. CheIP + Histone IP
D
Input
IP α-V5
elute
re-IP α-H3/H2B
FLAG(dCas9)
H3
E
500 bases
hg38_hiv1
0 250 500 750 1,000 1,250
52.5 _
V5/Histone ChIP
0 _
5' LTR
Gag-Pol
F
Input
V5/histone ChIP
OFF targets
Target (HIV 5'LTR)
Average signal
0 10 20 30
Start
End
Position in bp
G
Input
IP V5
elute
re-IP H3/H2B
- + - + - +
PMA
GO:0005730: Nucleolus
1% 100% 100%
Log 2 protein intensity
15 20 25 30 35 40
missing values
H
Input
IP-V5
Re-IP H3/H2B
- + - + - +
PMA
HIST4H4
H3F3B
HIST1H1B
H2AFY
H1FX
H1F0
H2AFZ
HIST1H2AD
HIST1H2AE
Log 2 relative protein abundance
14 17 20 23 26 29 32 35
missing values

Supplementary Figure S2. Characterization of the chromatin preparation protocol by mass spectometry and depletion of non localized bait.

(A) Western blot analysis using antibody specific for dCas9 indicates localization of HA-V5-FLAG-dCas9 bait following a nuclear and nucleolar fractionation protocol. α-Tubulin is used as a cytoplasmic marker, Histone H3 is used as a chromatin marker while Lamin B is used as a nuclear marker. **(B)** The heatmap shows a comparison between the protein content of a standard ChIP protocol, CheIP, and CheIP followed by a histone enrichment step (CheIP + histone IP) with H2B and H3 conjugated affinity beads. The colors represent the Log2 transformation of the proteins relative abundance. The protein relative abundance was calculated based on protein/peptide spectral intensity values and normalized to the total protein content. **(C)** Venn diagram showing the proportion of the number of hits identified in the different protocols. **(D)** Western blotting with anti Cas9 and anti-V5 antibody indicates relative presence of HA-V5-FLAG-dCas9 bait in the fractions used in the sequential ChIP experiments in Figure 1G. **(E)** ChIP-sequencing tracks of the V5/histone (H3/H2B) sequentially purified chromatin over the HIV-1 5' LTR. **(F)** Average coverage profiles using ChIP sequencing reads mapped 500bp upstream and downstream of the peak center at the 5'LTR region of the HIV genome ('Targets') to the respective coverage around the predicted off targets ('OffTargets'). 'Start' denotes the starting base pair of the aforementioned 1kb region around the peak centers and 'End' the ending base pair respectively. **(G)** Heatmap displaying the content of nucleolar proteins (GO cellular compartment category GO:005730; nucleolus) at each step of the Catchet-MS purification pipeline used for isolation of the HIV-1 5'LTR. The colors range represents the represents the Log2 transformation of the proteins intensities scores. Values corresponding to the V5 based immunopurification are adjusted to the fraction of material analyzed by mass spectrometry, corresponding to 1:40 of the material used for the second, histone based (H2B/H3) immunopurification. Missing values are represented by grey lines. **(H)** Detection of histones. The heatmap summarizes the relative abundance of histone proteins, detected by Catchet-MS. The colors represents the Log2 transformation of the proteins relative abundance. The protein relative abundance was calculated based on protein/peptide spectral intensity values and normalized to the total protein content. Missing values are represented by grey lines.

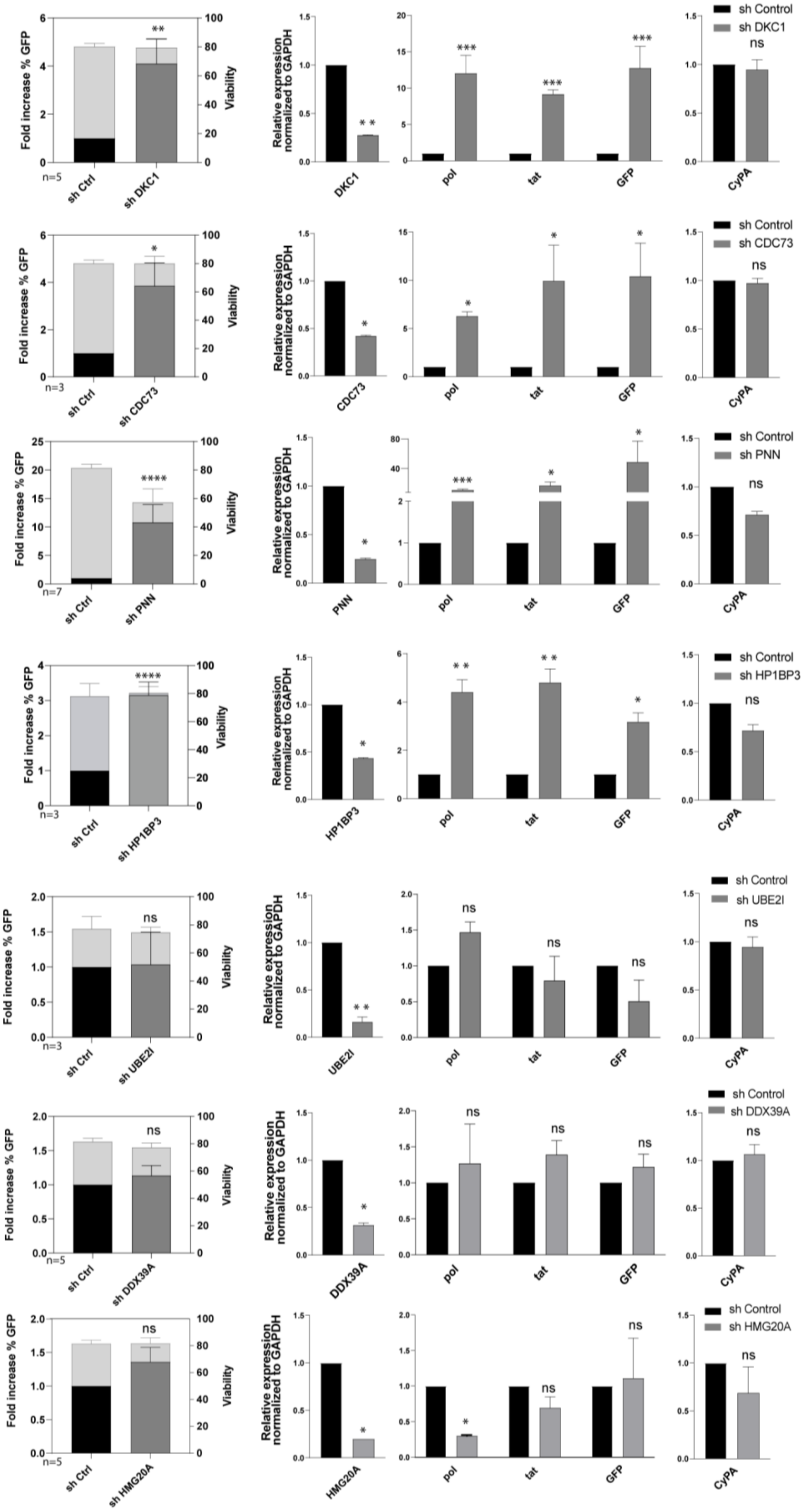

Fold increase % GFP
Viability
Relative expression normalized to GAPDH
n=5
sh Ctrl
sh DKC1
DKC1
pol
tat
GFP
CyPA
sh Control
sh DKC1
n=3
sh CDC73
CDC73
sh Control
sh CDC73
n=7
sh PNN
PNN
sh Control
sh PNN
sh HP1BP3
HP1BP3
sh Control
sh HP1BP3
sh UBE2I
UBE2I
sh Control
sh UBE2I
sh DDX39A
DDX39A
sh Control
sh DDX39A
sh HMG20A
HMG20A
sh Control
sh HMG20A
ns

Supplementary Figure S3. Functional validation of a selection of proteins bound downstream of the latent HIV-1 promoter.

Functional validation of the hits associated with the repressed HIV-1 LTR. shRNA mediated depletion by lentiviral transduction followed by Flow cytometry and RT-PCR. Statistical significance was calculated using unpaired t-test (n ≥ 3, as indicated in the figure) * – p < 0,05, ** – p < 0,01 , *** – p < 0,001, ****– p < 0,0001.

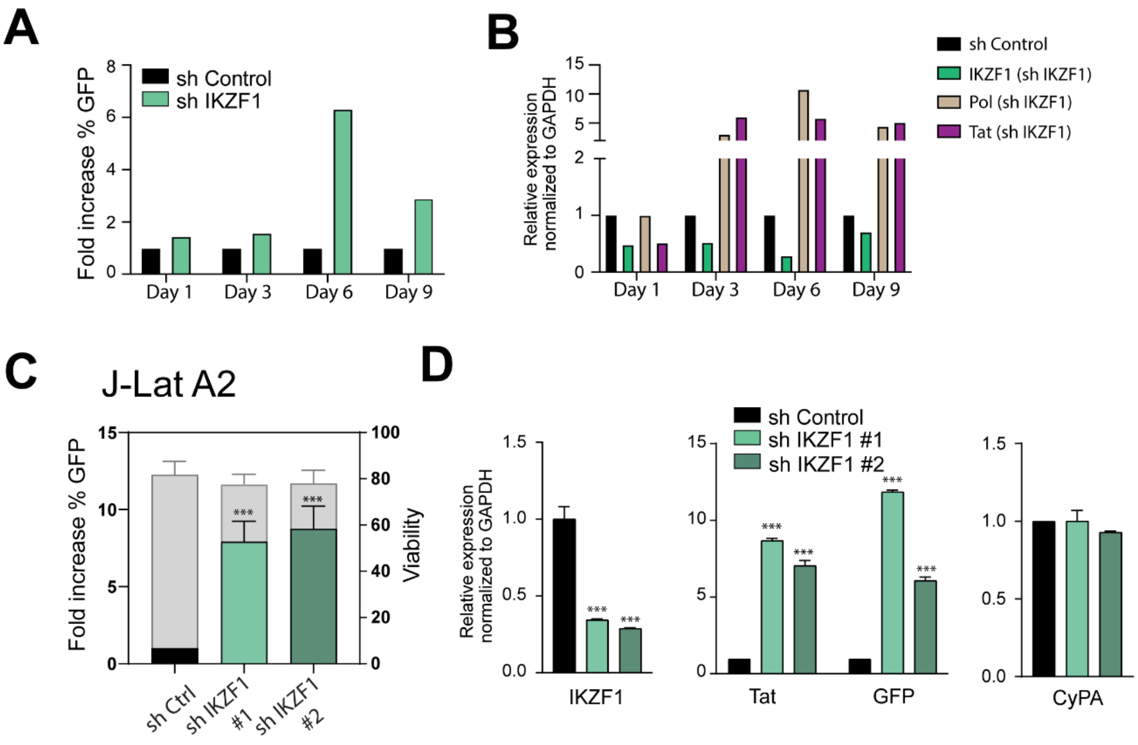

Supplementary Figure S4. shRNA-mediated IKZF1 degradation in J-Lat A2 cells leads to latency reversal.

(A) Fold induction in %GFP in J-Lat 11.1 nucleofected with scramble shRNA (shControl) or shIKZF1 at different timepoints. J-Lat 11.1 were nucleofected with 2 µg of shRNA vector and GFP expression was measured by flow cytometry at different timepoints. **(B)** Gene expression of IKZF1, Pol and Tat in shIKZF1 J-Lat 11.1 compared to shControl cells. J-Lat 11.1 were nucleofected with 2 µg of shRNA vector and gene expression was measured by RT-qPCR at different timepoints. **(C)** Bar plot showing the fold increase in % GFP positive cells (left y-axes) measured by flow cytometry analysis, following IKZF1 depletion in J-Lat A2 cells with two different shRNA constructs (#1 and #2).

The right y-axis represents the percentage of live cells. Data are the mean of two independent experiments (±SD). **(D)** qRT-PCR analysis measuring expression of HIV genes (pol, GFP, tat) in J-lat A2 cells transduced with scramble shRNA (sh Control) and sh IKZF1 #1 and #2. Data, normalized to GAPDH are represented as fold enrichment over sh Control and are the mean of three independent experiments (±SEM). Statistical significance was calculated using an unpaired t test, * – p<0,05; **– p<0,01– ***p<0,001

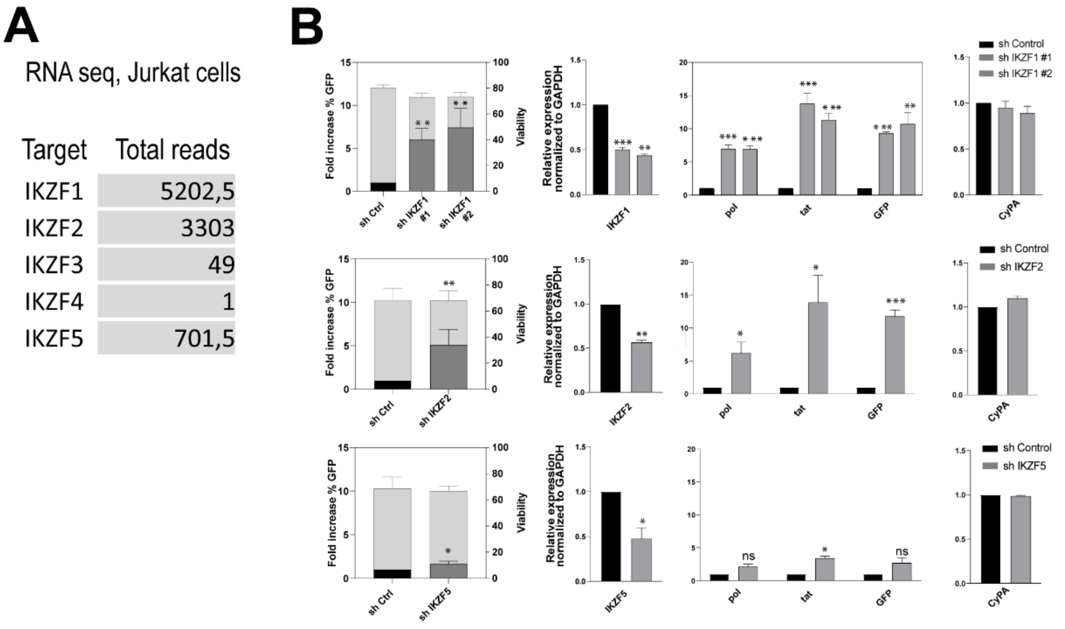

Supplementary Figure S5. Expression of IKZF family members and their role in HIV-1 latency in J-Lat 11.1 cells. **(A)** Expression of IKZF family members in Jurkat cells. The panel shows the number of RNA seq reads in Jurkat cells. The data are published and available in Palstra et al., Science advances (2018). **(B)** IKZF2 and IKZF5, the two prominent Jurkat cells expressed IKZF members were depleted from J-Lat 11.1 cells following shRNA-mediated transduction and GFP expression was examined by Flow cytometry and RT-PCR. Statistical significance was calculated using unpaired t-test * – p < 0,05, ** – p < 0,01 , *** – p < 0,001, ****– p < 0,0001 .

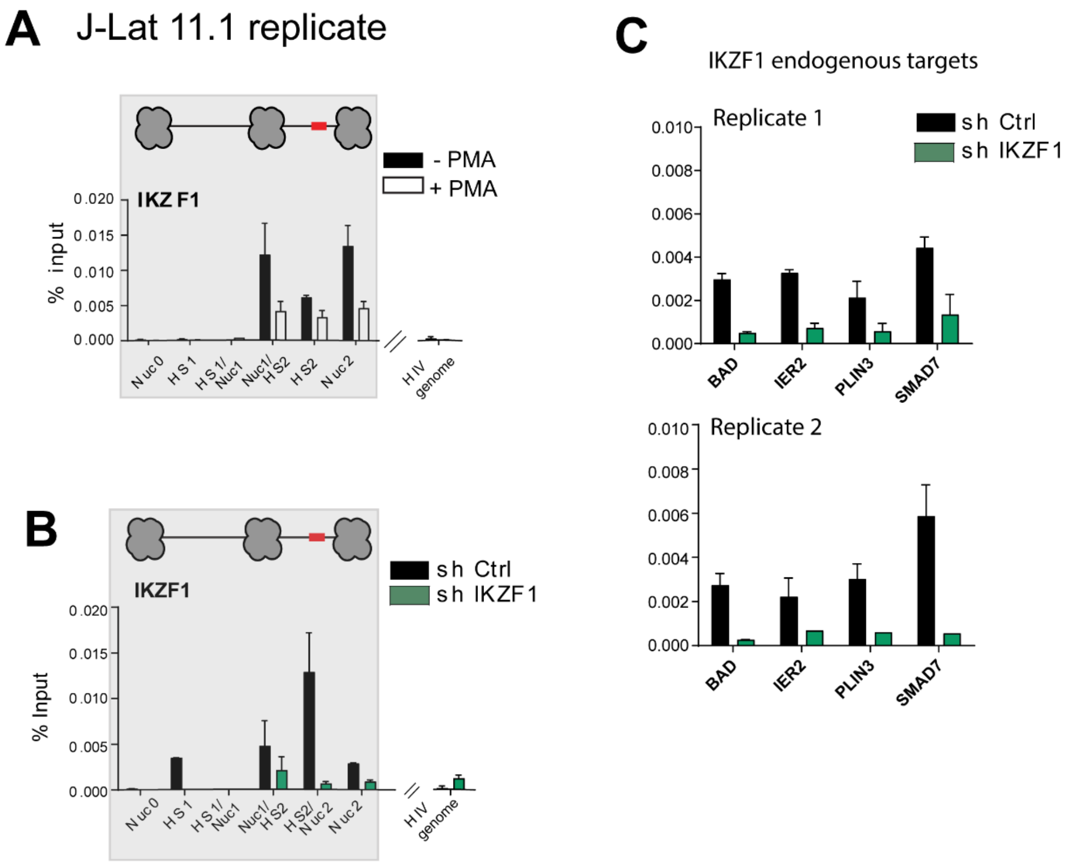

Supplementary Figure S6. Carachterization of IKZF1 binding and chromatin state at the 5'LTR following treatment with PMA and shRNA-mediated IKZF1 degradation in J-Lat 11.1 cells.

(A) Replicate ChIP-qPCR analysis with IKZF1 antibody in latent and PMA stimulated J-Lat 11.1 cells as indicated. Data are presented as % input, error bars represent the standard deviation (SD) of two separate real-time PCR measurements. **(B)** ChIP qPCR analysis using antibody against IKZF1 in J-Lat 11.1 cells transduced with scramble shRNA (shControl) and shIKZF1 probing binding to the HIV-1 5'LTR. Data is presented as % input, error bars represent the standard deviation (SD) of two separate real-time PCR measurements. **(C)** ChIP qPCR analysis using antibody against IKZF1 in J-Lat 11.1 cells transduced with scramble shRNA (shControl) and shIKZF1 probing binding of IKZF1 to endogenous IKZF target genes BAD, IER2, PLIN3 and SMAD7 (58).

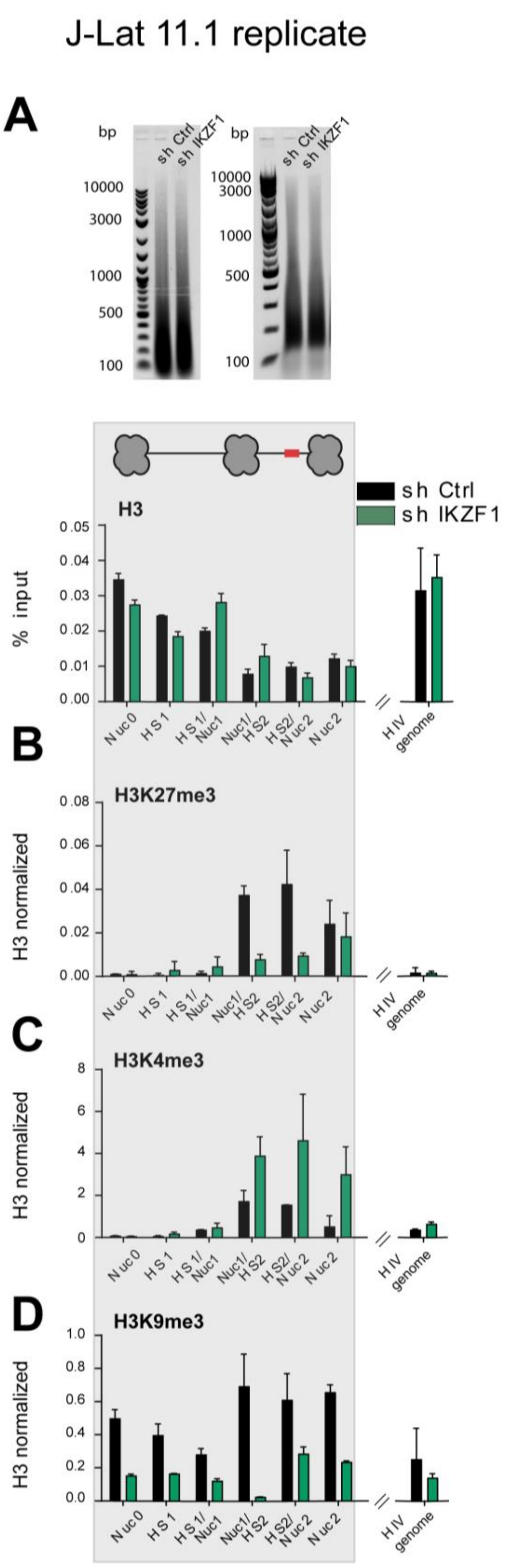
J-Lat 11.1 replicate
A
bp
sh Ctrl
sh IKZF1
10000
3000
1000
500
100
B
C
D
H3
H3K27me3
H3K4me3
H3K9me3
% input
H3 normalized
sh Ctrl
sh IKZF1
Nuc0
HS1
HS1/ Nuc1
Nuc1/ HS2
HS2/ Nuc2
Nuc2
HIV genome

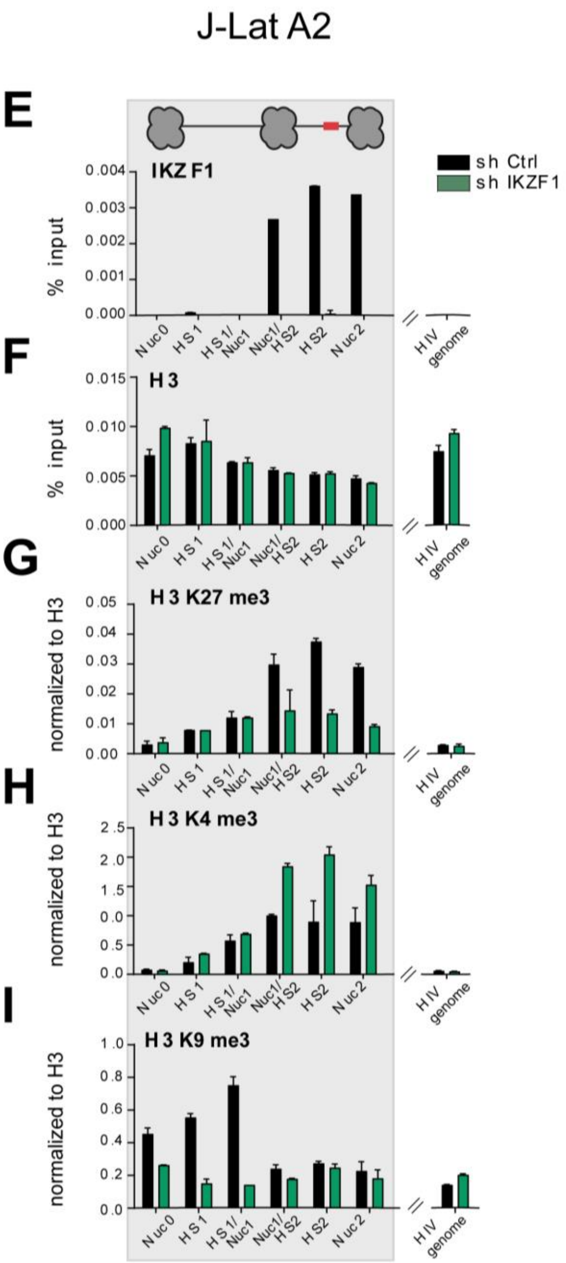
J-Lat A2
E
F
G
H
I
IKZF1
H3
H3K27me3
H3K4me3
H3K9me3
sh Ctrl
sh IKZF1
% input
normalized to H3
Nuc0
HS1
HS1/ Nuc1
Nuc1/ HS2
HS2
Nuc2
HIV genome

Supplementary Figure S7. IKZF1, required for maintenance of HIV-1 latency in J-Lat 11.1 and A2 cells, binds downstream of the latent HIV-1 5'LTR and establishes of a repressive chromatin environment.
(**A-E**) Representative agarose gels demonstrating range of DNA size resulting from sonication of chromatin used in the ChIP experiments presented in the manuscript and ChIP-qPCR using antibodies specific for distinct histone marks in J-Lat 11.1 cells transduced with scramble shRNA (shControl) and shIKZF1; total histone H3 **(A)**, H3K27me3 **(B)**, H3K4me3 **(C)**, H3K9me3 **(D)**. Total histone H3 data **(A)** are represented as % input mean (±SD), histone marks data (**B-D**) are expressed as fold change over H3 signal. Error bars represent the standard deviation (SD) of two separate real-time PCR measurements. **(E-I) (E)** ChIP qPCR analysis with IKZF1 antibody in J-Lat A2 cells transduced with scramble shRNA (shControl) and shIKZF1. Data are presented as % input, error bars represent the standard deviation (SD) of two separate real-time PCR measurements. (**F-I**) Histone marks ChIP qPCR analysis of the HIV-1 5' LTR in J-Lat A2 cells transduced with scramble shRNA (shControl) and shIKZF1; **(F)** Total histone H3 **(G)** H3K27me3 **(H)** H3K4me3 **(I)** H3K9me3. Total histone H3 data **(F)** are presented as % input mean (±SD), histone marks data (**G-I**) are expressed as fold change over H3 signal. Error bars represent the standard deviation (SD) of two separate real-time PCR measurements.

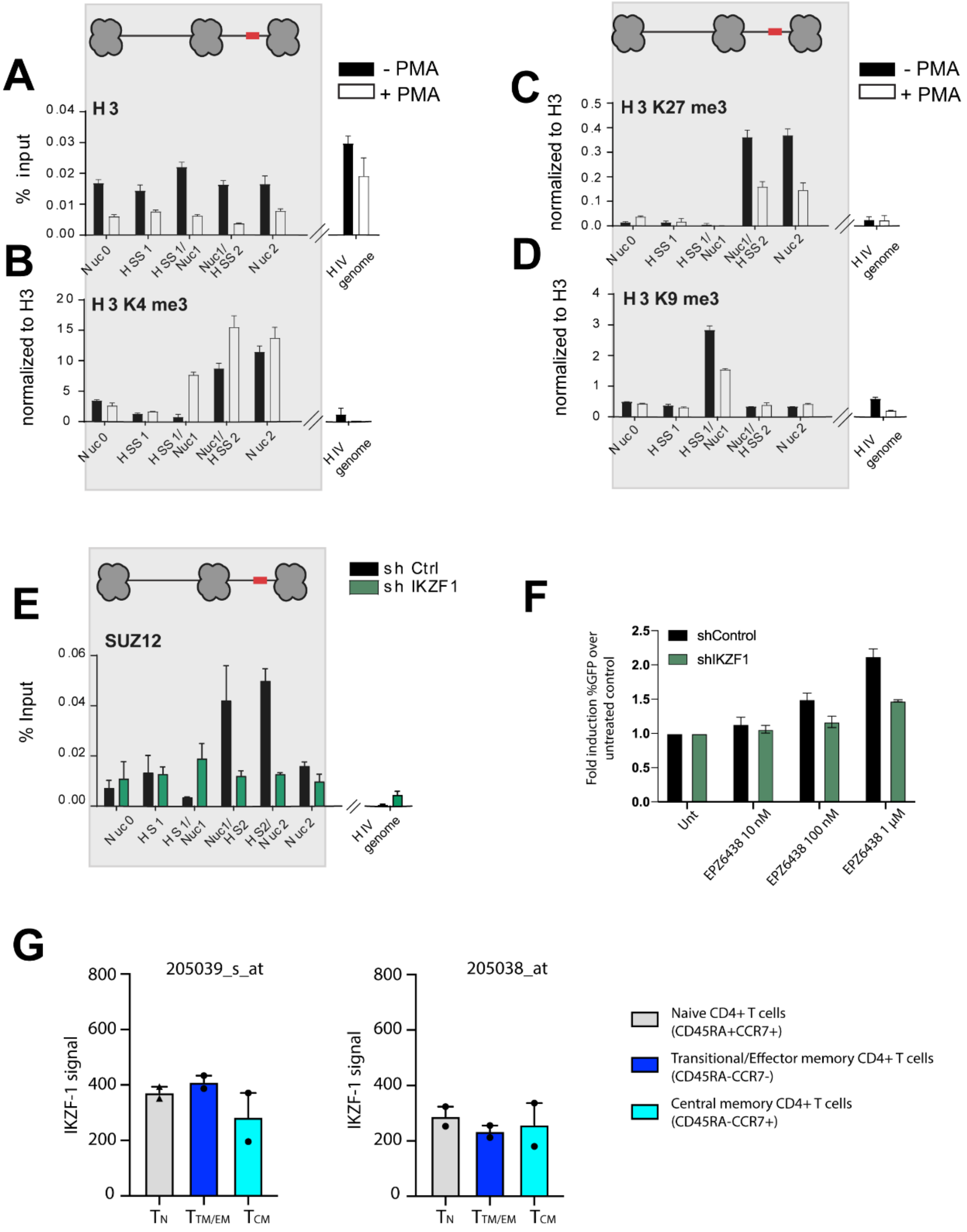

A
B
C
D
E
F
G
- PMA
+ PMA
H 3
% input
H 3 K4 me3
normalized to H3
H 3 K27 me3
H 3 K9 me3
N uc 0
H SS 1
H SS 1/ Nuc1
Nuc1/ HSS 2
N uc 2
H IV genome
sh Ctrl
sh IKZF1
SUZ12
% Input
shControl
shIKZF1
Fold induction %GFP over untreated control
Unt
EPZ6438 10 nM
EPZ6438 100 nM
EPZ6438 1 μM
205039_s_at
205038_at
IKZF-1 signal
Naive CD4+ T cells (CD45RA+CCR7+)
Transitional/Effector memory CD4+ T cells (CD45RA-CCR7-)
Central memory CD4+ T cells (CD45RA-CCR7+)

Supplementary Figure S8. PMA stimulation leads to a loss of repressive chromatin marks and IKZF1 promotes PRC2 recruitment to the latent HIV-1 5’LTR.

(**A-D**) ChIP-qPCR using antibodies specific for distinct histone marks in latent and PMA stimulated J-Lat 11.1 cells as indicated; total histone H3 **(A)**, H3K4me3 **(B)**, H3K27me3 **(C)**, H3K9me3 **(D)**. Total histone H3 data **(A)** are presented as % input mean (±SD), data corresponding to histone marks (**B-D**) are expressed as fold change over H3 signal. Error bars represent the standard deviation (SD) of two separate real-time PCR measurements. **(E)** ChIP-qPCR analysis with SUZ12 in J-Lat 11.1 cells transduced with scramble shRNA (shControl) and shIKZF1 at the HIV-1 5’LTR. Data is presented as % input, error bars represent the standard deviation (SD) of two separate real-time PCR measurements. **(F)** Treatment of J-Lat 11.1 cells transduced with scramble shRNA (shControl) and shIKZF1 with EZH2 inhibitor EPZ6438 for 48 hours as indicated. **(G)** IKZF1 signal in sorted human peripheral blood CD4+ T cell substets. Data was obtained from Array gene expression data generated by Chevalier et al. (2011) in which they isolated human peripheral blood cells from 2 healthy donors and sorted them by flow cytometry to obtain: Naïve (CD4+CD45RA+CCR7+CXCR5-), (2) Central memory (CD4+CD45RA-CCR7+CXCR5-) and (3) Effector memory (CD4+CD45RA-CCR7-CXCR5-). Array gene expression data was generated by RNA extraction from sorted cells and hybridization on Affymetix U133Plus2 chips with 2 different probes (205039_s_at and 205038_at).

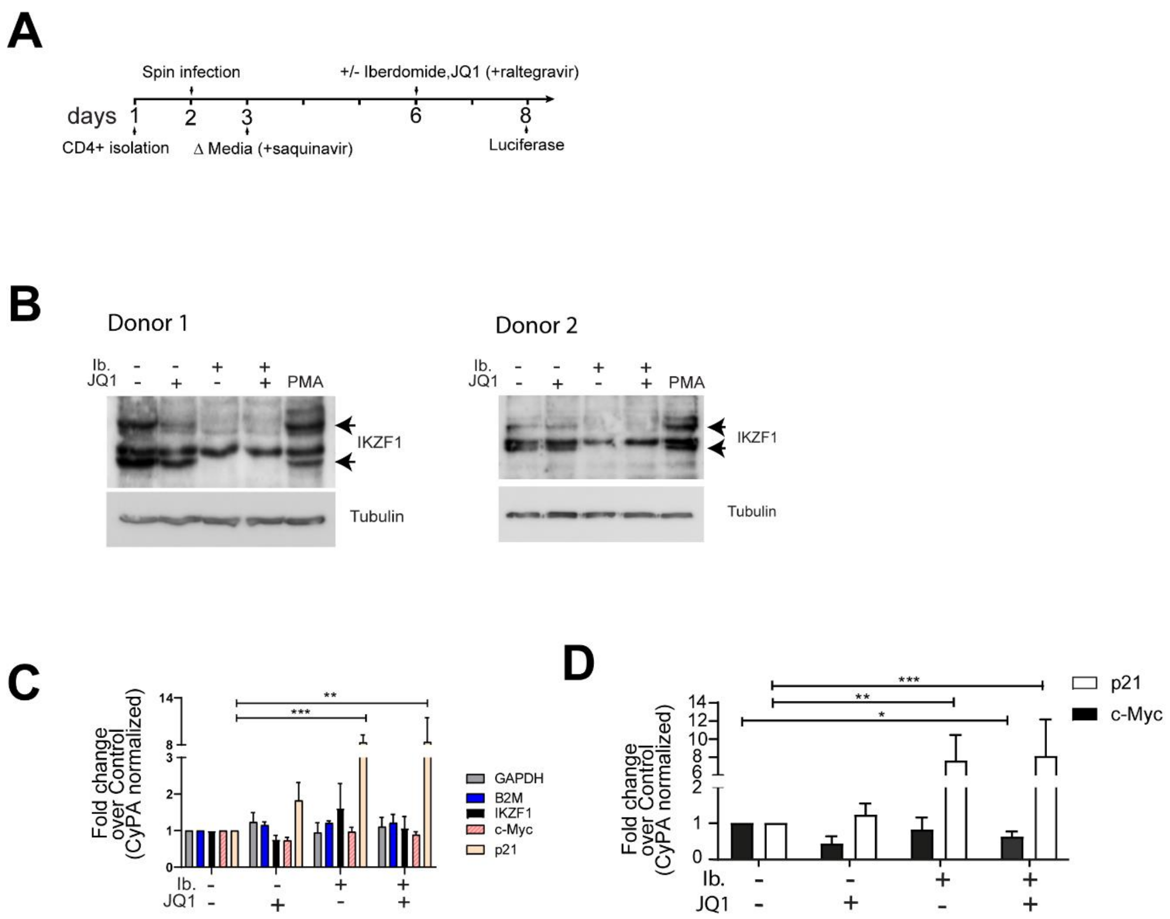

Supplementary Figure S9. Iberdomide treatment of primary CD4+ T cells causes depletion of IKZF at the protein level and does not cause cytotoxicity or induce T cell activation.
(A) Schematic representation of the protocol for HIV-1 latency establishment in primary human CD4+ T cells. **(B)** Western blot analysis using an antibody specific for IKZF1 indicates depletion of IKZF1 at the protein level in CD4+ T cells, upon treatment with iberdomide, JQ1 or a combination of both compounds as indicated for 24h. PMA is used as a control. α-Tubulin is used as a loading control. **(C)** qRT-PCR analysis of IKZF1 and IKZF1 targets p21 and c-myc upon treatment with JQ1 (500nm), iberdomide (10µM), and their combination. RT-PCR was performed in primary CD4+ T cells isolated from three healthy donors. Data are represented as fold change (±SEM) over untreated and are normalized to Cyclophilin A (CyPA). B2M and GAPDH are used as housekeeping genes. Statistical significance was calculated using ratio-paired t-test * – p < 0,05, ** – p < 0,01 , *** – p < 0,001. **(D)** qRT-PCR analysis of IKZF1 targets p21 and c-Myc upon treatment with JQ1 (500nm), iberdomide (10µM), or both compounds. RT-PCR was performed in primary CD4+ T cells isolated from 5 aviremic HIV-1 infected study participants. Data are represented as fold change mean (±SD) over untreated and are normalized with Cyclophilin A (CyPA). Statistical significance was calculated using unpaired t test * – p < 0,05, ** – p < 0,01 , *** – p < 0,001.

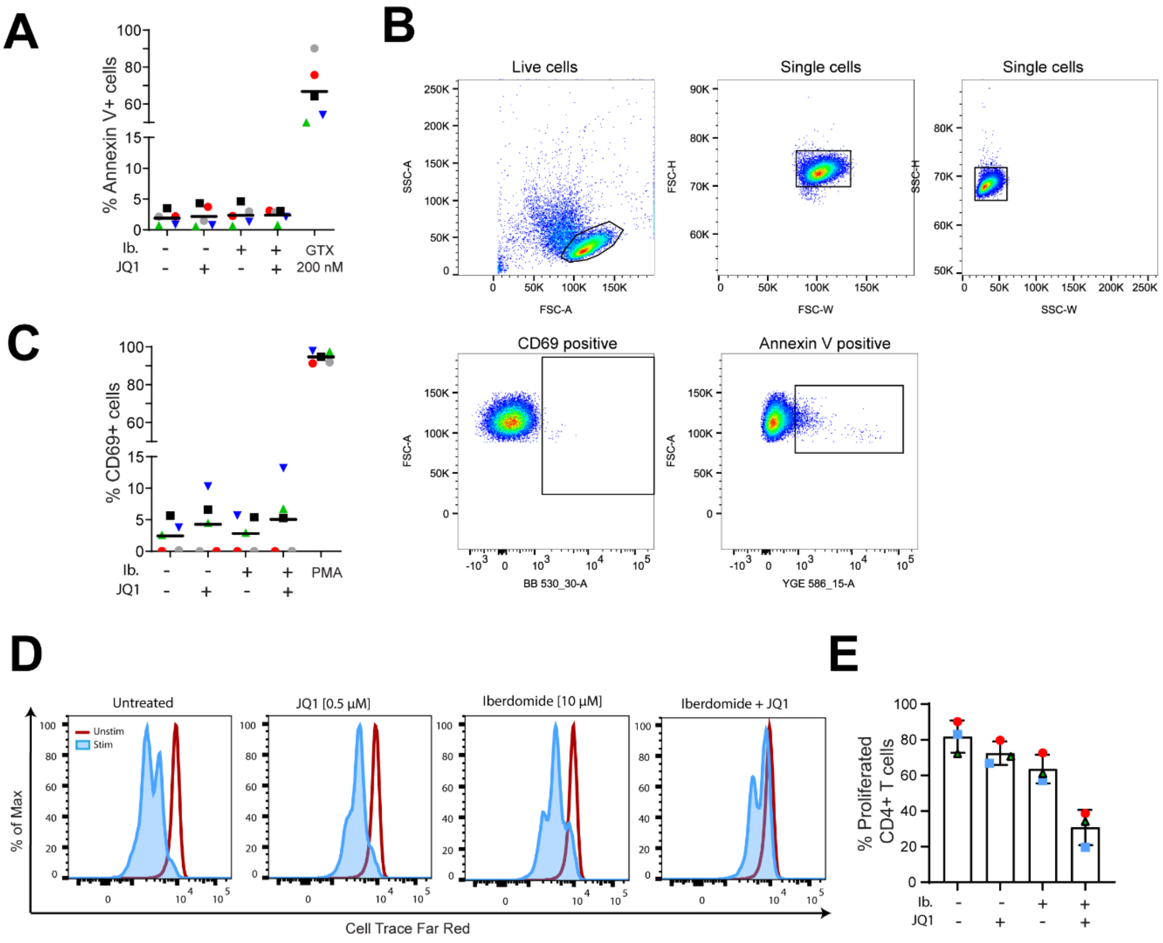

Supplementary Figure S10. Iberdomide treatment causes reduction in proliferation capacity of CD8+ T cells but is not toxic to CD4+ T cells.

(A) Percentage of cells expressing the Annexin V marker of apoptosis in primary CD4+ T cells treated with JQ1 (500nm), iberdomide (10µM), and the combination of both compounds for 48 hours. Treatment with a toxic concentration of Gliotoxin (GTX) 200nM was used as a positive control. **(B)** Representative flow cytometry plots and gating strategy for annexin V and CD69 staining. **(C)** Percentage of cells expressing the CD69 marker of cell activation in primary CD4+ T cells treated with(500nm), iberdomide (10µM), and the combination of both compounds for 48 hours. Experiments were performed in uninfected cells obtained from 5 healthy donors. Treatment with PMA was used as a positive control. Bars represent the average of experiments performed on samples deriving from two healthy donors. **(D)** Representantive histogram of proliferative capacity of unstimulated or aCD3/CD28 stimulated CD4+ T cells in the presence or absence of LRAs. Cells were stained with a proliferation dye and analyzed 72 hours later by flow cytometry. Dividing cells show decreased intensity of proliferation dye as it becomes diluted upon cell division. **(E)** Percentage of proliferated CD4+ T cells from 3 healthy donors as described in C.

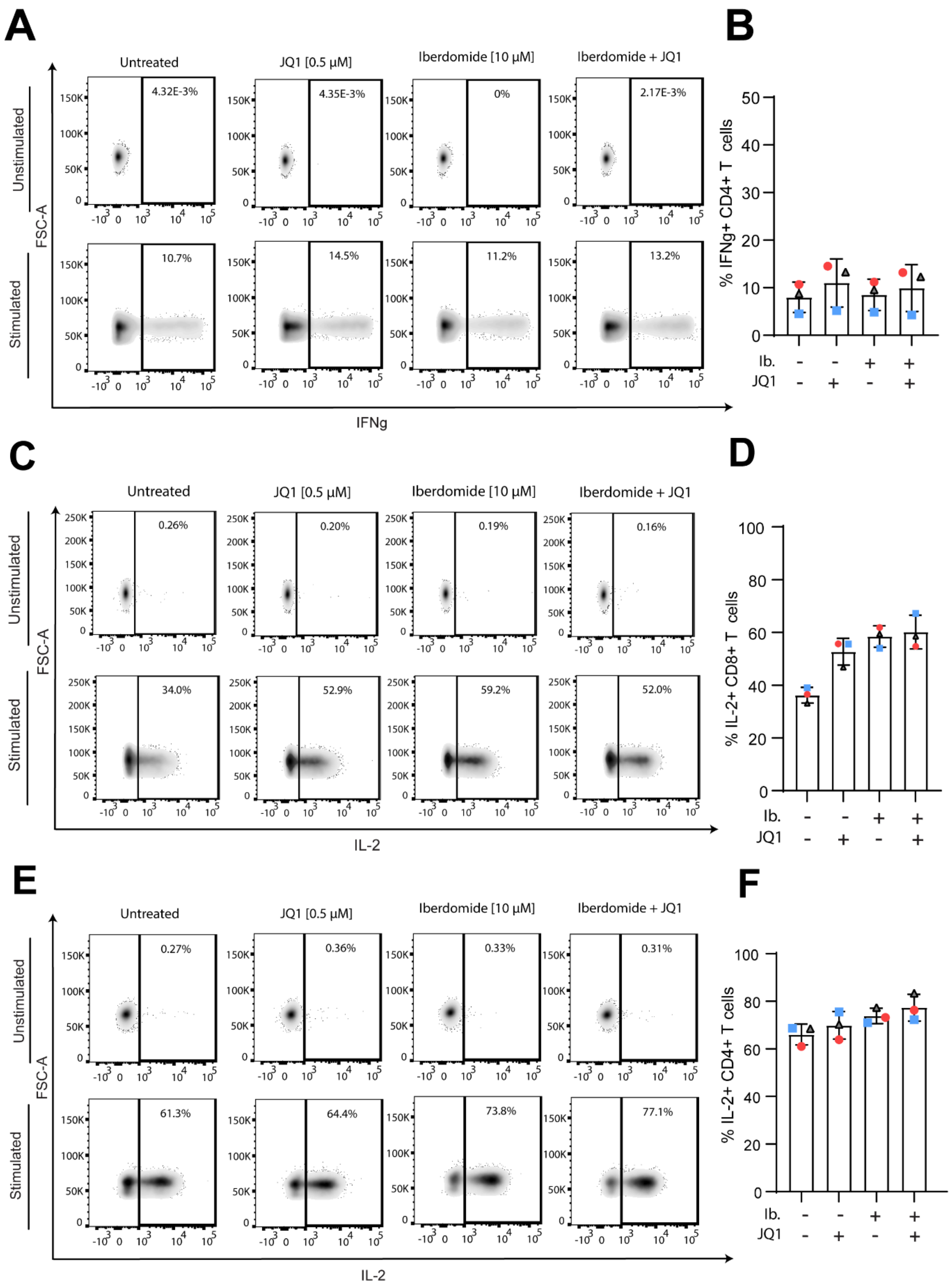
A
Untreated
JQ1 [0.5 μM]
Iberdomide [10 μM]
Iberdomide + JQ1
Unstimulated
Stimulated
FSC-A
4.32E-3%
4.35E-3%
0%
2.17E-3%
10.7%
14.5%
11.2%
13.2%
IFNg
B
% IFNg+ CD4+ T cells
Ib.
JQ1
C
Untreated
JQ1 [0.5 μM]
Iberdomide [10 μM]
Iberdomide + JQ1
0.26%
0.20%
0.19%
0.16%
34.0%
52.9%
59.2%
52.0%
IL-2
D
% IL-2+ CD8+ T cells
E
Untreated
JQ1 [0.5 μM]
Iberdomide [10 μM]
Iberdomide + JQ1
0.27%
0.36%
0.33%
0.31%
61.3%
64.4%
73.8%
77.1%
IL-2
F
% IL-2+ CD4+ T cells

Supplementary Figure S11. Effect of iberdomide treatment alone and in combination with JQ1 on T cell proliferation capacity and effector function

(A) Representative flow cytometry plots (left panel) of INF-g production analysis in unstimulated and stimulated primary CD4+ T cells. Cells were treated as indicated for 18 hours followed by PMA/Ionomycin stimulation for 7 hours in the presence of a protein transport inhibitor or remained unstimulated. IFNg production was assessed by intracellular staining and analyzed by flow cytometry. **(B)** Percentage of INF-g producing CD4+ T cells from 3 healthy donors as described in b. **(C)** Representative flow cytometry plots (left panel) of IL2 production analysis in unstimulated and stimulated primary CD8+ T cells. Cells were treated as indicated for 18 hours followed by PMA/Ionomycin stimulation for 7 hours in the presence of a protein transport inhibitor or remained unstimulated. IL-2 production was assessed by intracellular staining and analyzed by flow cytometry. **(D)** Percentage of IL-2 producing CD8+ T cells from 3 healthy donors as described in C. **(E)** Representative flow cytometry plots (left panel) of IL2 production analysis in unstimulated and stimulated primary CD4+ T cells. Cells were treated as indicated for 18 hours followed by PMA/Ionomycin stimulation for 7 hours in the presence of a protein transport inhibitor or remained unstimulated. IL-2 production was assessed by intracellular staining and analyzed by flow cytometry. **(F)** Percentage of IL-2 producing CD4+ T cells from 3 healthy donors as described in e.

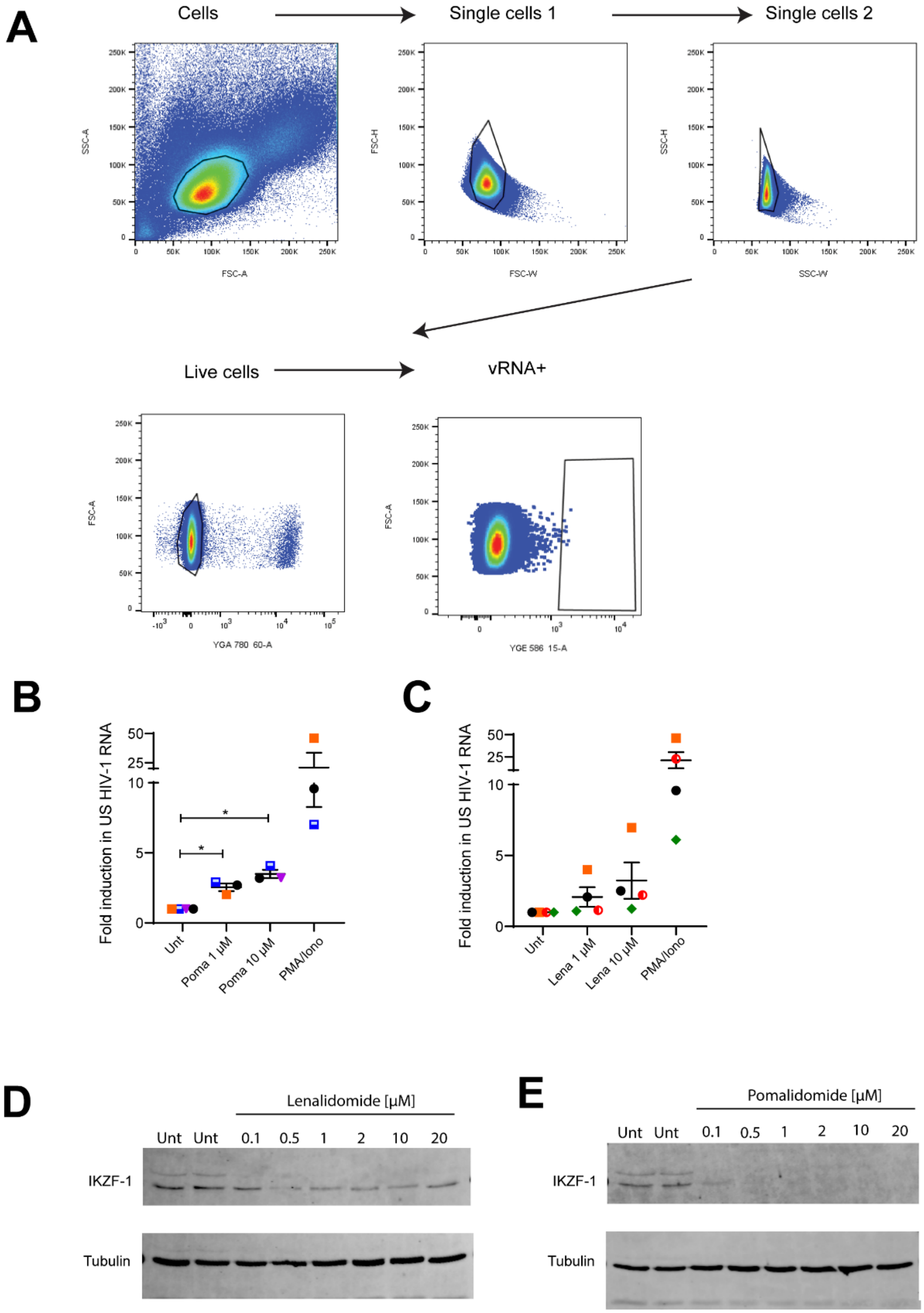
A
Cells
Single cells 1
Single cells 2
SSC-A
FSC-A
FSC-H
FSC-W
SSC-H
SSC-W
Live cells
vRNA+
FSC-A
YGA 780 60-A
FSC-A
YGE 586 15-A
B
Fold induction in US HIV-1 RNA
Unt
Poma 1 μM
Poma 10 μM
PMA/Iono
C
Fold induction in US HIV-1 RNA
Unt
Lena 1 μM
Lena 10 μM
PMA/Iono
D
Lenalidomide [μM]
Unt Unt 0.1 0.5 1 2 10 20
IKZF-1
Tubulin
E
Pomalidomide [μM]
Unt Unt 0.1 0.5 1 2 10 20
IKZF-1
Tubulin

Supplementary Figure S12. FISH-Flow and cell associated HIV-1 RNA analysis of pomalidomide and lenalidomide treated cells from HIV+ infected individuals

(A) Representative FISH-Flow flow cytometry plots and gating strategy for vRNA+ positive cells. (**B-C**) Changes in cell-associated unspliced HIV-1 RNA represented as fold-induction in CD4+ T cells isolated from HIV-1 infected donors after treatment with Pomalidomide **(B)** and Lenalidomide **(C)**. CD4+ T cells were isolated from PBMCs from HIV-1 infected donors and treated as indicated for 24 hours. Statistical significance was calculated using paired two-tailed t test * – p < 0,05. **(D-E)** Western blotting shows protein levels of IKZF1 after 24 hours following treatment with Lenalidomide **(D)** and Pomalidomide (**E**) as indicated in primary CD4+ T cells. α-Tubulin is used as a loading control.

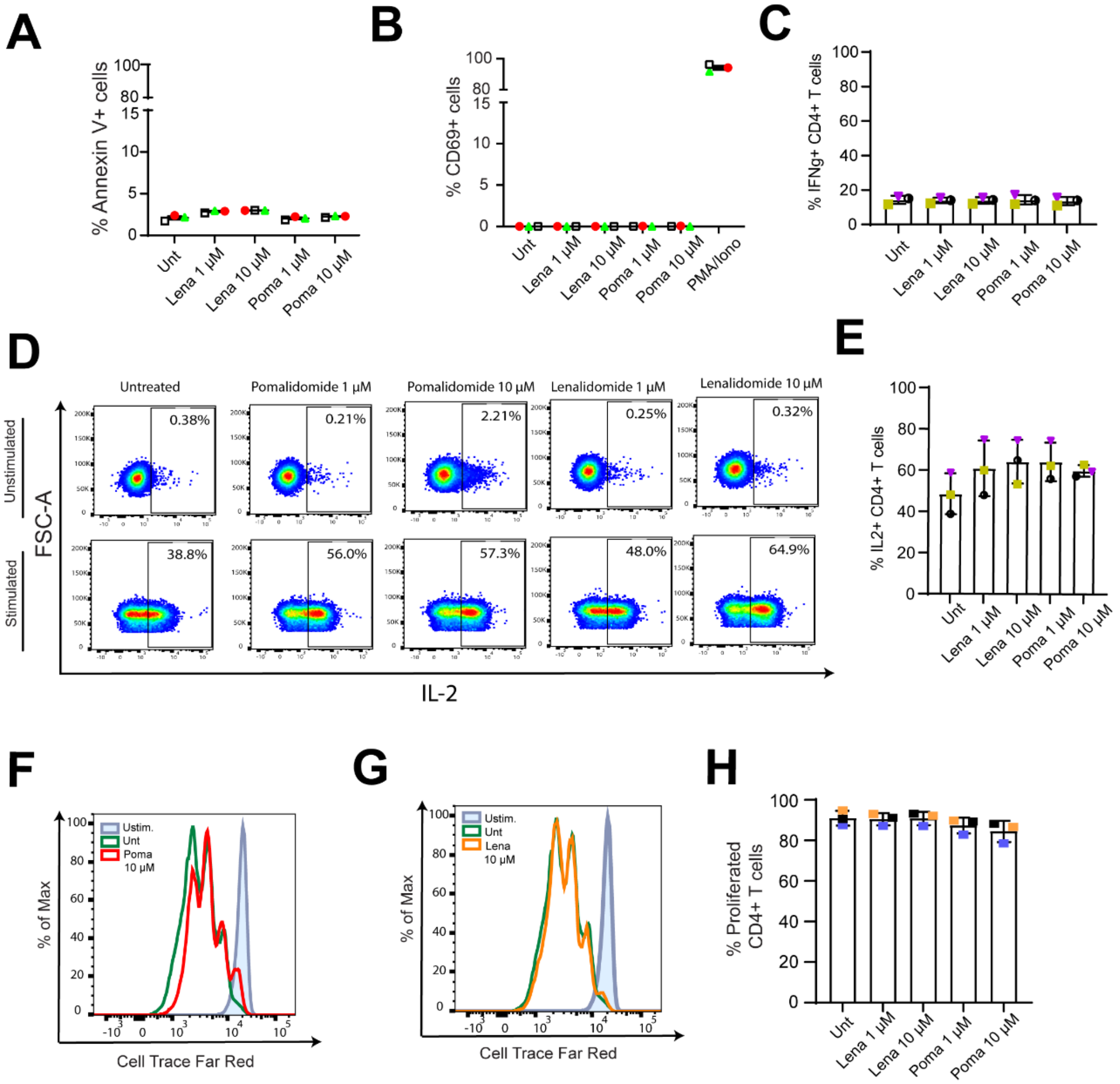
A
% Annexin V+ cells
Unt
Lena 1 μM
Lena 10 μM
Poma 1 μM
Poma 10 μM
B
% CD69+ cells
PMA/Iono
C
% IFNg+ CD4+ T cells
D
Untreated
Pomalidomide 1 μM
Pomalidomide 10 μM
Lenalidomide 1 μM
Lenalidomide 10 μM
Unstimulated
Stimulated
FSC-A
0.38%
0.21%
2.21%
0.25%
0.32%
38.8%
56.0%
57.3%
48.0%
64.9%
IL-2
E
% IL2+ CD4+ T cells
F
Ustim.
Unt
Poma 10 μM
% of Max
Cell Trace Far Red
G
Ustim.
Unt
Lena 10 μM
% of Max
Cell Trace Far Red
H
% Proliferated CD4+ T cells

Supplementary Figure S13. Effect of Lenalidomide and Pomalidomide on toxicity, proliferation and effector capacity of CD4+ T cells from healthy donors

(A) Percentage of cells expressing the Annexin V marker of apoptosis in primary CD4+ T cells treated with Pomalidomide and Lenalidomide for 24 hours. Experiments were performed in uninfected cells obtained from five healthy donors, represented by the dots. **(B)** Percentage of cells expressing the CD69 marker of cell activation in primary CD4+ T cells from 3 healthy donors as described in e. Treatment with PMA/Ionomycin was used as a positive control. (**C**) Cells were treated as indicated for 18 hours followed by PMA/Ionomycin (50 ng / 1 µM) stimulation for 7 hours in the presence of a protein transport inhibitor or remained unstimulated. IFN-g production was assessed by intracellular staining and analyzed by flow cytometry. Numbers in the plot show percentage of IFNg producing cells. (**D**) Representative flow cytometry plots of IL2 production analysis in unstimulated and stimulated primary CD4+ T cells after treatment with Pomalidomide and Lenalidomide. as described in C**.** **(E)** Percentage of IL2 producing CD4+ T cells from 3 healthy donors as described in C. (**F-G**) Representantive histogram of proliferative capacity of unstimulated or aCD3/CD28 stimulated CD4+ T cells in the presence or absence of Pomalidomide (**F**) or Lenalidomide (**G**). Cells were stained with a proliferation dye and analyzed 72 hours later by flow cytometry. Dividing cells show decreased intensity of proliferation dye as it becomes diluted upon cell division. (**H**) Percentage of proliferated CD4+ T cells from 3 healthy donors in the presence of Pomalidomide and Lenalidomide as described in **F-G**.

.

Location coordinates	Reads V5/Histone	Reads_INPUT	avg_reads_V5/Histone	avg_reads_INPUT	avg_reads_V5/Histone	avg_reads_INPUT	fold_enrichment	fold_enrichment_norm	significance	fdr	qc_flag	pileup	saturation_treatment	saturation_control	MACS_fe
K03455.1:324-1353	163	0	15.8252	0.09709	15.8252	0	7.34873	7.34873	125.021	120.338	33	68	0.17952	0.0011	48.8854
K03455.1:7469-7950	58	1	12.0332	0.20747	12.0332	0.20747	5.85798	5.85798	59.4773	55.3671	33	44	0.16111	0.00278	24.244
chr1:163642157-163642744	52	0	8.84354	0.17007	8.84354	0	5.70044	5.70044	69.5395	65.3325	33	42	0.11159	0.00215	30.4648
chr13:106929710-106930148	35	0	7.97267	0	7.97267	0	5.12928	5.12928	40.4553	36.5264	33	27	0.11041	0	19.8376
chr18:69452480-69452979	32	0	6.4	0.2	6.4	0	5	5	42.3036	38.3524	33	28	0.08466	0.00265	20.546
chr10:10107793-10108316	30	0	5.72519	0	5.72519	0	4.90689	4.90689	36.4838	32.6128	33	25	0.07463	0	18.2645
K03455.1:4591-5133	29	0	5.3407	0	5.3407	0	4.85798	4.85798	31.4541	27.6399	33	22	0.06888	0	16.2951
chrX:79904028-79904440	25	0	6.05327	0	6.05327	0	4.64386	4.64386	33.2202	29.3807	33	23	0.08591	0	17.0036
chr10:81712946-81713497	49	0	8.87681	0.36232	8.87681	0	4.61471	5.61471	81.8569	77.5484	33	48	0.11395	0.00465	34.7157
chr10:42527461-42527960	47	1	9.4	0.4	9.4	0.2	4.55459	5.55459	65.5112	61.3377	33	40	0.12434	0.00529	29.0479
chr14:27677712-27678319	46	1	7.56579	0.32895	7.56579	0.16447	4.52356	5.52356	54.3442	50.2743	33	41	0.09465	0.00412	22.6278
chr10:67391032-67391407	21	0	5.58511	0.26596	5.58511	0	4.39232	4.39232	24.5825	20.8832	33	18	0.08268	0.00394	13.4612
chr8:83440464-83441020	53	0	9.51526	0.5386	9.51526	0	4.14296	5.72792	88.1367	83.7771	33	51	0.12184	0.0069	36.8412
chr5:45664104-45664588	35	0	7.21649	0.41237	7.21649	0	4.12928	5.12928	49.8391	45.809	33	32	0.09642	0.00551	23.38
chr11:33625473-33625961	35	0	7.15746	0.409	7.15746	0	4.12928	5.12928	42.3036	38.3524	33	28	0.09537	0.00545	20.546
chr2:42995018-42995354	17	1	5.04451	0.29674	5.04451	0.29674	4.08746	4.08746	21.2761	17.6472	33	16	0.07907	0.00465	12.0442
chr4:120251096-120251584	32	0	6.54397	0.409	6.54397	0	4	5	46.0438	42.0515	33	30	0.08719	0.00545	21.963
chr13:67564368-67564889	38	0	7.27969	0.57471	7.27969	0	3.66297	5.24793	53.4639	49.4081	33	35	0.095	0.0075	24.4341
chr5:164248717-164249084	67	1	18.2065	1.63043	18.2065	0.27174	3.48113	6.06609	101.348	96.8835	33	67	0.27236	0.02439	36.6354
chr2:117593970-117594523	44	0	7.94224	0.72202	7.94224	0	3.45943	5.45943	65.5112	61.3377	33	40	0.10185	0.00926	29.0479
chr2:167082840-167083383	31	1	5.69853	0.55147	5.69853	0.18382	3.36923	4.9542	31.4541	27.6399	33	22	0.07346	0.00711	16.2951
chr10:115327573-115327989	49	0	11.7506	1.19904	11.7506	0	3.29278	5.61471	83.9417	79.6163	33	49	0.1661	0.01695	35.4242
chr4:57790810-57791142	19	1	5.70571	0.6006	5.70571	0.3003	3.24793	4.24793	22.9176	19.2508	33	17	0.09005	0.00948	12.7527
chr7:57945316-57945811	37	0	7.45968	0.80645	7.45968	0	3.20945	5.20945	46.0438	42.0515	33	30	0.09893	0.0107	21.963

Supplementary Table S1. dCas9 V5/Histone ChIP sequencing peak calling summary

Category	Function	Protein	HIV interaction
DNA associated	Chromatin remodelling	CHD1L	
	Chromosome organization	H3F3B	Naji et al., 2012
		HMGB3	
		MAD2L1	Liu et al., 2014
		MTF2	
		PCNA	Zhoue and He, 2004
		WDR5	
		HMGA1	
	DNA metabolism	GINS3	
		MPG	Jager et al., 2011
		NUDT16L1	
	Structural	TOP2B	Lokeswara et al., 2013
	Transcription factor	BCLAF1	Zhou et al., 2008
		CBFB	Anderson and Harris 2015
		FLI1	
		GTF2B	Zhang et al., 2000
		GTF2E2	Zhou et al., 1996
		GTF2I	Taylor et al., 2011
		LDB1	
		TAF3	Zhou and Sharp 1995
		TBPL1	
		ZNF48	
		ZNF638	
		ZNF653	
		PRDM8	
	Transcription complex	MED20	Zhou et al., 2008
		PCBP3	Kim et al., 2013
		SSU72	Chen et al., 2014
		TAF15	Kashanchi et al., 1994
	Ubiquitin proteasome system	PSMA6	
		PSMD10	
		RNF114	
		UBA1	
		UBE2S	
		UBE3A	
		USP11	

Category	Protein	HIV interaction
RNA associated	CSTF3	Naji et al., 2012
	NUDT21	Barrero et al., 2013
	RNF40	
	SNRNP70	Naji et al., 2012
	CSTF1	
	CTNNBL1	
	DBR1	
	GPKOW	
	POLR2G	Agostini et al., 1996
	RBM10	
	RBM15B	Uranishi et al., 2009
	RBM27	
	SAFB2	
	SAP18	La Porte et al., 2016
	SLTM	
	SON	Le Sage et al., 2015
	ZRANB2	
	PRPF4	
	RAVER1	
	SNRNP40	Naji et al., 2012
	U2SURP	Jarboui et al., 2012
	NCBP1	
	SNRPE	Naji et al., 2012
	LUC7L	
	RBM5	
	C7orf55-LUC7L2	
	RBM6	Konig et al., 2008
	RNASEH2C	
	RTCA	
	DDX41	
	DHX40	
	GTF2F2	Cicala et al., 2002
	SRSF2	Jarboui et al., 2012
	U2AF2	Luznik et al., 1995
	SF3B1	Kyei et al., 2018
	RBM17	
	SF3B6	
	STAT3	Fan et al., 2015
	ANP32A	Naji et al., 2012
	ANP32E	Naji et al., 2012
	KPNA2	Naji et al., 2012
	CPSF1	Milev et al., 2012
	PARK7	Lopez-Huertas et al., 2013
	CPSF3	de la Vega et al., 2007
	MAGOH	
	MAGOHB	Jarboui et al., 2012
	C14orf166	
	FKBP4	

Log 2 relative protein abundance

18 20 22 24 26 28 30 32

missing values

Supplementary Table S2. List of putative factors enriched on the active (+PMA) HIV-1 promoter. The table displays selected and functionally classified hits (n=84) identified by Catchet-MS in the +PMA state.

		Factor	-PMA	+PMA	HIV interaction
DNA associated	Bait	dCas9			
	Chromatin remodeling	EED			Nguyen et al., 2017
		PRMT1			Hassa et al., 2008
		MTA2			Cismasiu et al., 2008
		RBBP4			Knoener et al., 2017
		UHRF1			Verdikt et al., 2018
		CHD4			
		HAT1			Galey et al., 2013
		ACTL6A			
	Chromosome organization	HIST1H1B			
		RPA2			Amacker et al., 1997
		DNMT1			Trejbalova et al., 2016
		RPA1			
		H1F0			
		HMGB1			
	DNA replication	MCM2			López-Huertas et al., 2013
		MCM4			
		MCM5			
		MCM6			Jarboui et al., 2012
		MCM7			Jarboui et al., 2012
		RPA3			Amacker et al., 1997
	Structural	BOLA2B			
		FHL1			
		HMGB2			
		IK			
		RAD52			
		PHF6			
		TOP2A			
		RFC4			
		SMC4			
		SAFB			Milev et al., 2012
		TOP1			Shoya et al., 2003
		XRCC6			Li et al., 2001
	Transcription complex	TCERG1			Coiras et al., 2013
		SSRP1			Huang et al., 2015
	Transcription factor	EDF1			Mariotti et al., 2008
		E4F1			
		RFXAP			
		BCCIP			
		ZC3H8			
		ZFR			Milev et al., 2012
		TFPT			
		CGGBP1			
		LEF1			Sharma et al., 2011
		FUBP3			
		FUBP1			
		TRIM28			Milev et al., 2012
		RB1			Prasad and Shanmgam 1993
	Ubiquitin proteasome system	CAND1			
		USP7			

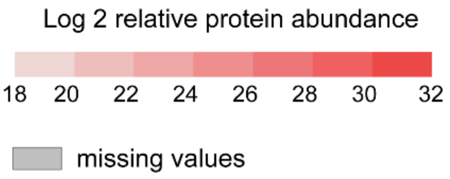

	Factor	-PMA	+PMA	HIV interaction
RNA associated	DNAJC8			
	DDX42			
	ELAVL1			Milev et al., 2012
	HNRNPLL			
	PABPN1			Milev et al., 2012
	PTBP1			
	QKI			
	RBM15			Naji et al., 2011
	RBM39			Naji et al., 2011
	RBM7			
	RBMXL1			
	SNRPA			Simmons et al., 2011
	TARDBP			Ou et al., 1995
	TIA1			
	ZC3H11A			
	ADAR			Jarboui et al., 2012
	DDX39B			Milev et al., 2012
	LUC7L3			
	RBMX			Milev et al., 2012
	SNRNP200			Milev et al., 2012
	SNRPF			Naji et al., 2011
	SNW1			Brès et al., 2005
	EFTUD2			
	SARNP			Lin et al., 2014
	HNRNPDL			
	HNRNPUL2			
	API5			
	FAM98B			Naji et al., 2011
	MYEF2			
	RBM45			
	RNASEH2A			
	SSB			
	STRBP			
	PRPF8			Milev et al., 2012
	SF3A3			Naji et al., 2011
	DHX15			
	SF3B2			
	SRSF4			Milev et al., 2012
	TRA2B			Paz et al., 2014
	KHSRP			Barrero et al., 2013
	PRPF40A			
	PRPF6			Knoener et al., 2017
	DDX46			
	CPSF7			Milev et al., 2012
	KHDRBS3			Kim et al., 2013
	ZNF326			
	MBNL1			
	DAZAP1			Naji et al., 2012
	RALY			
	KHDRBS1			Marsh et al., 2008
	AKAP8L			Jager et al., 2011
	ALYREF			Jager et al., 2011
	TNPO1			Naji et al., 2012
	CPSF6			Sowd et al., 2016
	MATR3			Sarracino et al., 2018
	HNRNPA0			Milev et al., 2012
	SNRNP40			
	SNRPD3			König et al., 2008
	SNRPA1			Lin et al., 2014
	HNRNPUL1			Wagner et al., 2014
	HNRNPH3			Milev et al., 2012
	SNRPB			Fong et al., 2001
	SNRPD2			Fong et al., 2001
	DDX1			Milev et al., 2012
	CELF2			
	UCHL5			Rathore et al., 2020
	RBM12			
	NCOA5			
	U2AF1L5			
	SRSF1			Milev et al., 2012
	SRSF10			Milev et al., 2012
	SRSF6			Swanson et al., 2010

Supplementary Table S3. List of factors enriched on both the latent and active state of the HIV-1 promoter. The table displays selected and functionally classified hits (n=122) identified in both experimental conditions with similar scores, including the HA-V5-FLAG-dCas9 bait and potential non-specific binding contaminants.

Supplementary Table S4. Excel file showing Catchet-MS mass spectrometry raw data and statistics. A key is given for each sample.

Reagent	Source	Identifier
**Antibodies**		
Cas9 antibody (mAb)	Active Motif	61577
Monoclonal ANTI-FLAG_ M2 antibody produced in mouse	Sigma	F3165-.2MG
Mouse monoclonal V5 Tag	Invitrogen	46-0705
HA tag antibody	Abcam	ab18181
Anti Flag M2 affinity gel	Sigma	A-2220
Anti-V5 Agarose Affinity Gel antibody produced in mouse	Sigma	A7345-1ml
Anti-HA Agarose	Pierce	26181
Ikaros (D10E5) Rabbit mAb	Cell Signaling Techn	#CST081119Q
Anti-Ikaros antibody - N-terminal	Abcam	ab229275
anti mouse HRP antibody	Promega	W4021
anti Rabbit HRP antibody	Sigma	A0545
Antibody: donkey anti goat IgG HRP	Promega	V8051
BV510 Human Anti-IL-2 antibody	BD Biosciences	563265
PE-Cy7 Human Anti-IFNg	eBioscience	27-7319-41
IRDye® Donkey 680RD anti-Mouse	LI-COR	926-68072
IRDye® Donkey 680RD anti-Rabbit	LI-COR	926-68073
Anti-Tubulin antibody	Sigma	T5168
**Recombinant DNA**		
sh IKZF1 #1	Sigma	TRCN0000107874
sh IKZF1 #2	Sigma	TRCN0000107871
sh CDC73	Sigma	TRCN0000008728
sh Control	Sigma	SHC002
sh CTR9	Sigma	TRCN0000008739
sh DKC1	Sigma	TRCN0000352996
sh NFRKB	Sigma	TRCN0000014868
sh HP1BP3	Sigma	TRCN0000180320
sh CHD1	Sigma	TRCN0000021309
sh EED	Sigma	TRCN0000021204
sh SIN3A	Sigma	TRCN0000021774
sh SUZ12	Sigma	TRCN0000038724
UBE2I	Sigma	TRCN0000007205
CBX8	Sigma	TRCN0000363282
HMG20A	Sigma	TRCN0000015578

UCHL5	Sigma	TRCN0000234906
RNGTT	Sigma	TRCN0000284836
PRPF6	Sigma	TRCN0000293597
PNN	Sigma	TRCN0000072278
DDX39	Sigma	TRCN0000050668
Lentiviral packaging construct pCMVDR8.91	(Naldini et., 1996)	N/A
VSV-G glycoprotein-expressing vector	(Naldini et., 1996)	N/A
Plasmid: SpCas9n-2A-Puro V2.0	(Ran et al., 2013)	62987

Primer	Application	Forward (5' to 3')	Reverse (5' to 3')
Nuc-0	ChIP-qPCR	CCACACACAAGGCTACTTCC	AACTGGTACTAGCTTGTAGCAC
HS1	ChIP-qPCR	TGTGAGCCTGCATGGGATGG	GAAAGTCCCCAGCGGAAAGT
HS1/Nuc1	ChIP-qPCR	AGTGGCGAGCCCTCAGATG	AGCTTTATTGAGGCTTAAGC
Nuc1/HS2	ChIP-qPCR	CGTCTGTTGTGTGACTCTGGT	TCGAGAGAGCTCCTCTGGTT
Nuc1	ChIP-qPCR	TCTCTGGCTAACTAGGGAACC	AAAGGGTCTGAGGGATCTCTAG
HS2/Nuc2	ChIP-qPCR	GCCCGAACAGGGACTTGAAA	TTGGCGTACTCACCAGTCG
Nuc2/Gag	ChIP-qPCR	GGTGCGAGAGCGTCAGTAT	AGCTCCCTGCTTGCCCATA
HIV-1 genome (Vif)	ChIP-qPCR	GGTCTGCATACAGGAGAAAGAG	TTAGTTGGTCTGCTAGGTCAGG
Cyclophilin A (PPIA)	RT-qPCR	TCATCTGCACTGCCAAGACTG	CATGCCTTCTTTCACTTTGCC
GAPDH	RT-qPCR	CAAGAAGGTGGTGAAGCAG	GCCAAATTCGTTGTCATACC
β2-microglobulin	RT-qPCR	ATGAGTATGCCTGCCGTGTG	CCAAATGCGGCATCTTCAAAC
HIV-1 Pol	RT-qPCR	GGTTTATTACAGGGACAGCAGAGA	ACCTGCCATCTGTTTTCCATA
HIV-1 Tat	RT-qPCR	CAAAAGCCTTAGGCATCTCCT	CCACCTTCTTCTTCGATTCCT
GFP	RT-qPCR	GAAGCAGCACGACTTCTTCAA	GCTTGTCGGCCATGATATAGA
IKZF1	RT-qPCR	GGGTCAAGACATGTCCCAAGT	ACATTACTGGCCACGACTCTG
CDC73	RT-qPCR	GTTTATGTCCGACGTGCAGC	TACTTGCCGATGTTGACGCT
DKC1	RT-qPCR	CACTTACCCTCGGAAGTGGG	TTTTGGCAGACTCACTGTAGTCAA
SUZ12	RT-qPCR	GCCTTTGAGAAGCCAACACAG	AGCTGCAAATGAGCTGACAAG
UBE2I	RT-qPCR	GAAAGGGACTCCGTGGGAAG	GCTTGAGCTGGGTCTTGGAT
PNN	RT-qPCR	AAGAGCGCACACGTAGAGAC	CCAAAAGCCGCAGTTCTGTC
HP1BP3	RT-qPCR	GCCGCCGCCATTACG	ATCTTCTACCTTCTCACCTAACTTG
DDX39A	RT-qPCR	CGGCGGAAAACCGAAGTTGG	GCTGTGGATGGAAACGTAGGA
HMG20A	RT-qPCR	AGCTACACATCACTTGACACCA	ATCTGCAAAAAGGGGCGGTA
IKZF2	RT-qPCR	TTCCTTCCTCCTCCCCTTGA	TGAAAGCTCATTGTCACACGTT
IKZF5	RT-qPCR	GAAGCAGAGGCTCTTCAGGG	TGAGGTTTTTCACCTGTGTGG
p21	RT-qPCR	AGCAGAGGAAGACCATGTGGAC	TTTCGACCCTGAGAGTCTCCAG
c-Myc	RT-qPCR	TCTCCACACATCAGCACAACTACGC	CGCCTCTTGACATTCTCCTCGGTG
Gag Fw/ SK437 Rv	Nested PCR preamp	TCAGCCCAGAAGTAATACCCATGT	TGCTATGTCAGTTCCCCTTGGTTCTCT

Gag Fw/ Gag2 Rv	Nested PCR amp	TCAGCCCAGAAGTAATACCCATGT	CACTGTGTTTAGCATGGTGTTT
Gag Taqman probe	Nested PCR amp	[6FAM]ATTATCAGAAGGAGCCACCCCACAAGA[BHQ1]	
U6 promoter	Sequencing, detect gRNA insertion		ACTATCATATGCTTACCGTAAC

Supplementary Table S5. Reagents and qPCR primer list.
